# Supplementary material for: In-depth characterization of stem cell potency and genotoxicity for clinical-scale ex vivo CRISPR-Cas9 gene editing
Source: Mol Ther Adv. 2026 Jul 22;34(3):201816. doi: 10.1016/j.omta.2026.201816 (PMC13476499; doi:10.1016/j.omta.2026.201816)
Supplement: Supplementary file 1 [file mmc1.pdf]

OMTA, Volume *34*

Supplemental information

In-depth characterization of stem cell potency and genotoxicity for clinical-scale *ex vivo* CRISPR-Cas9 gene editing

Asma Naseem, Winston Vetharoy, Thomas E. Whittaker, Federica Zinghirino, Antonio Carusillo, Roopkatha Ghosh, Eduardo Gomez-Castaneda, Hashim Ali, Carlo Cipriani, Eugenio Montini, Adrian J. Thrasher, Giorgia Santilli, Daniela Cesana, Giandomenico Turchiano, and Alessia Cavazza

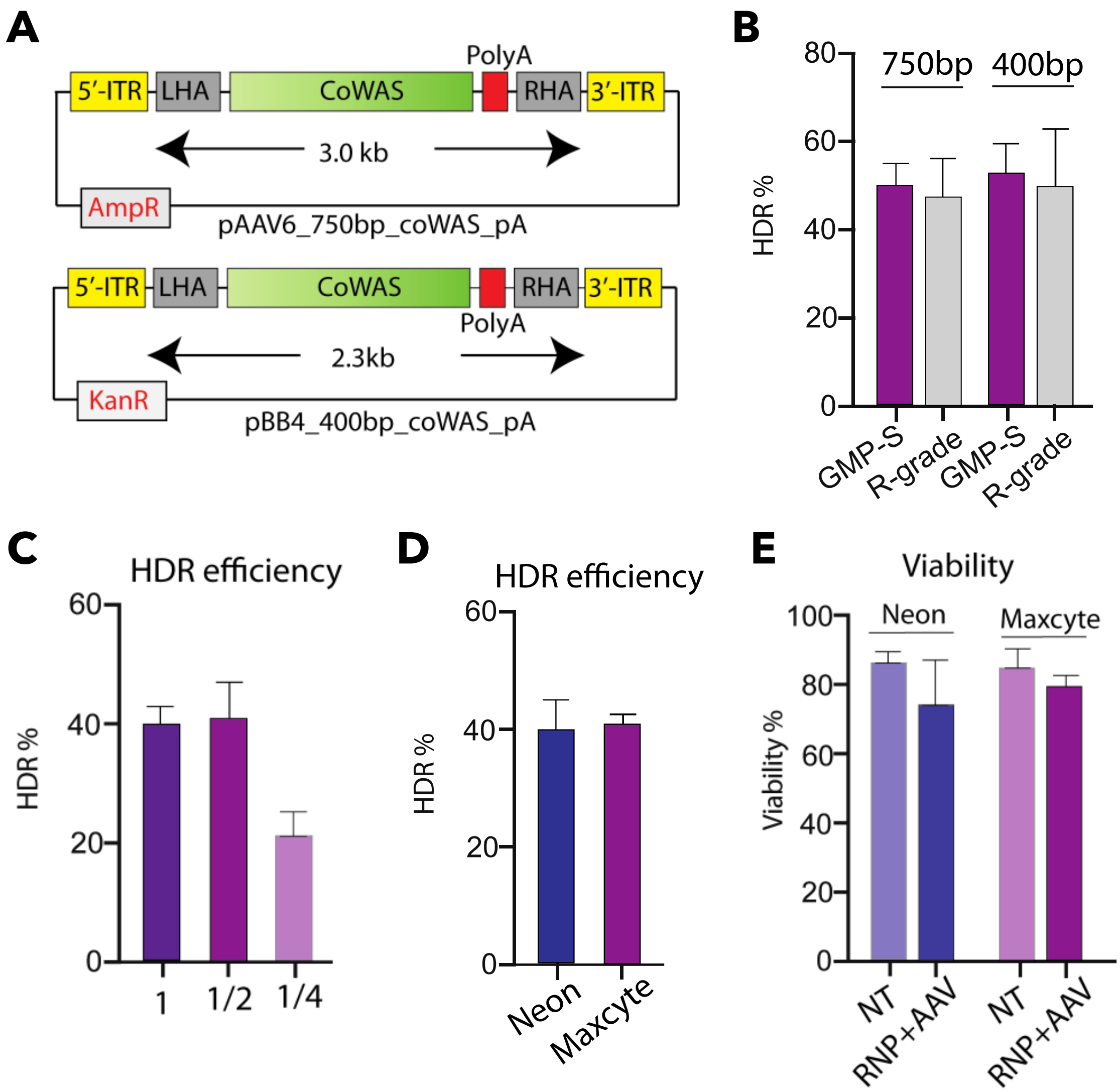

Figure S1. HSPC gene editing scale-up protocol and reagents optimization.
(A) maps of the AAV donor molecules used in the proof-of concept study by Rai et al[1] (pAAV6_750bp_coWAS_pA) and in this study (pBB4_400pb_coWAS_pA). (B) Frequency of coWAS cassette knock-in via HDR in HSPCs (n=3 biological replicates) when using the two different AAV donor vectors (as in (A)) and cGMP- or research-grade reagents. (C) Frequency of coWAS cassette knock-in via HDR in HSPCs (n=3 biological replicates) when using different RNP doses. (D-E) Frequency of coWAS cassette knock-in via HDR in HSPCs (D) and cell viability (E) when electroporating cells with different electroporation machines (n=3 biological replicates). Unmanipulated HSPCs were used as control. Data are presented as mean ± SD.

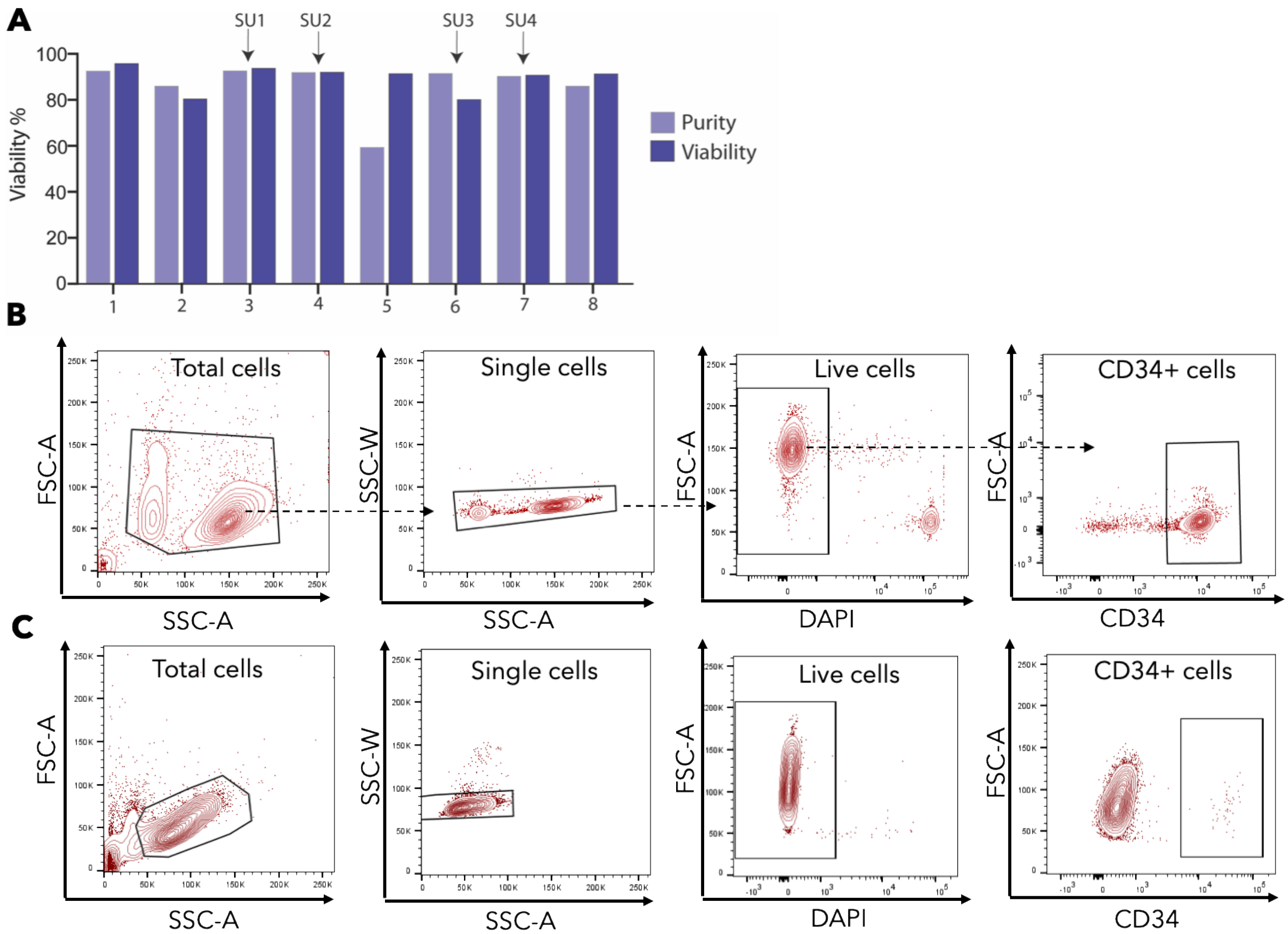

Figure S2: Purity and viability of HSPCs isolated from leukapheresis products (A) CD34+ isolation was carried out from G-CSF mobilised leukapheresis from eight healthy donors. Purity (fraction of CD34+ cells) and viability post selection is shown. Arrows indicate the donors used for the 4 scale ups in this study. (B) Gating strategy used to assess HSPC purity (%CD34+) and viability (DAPI-) in HSPC samples and (C) in unstained controls.

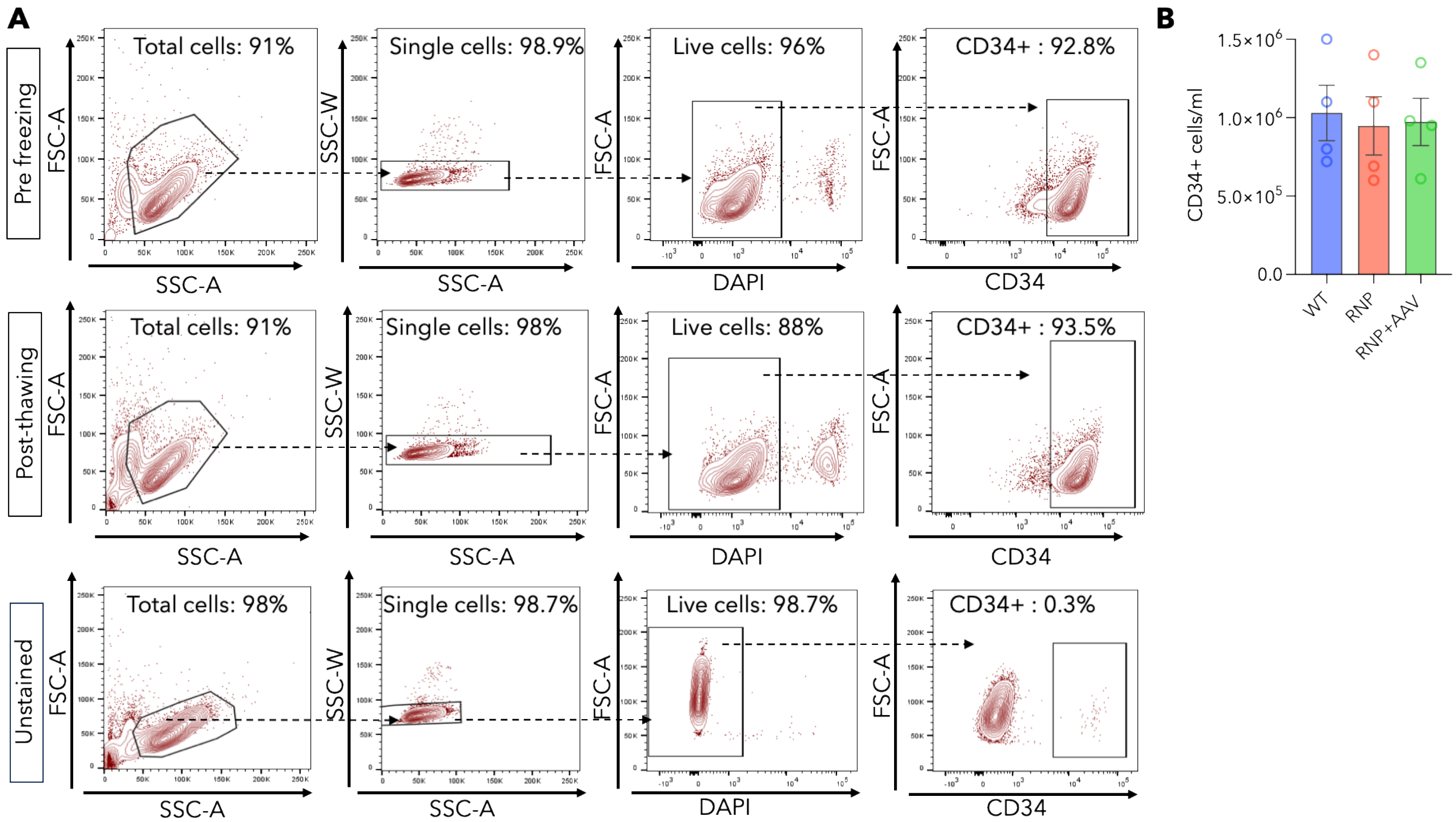

Figure S3: Gating strategy to assess HSPC purity and viability pre- and post-cryopreservation (A) CD34+ cell isolation was carried out from G-CSF mobilised leukapheresis from eight healthy donors. Purity (fraction of CD34+ cells) and viability post selection is shown. Arrows indicate the donors used for the 4 scale ups in this study. (B) Number of CD34+ HSPCs recovered after thawing of the cryopreserved products (number of cells per ml, in 10 ml total; n=4, where each dot represents one donor source). Data are presented as mean ± SD.

Number of mice analysed				
	**SU-1**	**SU-2**	**SU-3**	**SU-4**
**WT**	5	4	2	2
**RNP**	4	4	3	2
**RNP+AAV**	15	16	9	3

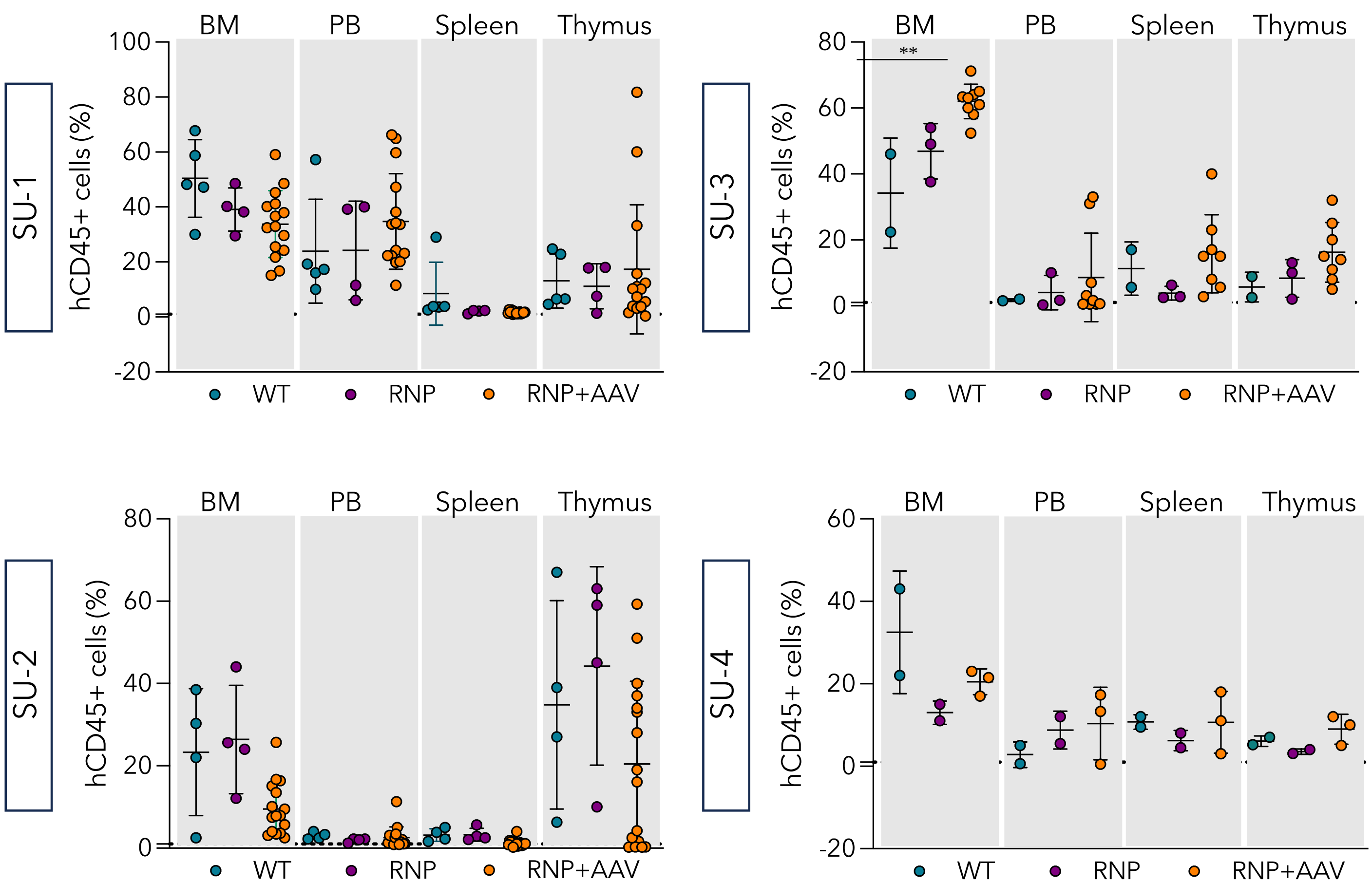

Figure S4: Assessment of human chimerism in xenotransplanted NSG mice. Engraftment frequencies in the bone marrow (BM), peripheral blood (PB), spleen and thymus of transplanted NSG mice at 14 weeks post injection, expressed as the frequency of CD45+ cells detected by flow cytometry, per each single SU product infused

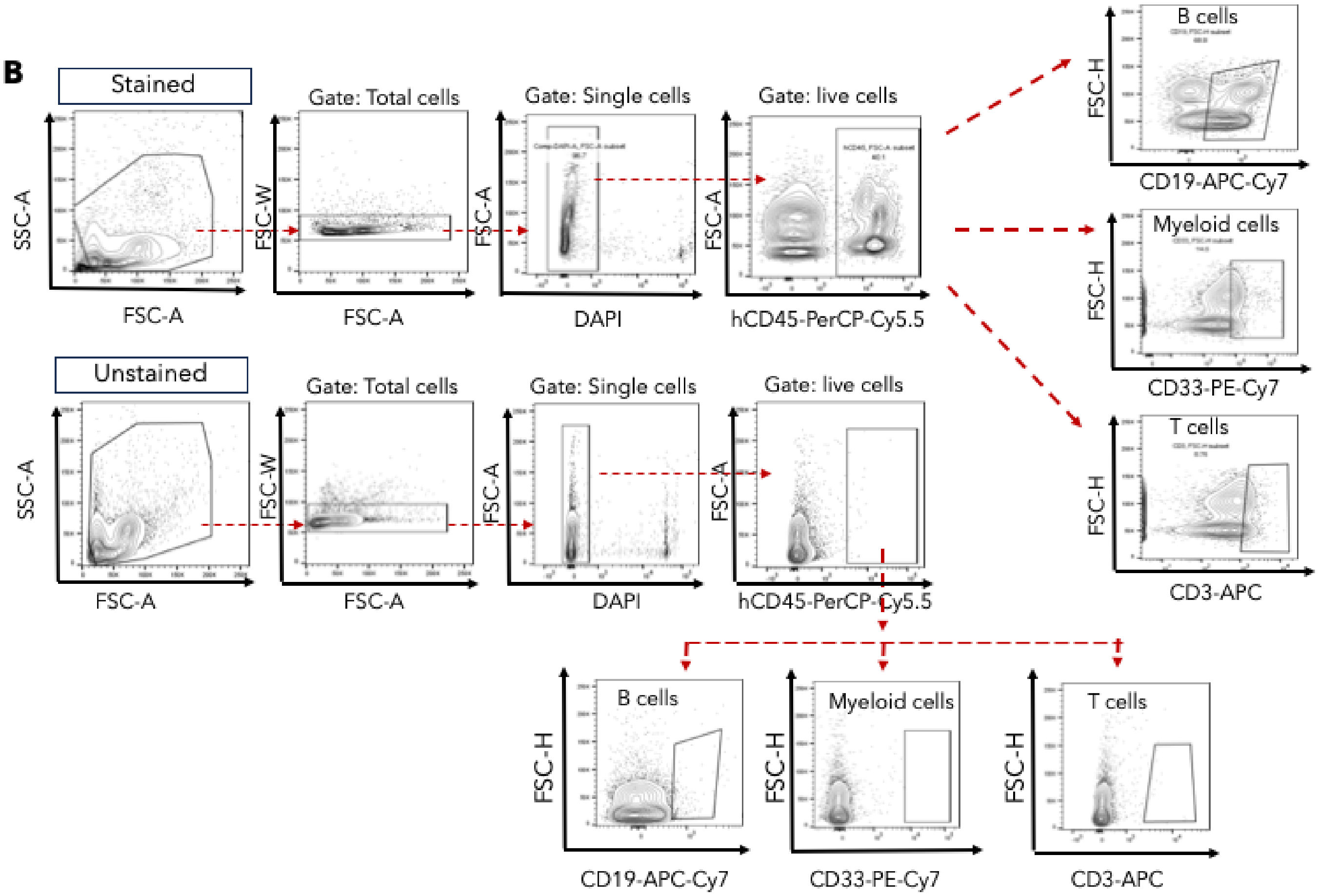

Figure S5: Assessment of human chimerism in xenotransplanted NSG mice. (**A)** Representative gating strategy for the for the analysis of hematopoietic subpopulations in the hematopoietic organs of transplanted mice.

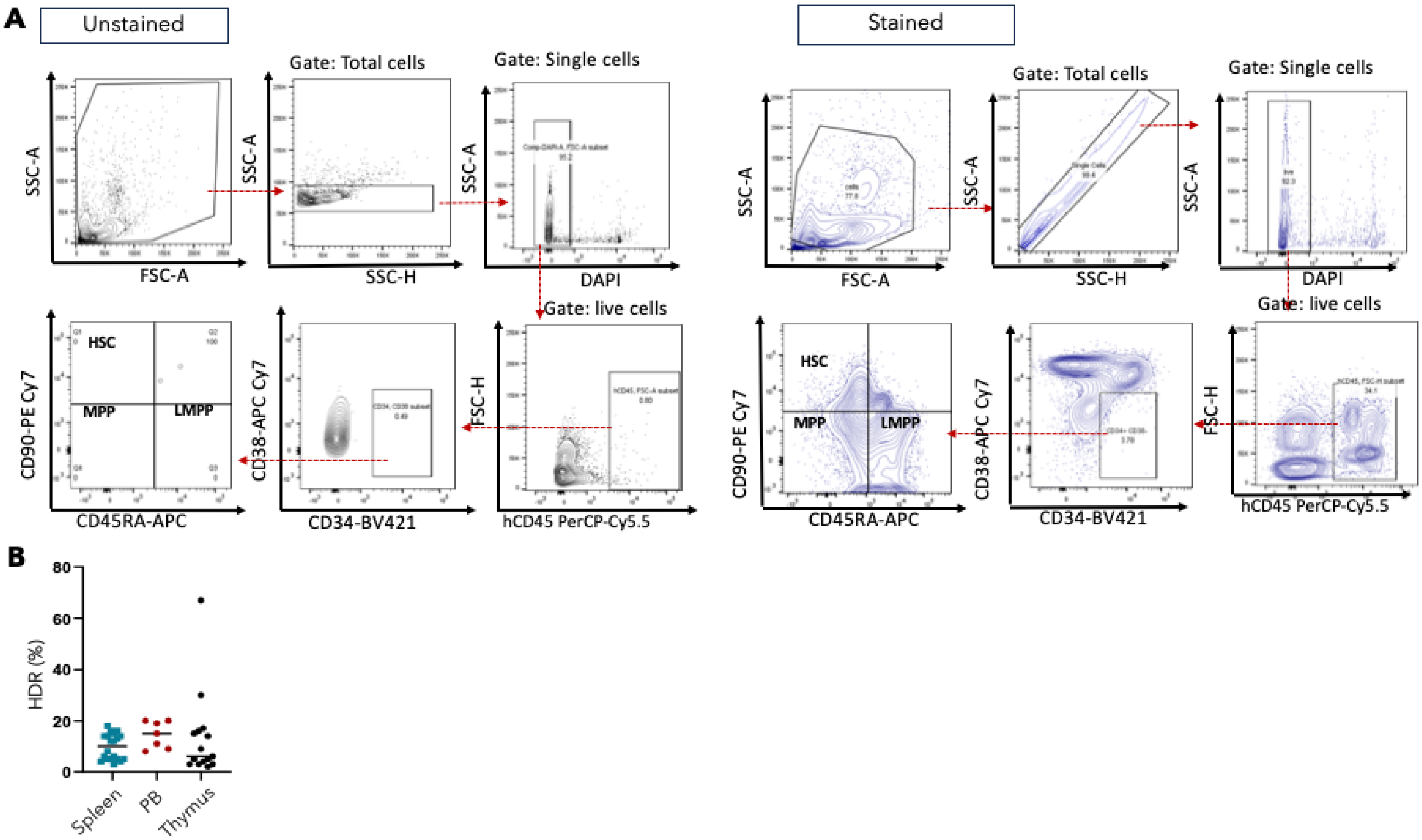

Figure S6: Assessment of human chimerism in xenotransplanted NSG mice. (**A)** Representative gating strategy for the for the analysis of HSPC subpopulations in the bone marrow of transplanted mice. Human cells (hCD45+) were isolated from the BM of mice 14 weeks post injections. hCD45+ population was phenotyped into primitive HSCs (CD34+ CD38- CD90+ CD45RA-), MPPs (CD34+ CD38- CD90- CD45RA-) and CD38+ committed progenitors (CD34+ CD38+). (**B)** Frequency of coWAS cassette integration via HDR in the PB, spleen and thymus of mice transplanted with SU-1 RNP+AAV HSPCs, as detected by ddPCR. Each dot represents a mouse.

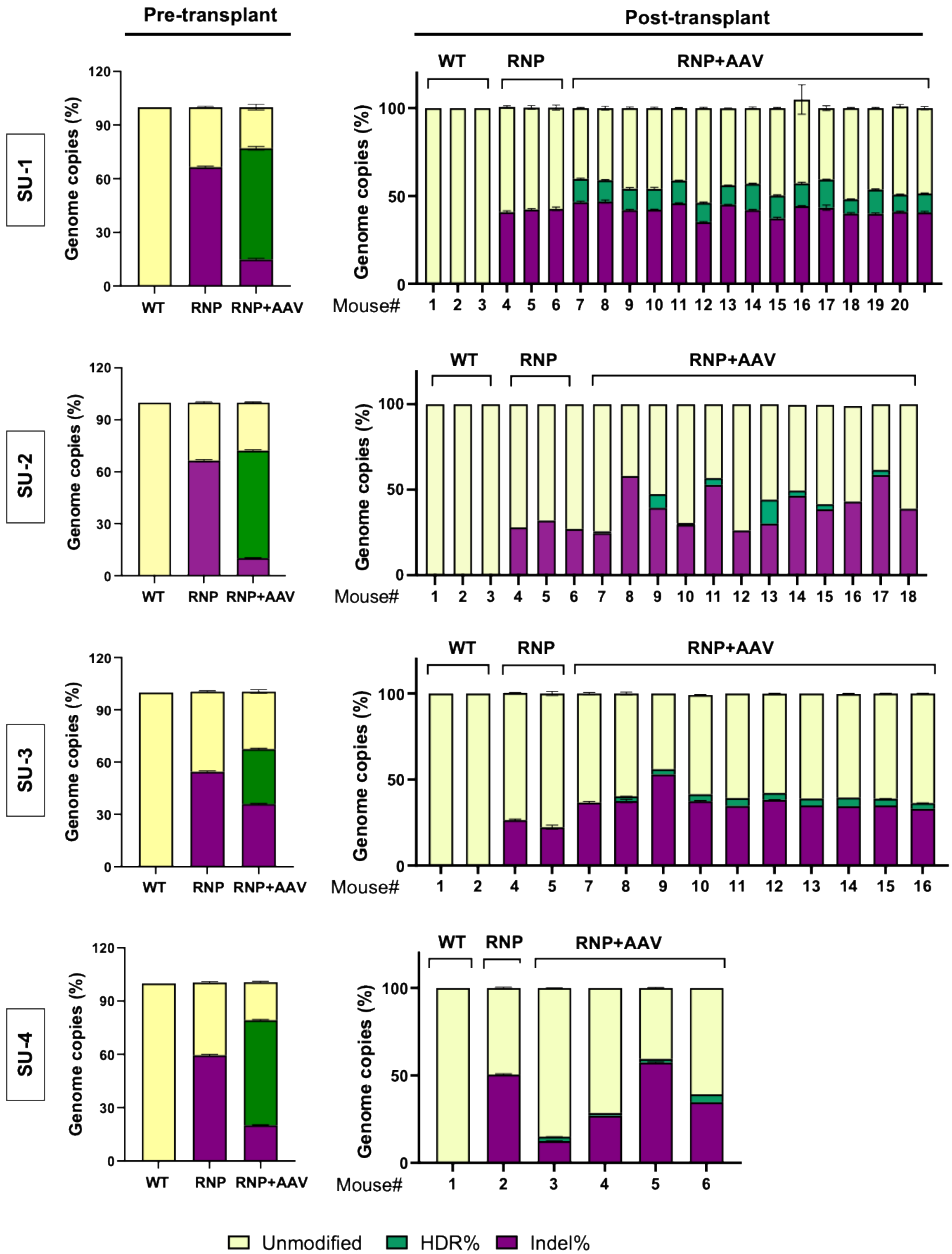

Figure S7: Assessment of gene editing frequency pre and post-transplantation into NSG mice. Fraction of cell genomes with a *WAS* locus unmodified, bearing indels (Indel%) or with coWAS cassette integration (HDR%) in each ex vivo HSPC scale-up product (pre-transplantation) and in CD45+ cells retrieved from the bone marrow of mice transplanted with each of the HSPC product indicated (post-transplantation) as detected by ddPCR. Data are presented as mean ± SD, where n=3 technical ddPCR replicates.

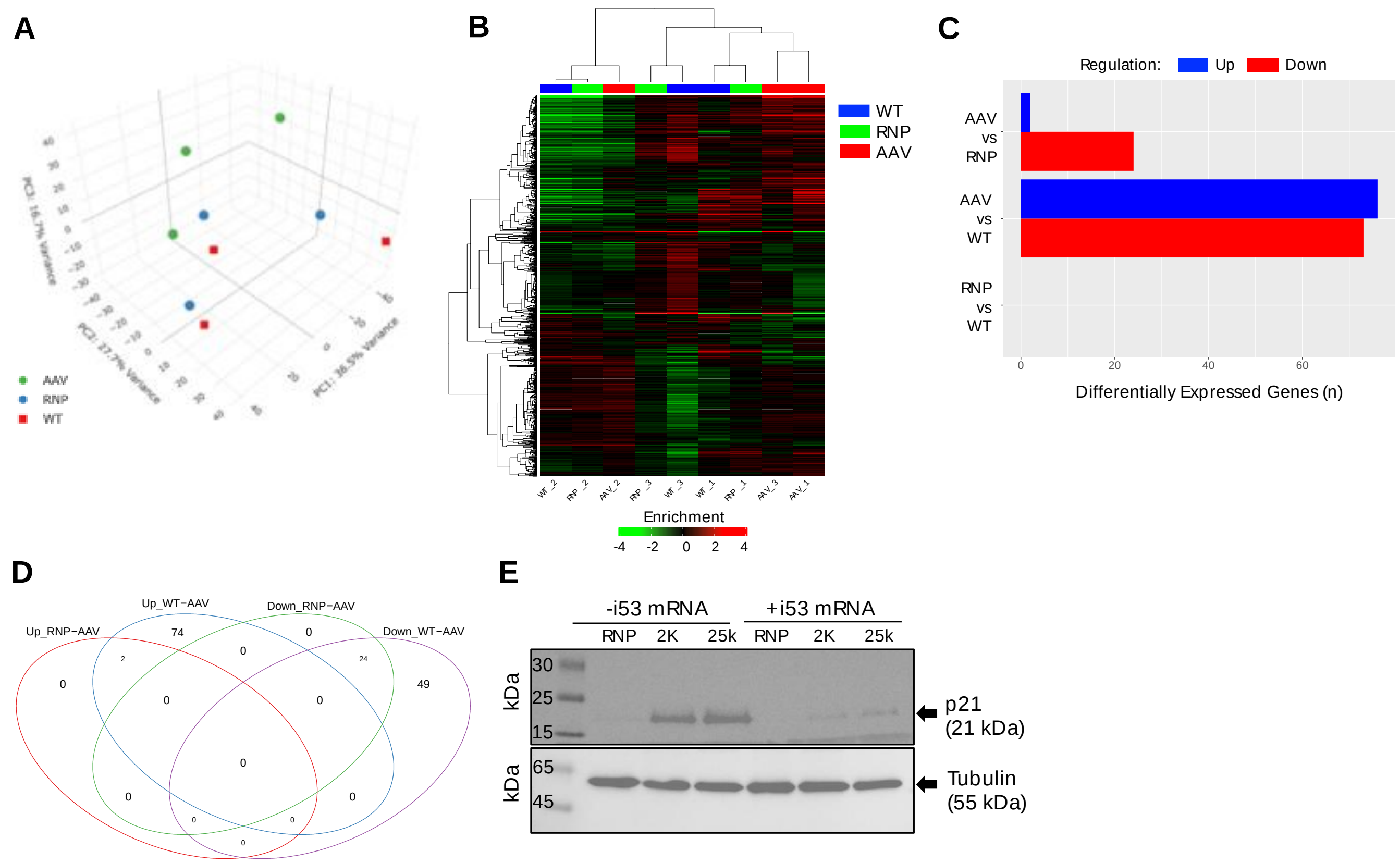

Figure S8: Dampening of p53 signalling improves engraftment of HSPCs that underwent HDR.
(A) Principal Component Analysis conducted on the whole RNA-seq data retrieved from the 3 experimental groups (n=3 biological replicates each). (B) Unsupervised hierarchical clustering of RNA-seq data. (C) Number of genes down- or up-regulated for each pairwise comparison. (D) Venn diagrams displaying the number of differentially expressed genes for each pairwise comparison (RNP vs RNP+AAV or WT vs RNP+AAV) and overlaps among groups. (E) Representative Western blot of p21 expression in HSPCs gene edited using different AAV MOIs and with or without the addition of i53 mRNA. Tubulin was used as a reference for protein quantification.

A

Mouse#	SU1		SU2		SU3		SU4	
	BM smear	Spleen	BM smear	Spleen	BM smear	Spleen	BM smear	Spleen
WT	WNL	EMH	WNL	EMH	WNL	EMH	WNL	EMH
WT	WNL	EMH	WNL	EMH	WNL	EMH	WNL	EMH
WT	WNL	EMH	WNL	EMH	WNL	EMH	WNL	EMH
WT	WNL	EMH	WNL	EMH				
WT	WNL	EMH						
RNP+AAV	WNL	EMH	WNL	EMH	WNL	EMH	WNL	EMH
RNP+AAV	WNL	EMH	WNL	EMH	WNL	EMH	WNL	EMH
RNP+AAV	WNL	EMH	WNL	EMH	WNL	EMH	WNL	EMH
RNP+AAV	WNL	EMH	WNL	EMH	WNL	EMH	WNL	EMH
RNP+AAV	WNL	EMH	WNL	EMH				
RNP+AAV	WNL	EMH	WNL	EMH				
RNP+AAV	WNL	EMH	WNL	EMH				
RNP+AAV	WNL	EMH	WNL	EMH				
RNP+AAV	WNL	EMH	WNL	EMH				
RNP+AAV	WNL	EMH	WNL	EMH				
RNP+AAV	WNL	EMH	WNL	EMH				
RNP+AAV	WNL	EMH	WNL	EMH				
RNP+AAV	WNL	EMH	WNL	EMH				
RNP+AAV	WNL	EMH	WNL	EMH				
RNP+AAV	WNL	EMH	WNL	EMH				
	normal	minimal	mild	moderate	severe			

B

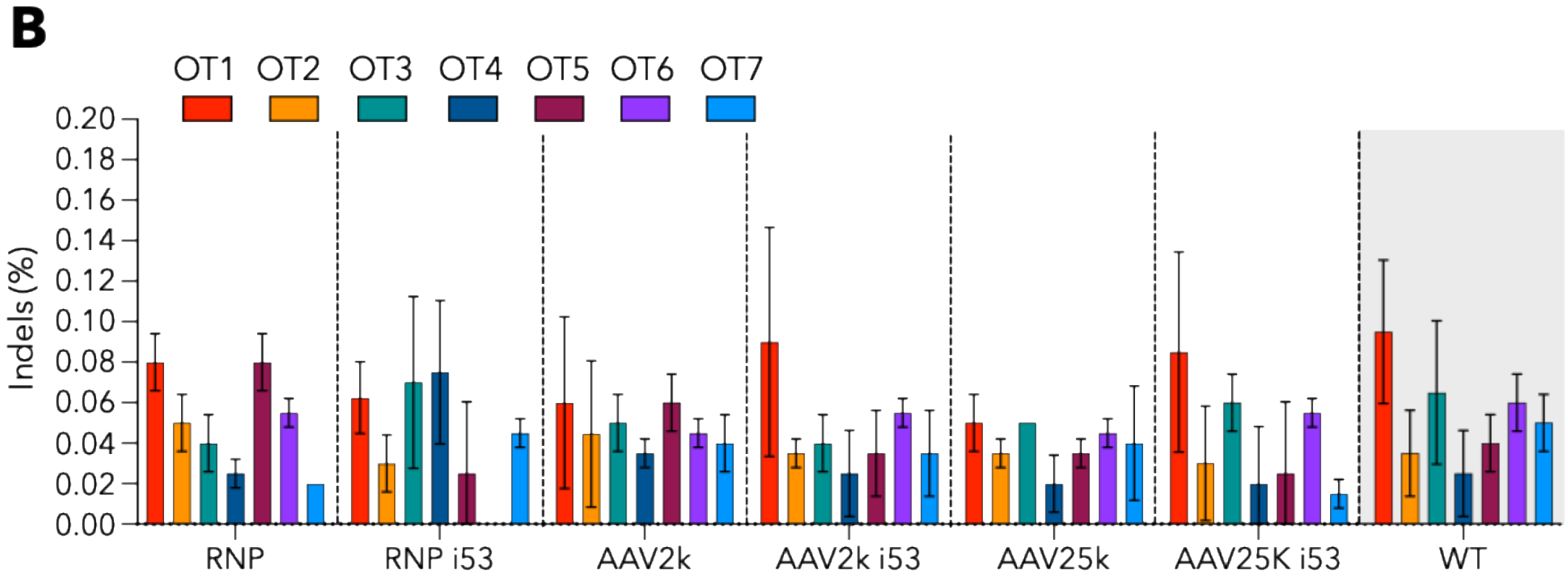

C

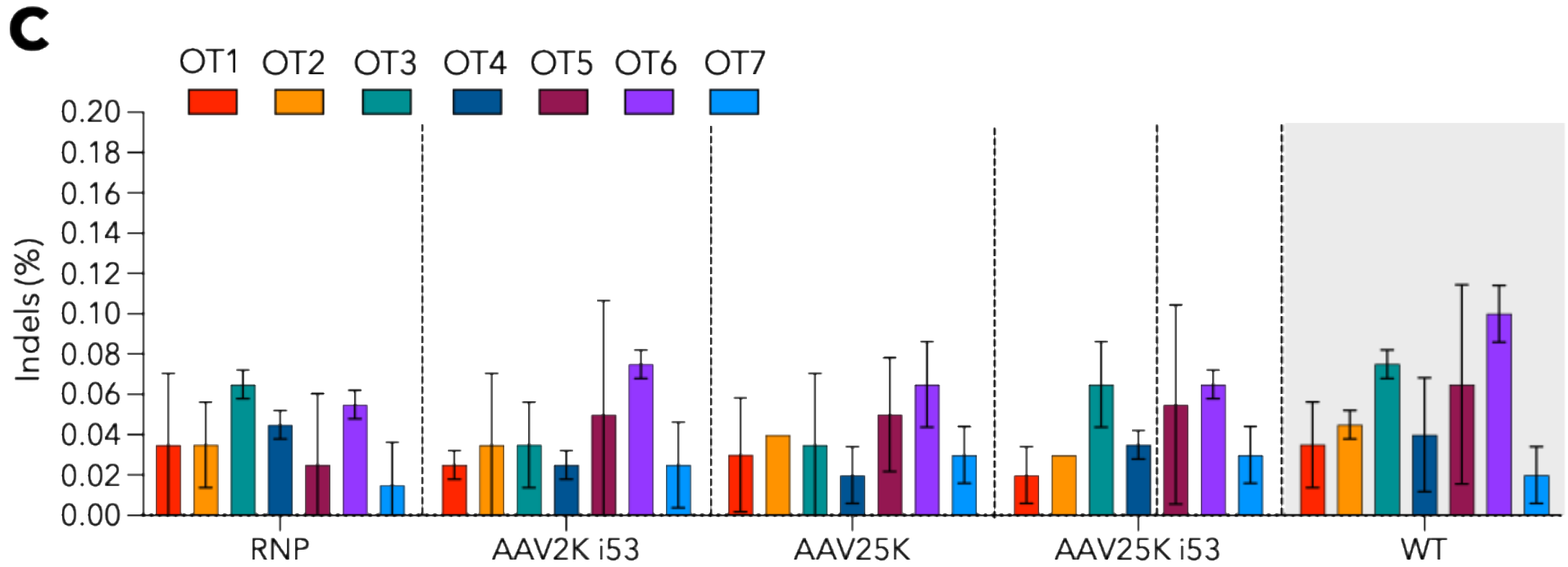

Figure S9. Evaluating the genotoxicity of the gene editing platform in vitro and in vivo. (A) Table summarising histopathological results obtained by analysing BM smears and spleen sections upon H&E staining. BM smears revealed expected morphology and normal levels of precursor cells among the groups. Minimal to mild extra medullary haematopoiesis (EMH) was observed in spleen sections, comparable among the groups.

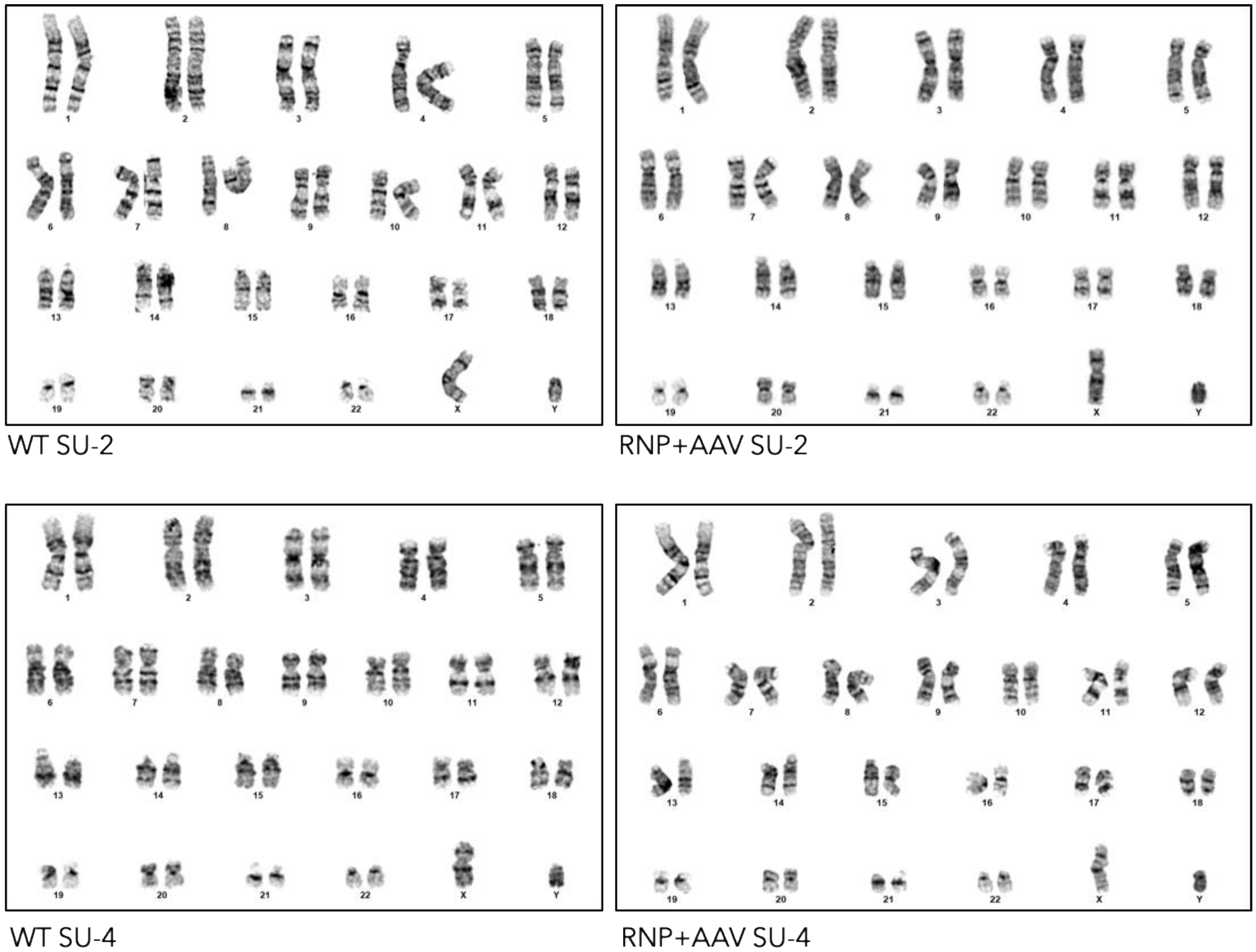

Figure S10. Evaluating the genotoxicity of the gene editing platform in vitro. Karyotypes of edited (RNP+AAV) and unedited (WT) HSPCs from individual scale up experiments.

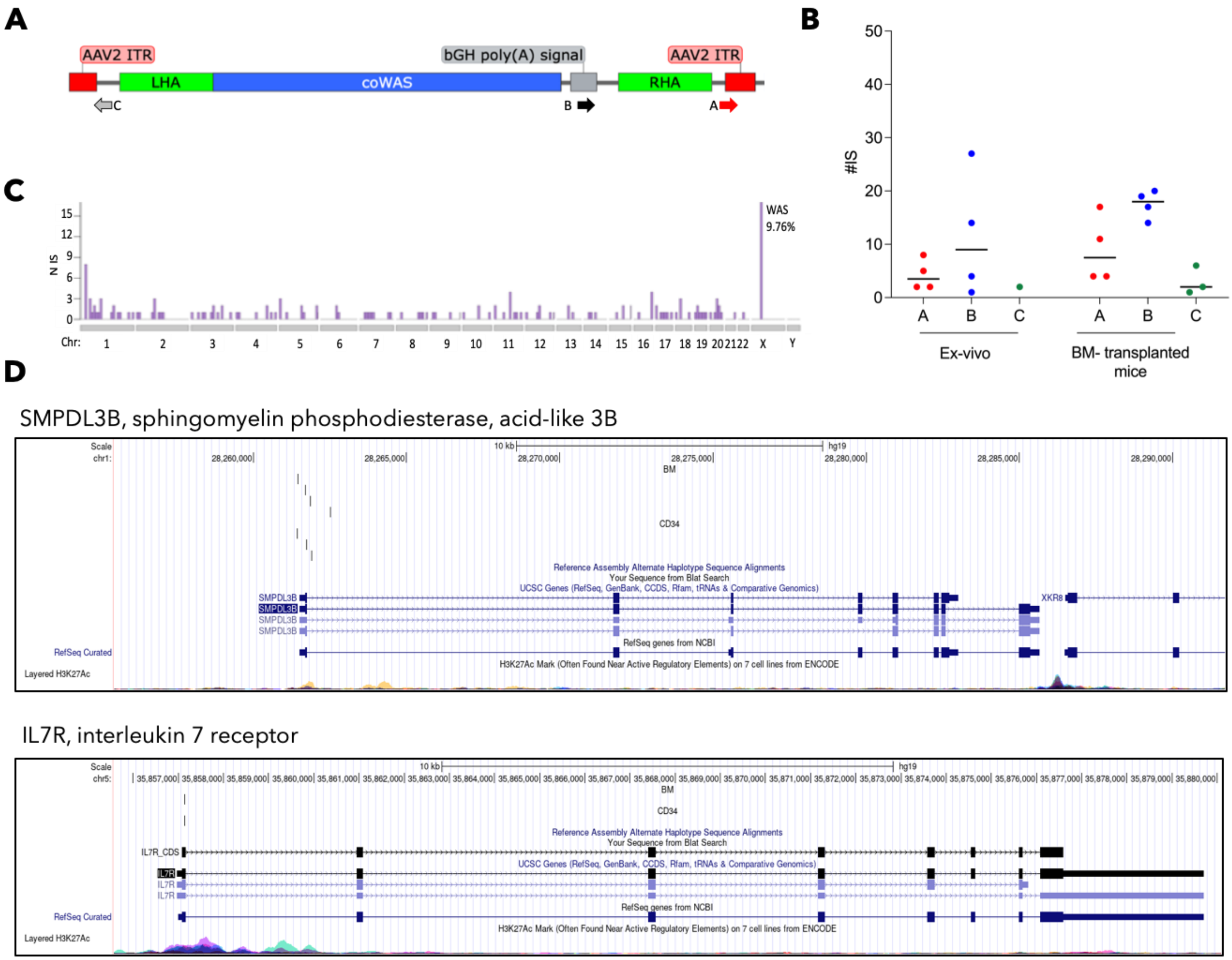

Figure S11. Analysis of AAV donor molecule integration in gene edited HSPCs.
(A) Schematic representation of the strategy used to map AAV integrations involving different features of the AAV donor vector. (B) Number of AAV integration sites (IS) per each sample analysed, ex vivo and in vivo. (C) Distribution of AAV integrations across chromosomes. High IS integration frequency was observed in chromosome X where the *WAS* locus is located. (D) Representative UCSC Genome Browser snapshot of two genes hit by AAV integration retrieved from edited HSPCs (CD34 track) or from CD45+ cells isolated from the BM of transplanted mice (BM track).

Table S1. Genomic sites analysed by amplicon deep-sequencing to assess off-target editing.

Deetction method	Site name	BED Chromosome	Off-Target start	Off-Target end	Strand	Off-Target Sequence	Mismatches
Guide-seq	OT-2	chr1	50322318	50322341	+	GGAAACAG CACCCTGA GAGGAGG	5
	OT-3	chr2	112824051	112824074	-	GCAGCATG AACAATGG GTGGAGG	5
	OT-4	chr2	197121373	197121396	+	ACACAAAG CACCATGA GTGGAGG	2
	OT-5	chr3	2220065	2220088	-	GGAGAAAA CACTCTGA GTGATGG	5
	OT-6	chr6	159803440	159803463	-	GCAGAGGG CAGAGTGA GTGGAGG	5
	OT-7	chrX	124413658	124413681	+	GCATAAAG CACAAGGA GAGGTGA	5
Cosmid+NGS	OT-1	Chr 5	178282798	178282820	+	AAAGGAAG CACCATGA GTGGGGG	3

Table S2. Sequencing results of the translocation detected by CAST-seq in the BM of SU-3 HSPC transplanted mice

chr	start	end	read (n)	hits (n)	read (n) ctrl	hits (n) ctrl	group	annotation	Distance To TSS	Gene	Genes within 100kb	Oncogenes within 100kb
**X**	48674308	48687957	16465	1974	2917	488	OMT	Promoter (<=1kb)	0	WAS	GLOD5; RBM3; WDR13 ; SUV39H1; WAS	WAS
**17**	19697297	19697797	257	34	3	3	NBS	Intron (ENST00000325411.9/146802, intron 13 of 16)	21182	SLC47A2	SLC47A2; ALDH3A1; ALDH3A2 ; ULK2	

Table S3. List and description of AAV integration sites retrieved in this study.

For each IS matrix there are the following columns:
chr: chromosome number
integration_locus: genomic position of the integration sites
GeneID by RefSeq RefSeq Gene Symbol of the closest gene targeted by vector insertion
Gene strand: genomic orientation of the targeted gene
MhOR/ins: Micro-Homology Region (MhOR): Nucleotides overlap between alignments of AAV and target genome (indicated as negative number) / insertion (ins): De novo insertion of nucleotides between AAV and host genome breakpoint (indicated as positive number)
Junction: nucleotide position on the AAV genome at the vector/cellular genome breakpoint
vector_fragments: number of AAV fragments identified within the vector portion of the reads
sample_columns: column name contained the sample ID, the analyzed tissue, the PCR-set from which the IS was identified and eventually the vector dose. Within the column is indicated the number of different genomic fragments identified for each IS

Table S4. Most targeted genes by AAV integration.

ALL IS N= 164			
chr	GeneID	Targeting	%
chrX	WAS	16	9.76
chr1	SMPDL3B	7	4.27
chr1	KDM4A	3	1.83
chr2	DHFRP3	3	1.83
chr10	VSIR	2	1.22
chr12	GRIP1	2	1.22
chr13	PCDH17	2	1.22
chr15	LRRK1	2	1.22
chr18	ZNF521	2	1.22
chr19	MRI1	2	1.22
chr20	CTNNBL1	2	1.22
chr3	FHIT	2	1.22
chr3	MIR548AB	2	1.22
chr5	MIR4456	2	1.22
chr6	EEF1A1	2	1.22
**CD34 N=115**			
chr	GeneID	Targeting	%
chrX	WAS	10	8.7
chr1	SMPDL3B	3	2.61
chr1	KDM4A	3	2.61
chr10	VSIR	2	1.74
chr12	GRIP1	2	1.74
chr2	DHFRP3	2	1.74
chr20	CTNNBL1	2	1.74
chr3	FHIT	2	1.74
chr5	MIR4456	2	1.74
**BM=57**			
chr	GeneID	Targeting	%
chrX	WAS	6	10.71
chr1	SMPDL3B	4	7.14
chr13	PCDH17	2	3.57
chr18	ZNF521	2	3.57
chr6	EEF1A1	2	3.57

Table S5. AAV integration sites abundance in SU samples in vitro (relative to Figure 6G)

SU-1 CD34			SU-2CD34			SU-3 CD34			SU-4 CD34		
GeneID	%	Cell count	GeneID	%	Cell count	GeneID	%	Cell count	GeneID	%	Cell count
IL7R	30.4	38	SHANK2	6.7	6	ATP6V1C2	30.9	17	WAS	8.2	6
PCDH17	10.4	13	WAS	5.6	5	LINC01608	7.3	4	WAS	8.2	6
ZBTB38	8	10	WAS	4.5	4	SMPDL3B	5.4	3	NUP88	6.8	5
SMPDL3B	4	5	LRRK1	3.4	3	TRPM4	5.4	3	LOC100507468	5.5	4
ELMO1	3.2	4	CLTC	3.4	3	OBSCN	3.6	2	C3orf58	4.1	3
KIF17	2.4	3	CTNNBL1	3.4	3	VSIR	3.6	2	LOC101928820	4.1	3
KDM4A	2.4	3	WAS	3.4	3	TUB	3.6	2	KDM4A	2.7	2
ITIH5	2.4	3	RABGGTB	2.2	2	GRIP1	3.6	2	LINC00466	2.7	2
GRIP1	2.4	3	SEC22B	2.2	2	C15orf53	3.6	2	PKN2-AS1	2.7	2
WAS	2.4	3	VSIR	2.2	2	GP2	3.6	2	EVI5	2.7	2

Table S6. AAV integration sites abundance tracking in vivo for SU-1 (relative to Figure 6H)

**SU-1 CD34**			**SU-1 17BM**			**SU-1 21BM**		
GeneID	%	Cell count	GeneID	%	Cell count	GeneID	%	Cell count
IL7R	30.4	38	IL7R	42.6	230	WAS	24.3	51
PCDH17	10.4	13	EEF1A1	34.6	187	IL7R	20	42
ZBTB38	8	10	NCF2	8.2	44	EEF1A1	11.9	25
SMPDL3B	4	5	HFM1	2.4	13	ZBTB38	4.8	10
ELMO1	3.2	4	CLPB	1.7	9	PCDH17	3.3	7
KIF17	2.4	3	ZNF521	1.3	7	DAD1	3.3	7
KDM4A	2.4	3	ZBTB38	1.1	6	ELMO1	3.3	7
ITIH5	2.4	3	EEF1A1	1.1	6	WAS	2.4	5
GRIP1	2.4	3	ELMO1	1.1	6	JPH3	1.9	4
WAS	2.4	3	WAS	0.9	5	MRI1	1.9	4

Table S7. AAV integration sites abundance tracking in vivo for SU-3 (relative to Figure 6I)

SU-3 CD34			SU-3 27BM			SU-3 28BM		
GeneID	%	Cell count	GeneID	%	Cell count	GeneID	%	Cell count
ATP6V1C2	30.91	17	SULT1C2P1	65	13	DDX18	26.67	4
LINC01608	7.27	4	ATP6V1C2	20	4	ECHDC2	20	3
SMPDL3B	5.45	3	WAS	15	3	SMCO4	13.33	2
TRPM4	5.45	3				C2CD4C	13.33	2
OBSCN	3.64	2				ATP6V1C2	13.33	2
VSIR	3.64	2				WAS	13.33	2
TUB	3.64	2						
GRIP1	3.64	2						
C15orf53	3.64	2						
GP2	3.64	2						
