# Supplementary material for: In-depth characterization of stem cell potency and genotoxicity for clinical-scale ex vivo CRISPR-Cas9 gene editing
Source: Mol Ther Adv. 2026 Jul 22;34(3):201816. doi: 10.1016/j.omta.2026.201816 (PMC13476499; doi:10.1016/j.omta.2026.201816)
Supplement: Supplementary file 2 [file mmc3.pdf]

Molecular Therapy
Advances
Original Article

In-depth characterization of stem cell potency and genotoxicity for clinical-scale *ex vivo* CRISPR-Cas9 gene editing

Asma Naseem,[1] Winston Vetharoy,[1] Thomas E. Whittaker,[1] Federica Zinghirino,[1] Antonio Carusillo,[2] Roopkatha Ghosh,[1] Eduardo Gomez-Castaneda,[1] Hashim Ali,[3] Carlo Cipriani,[4,5] Eugenio Montini,[4] Adrian J. Thrasher,[1,6] Giorgia Santilli,[1,6] Daniela Cesana,[4] Giandomenico Turchiano,[1] and Alessia Cavazza[1,2,6]

[1]Infection, Immunity & Inflammation Research and Teaching Department, UCL Great Ormond Street Institute of Child Health, University College London, 20 Guilford Street, London WC1N 1DZ, UK; [2]Center for Genomic Medicine and Rare Diseases, Department of Medical and Surgical Sciences for Children and Adults, University of Modena and Reggio Emilia, Modena, Italy; [3]Division of Infection and Immunity, University College London, London, UK; [4]San Raffaele Telethon Institute for Gene Therapy, IRCCS San Raffaele Hospital, Milan, Italy; [5]Department of Electronics, Information and Bioengineering, Politecnico di Milano, Milan, Italy; [6]NIHR Great Ormond Street Hospital Biomedical Research Centre, 30 Guilford Street, London WC1N 1EH, UK

Translating CRISPR-Cas9-based homology-directed repair (HDR) strategies into clinical application remains a major challenge due to limited standardization, concerns over safety, and efficacy issues. Here, we present a comprehensive and clinically compliant preclinical framework for the *ex vivo* correction of Wiskott-Aldrich syndrome (WAS) using a CRISPR-Cas9-AAV6 platform targeting hematopoietic stem and progenitor cells (HSPCs). In this study, we established a clinical-compatible platform enabling large-scale manufacturing while preserving HSPC viability, stemness, and multilineage functionality. To overcome low HSPC long-term engraftment, we fine-tuned AAV dosing and transiently modulated p53BP-dependent DNA damage response pathway, achieving significantly improved *in vivo* correction and repopulation. Importantly, we implemented a multi-tiered genotoxicity assessment strategy, integrating in silico, genome-wide, and orthogonal assays, revealing a largely favorable safety profile with minimal off-target risks and no evidence of clonal dominance or transformation during the study period. Longitudinal *in vivo* safety monitoring revealed donor-specific rare off-target events and structural variants. This highlights the crucial importance of patient monitoring after transplantation, further emphasized by the identification of a *de novo* chromosomal rearrangement that could be detected exclusively following cell engraftment in mice. This work offers a robust and adaptable roadmap for future HDR-based gene editing platforms, establishing critical benchmarks for efficacy, safety, and regulatory readiness in the development of advanced therapeutic medicinal products.

INTRODUCTION

The discovery of DNA endonuclease enzymes, especially those linked to the clustered regularly interspaced short palindromic repeats (CRISPR) system, has revolutionized genetic correction in hematopoietic stem and progenitor cells (HSPCs). CRISPR-Cas9 targets specific genomic sites using a guide RNA that pairs with DNA. Once it identifies a sequence adjacent to a protospacer adjacent motif (PAM), it introduces a double-strand break (DSB), which can be repaired by the cell's natural DNA repair mechanisms, such as non-homologous end joining (NHEJ) or microhomology-mediated end joining. Alternatively, if a therapeutic donor sequence is provided alongside CRISPR machinery, the cell can use homology-directed repair (HDR) to incorporate the corrective sequence.[1–7] Because many monogenic disorders arise from loss-of-function mutations affecting entire gene loci, insertion of a complete therapeutic coding sequence via HDR offers a universal therapeutic solution while ensuring controlled gene copy number and transcriptional regulation. In this regard, promising preclinical results have been obtained by delivering CRISPR components to HSPCs via electroporation of ribonucleoprotein (RNP) complexes, combined with HDR donor templates delivered through recombinant adeno-associated virus 6 (AAV6) vectors.[1–7]

Despite its potential, HDR-based gene editing still faces significant challenges in achieving durable corrections for blood-related genetic diseases and thus in reaching clinical translation. One major hurdle is that current techniques primarily edit committed progenitors rather than long-term repopulating stem cells while also imposing detrimental effects on cell fitness, reducing their viability, frequency, and regenerative capacity. Many preclinical studies have reported a decline in hematopoietic repopulation capacity, both *in vitro* and following *in vivo* transplantation. This reduction is primarily

Received 1 August 2025; accepted 20 July 2026;
https://doi.org/10.1016/j.omta.2026.201816.

Correspondence: Alessia Cavazza, Infection, Immunity & Inflammation Research and Teaching Department, UCL Great Ormond Street Institute of Child Health, University College London, 20 Guilford Street, London WC1N 1DZ, UK.
E-mail: a.cavazza@ucl.ac.uk

© 2026 The Authors.
Published by Elsevier Inc. on behalf of The American Society of Gene and Cell Therapy.
This is an open access article under the CC BY license (http://creativecommons.org/licenses/by/4.0/).

attributed to the activation of a p53-mediated DNA damage response and innate immune signaling triggered by Cas9 activity and donor template delivery, particularly when using adeno-associated virus (AAV) vectors. These cellular responses drive hematopoietic stem cell differentiation and contribute to the exhaustion of their long-term repopulating potential.[8–11] As such, efforts to improve both the frequency of corrected primitive hematopoietic stem cells (HSCs) and their engraftment in mice upon *in vivo* transplantation have led to significant results when using optimized protocols to transiently manipulate DNA repair pathways, cell cycle, and culture conditions to favor HDR or to dampen detrimental signaling pathways.[12–15] On the other hand, unintended genetic modifications, RNA editing, integration of portions of donor templates, and chromosomal abnormalities at both on- and off-target sites are potential side effects that still pose a major concern for clinical application of gene editing. Numerous technologies have emerged to assess the safety profiles of gene editors using both qualitative and quantitative assays *in vitro* and in living cells.[16] However, the field has yet to establish standardized protocols to evaluate the genotoxic risks associated with different gene-editing techniques. Additionally, the lack of reliable methods to predict the functional consequences of genomic alterations makes it difficult to interpret the vast amount of safety data generated with respect to their clinical relevance. Therefore, there is an urgent need to establish a consensus on the key aspects that should be addressed in preclinical studies of HSPC gene editing platforms to enable their clinical testing and eventual commercialization.[17]

Previously, we showed that HDR-mediated insertion of a correct copy of the *WAS* gene, involved in the occurrence of a rare primary immunodeficiency Wiskott-Aldrich syndrome (WAS) via CRISPR-Cas9 and AAV6 in patient-derived HSPCs led to full correction of all functional hallmarks of the disease *in vitro* and *in vivo*.[6] The platform reached curative levels of gene knock-in, leading to restored physiological expression of the *WAS* gene, functional correction of all hematopoietic lineages, absence of altered HSPC functionality, and controlled gene integration at the *WAS* locus with normal gene copy number. Here, we present a series of preclinical experiments that define and refine our genome editing strategy, bringing it closer to clinical trial readiness. This set of experiments comprehensively investigate the adoption of a good manufacturing practice (GMP)-grade scale up protocol for editing HSPCs while assessing the functionality of manipulated stem cells and the toxicity and safety of the whole procedure by longitudinal analysis *in vitro* and *in vivo*. Overall, our preclinical studies suggest that HDR-mediated editing of HSPCs at the *WAS* locus is feasible and reproducible at clinical scale, with sustained cell viability and repopulating capacity when optimized protocols are implemented. Thorough safety analysis confirmed absence of genotoxic events caused by the gene editing procedure and highlighted the necessity of patient-tailored assessments and post transplantation evaluations of functional relevance of potential off-target editing. Moreover, this study lays the foundation for developing a clear roadmap of essential preclinical tests required to support the clinical translation of HDR-based gene editing platforms in HSPCs for the treatment of a broad spectrum of blood disorders.

RESULTS

Optimization of HSPC gene editing protocols to cGMP-compliant standards

To set up a GMP-compliant HSPC manipulation pipeline, we fine-tuned our previously developed gene editing protocols to match the standards required for a clinical-grade manufacturing process. First, we modified the AAV6 donor vector carrying a codon-optimized WAS cDNA flanked by *WAS* homology arms[6] by switching from ampicillin to kanamycin resistance in the vector backbone and by shortening the homology arms from ∼750 bp to ∼400 bp for improved AAV production and detection of the knocked-in cassette in target cells and tissues (Figure S1A). By comparing both donor vector designs in *in vitro* knock-in experiments in HSPCs, we confirmed consistent HDR efficiency when using the new construct. Then, we replaced research-grade cell culture and gene editing reagents with GMP-grade ones, again showing stable HDR rates when using the new set of reagents (Figure S1B). Optimization of Cas9:gRNA stoichiometry to reduce overall manufacturing costs as well as to calibrate gRNA complexation with Cas9, resulted in 1:1.65 being the most cost-effective and efficient Cas9:gRNA ratio in our setting, in terms of knock-in efficiency and cell viability (Figure S1C). Lastly, we tested the use of the GMP-compatible MaxCyte GTx electroporator by comparing different electroporation programs to previously used parameters in the Neon NTx electroporation system. By employing the program HSC3 in the MaxCyte GTx machine, we achieved highly consistent HDR rates in HSPCs, paralleled by superior cell viability (Figures S1D and S1E).

Having established a reliable GMP-grade platform to edit HSPCs, we then moved forward to scale up HSPC manipulation to clinical need using the pipeline shown in Figure 1A. CD34+ HSPCs were isolated from 8 batches of granulocyte colony-stimulating factor (G-CSF) mobilized peripheral blood (PB) from healthy donors either manually or using the automated CliniMACS Prodigy system, with successful and reproducible cell purification (Figures 1B and S2). Once purified, HSPCs were pre-stimulated for 2 days in a defined medium supplemented with cytokines, followed by Cas9:gRNA RNP electroporation and transduction with the AAV6 coWAS donor. A portion of edited cells, alongside controls, was then harvested at day 4 for quality control (QC) testing, such as viability, editing efficiency (Indel/HDR) as well as *in vitro* genotoxicity analysis. In parallel, the majority of cells were instead cryopreserved, followed by thawing, QC analysis, and transplantation into animals, to mimic a potential clinical process.

Targeted integration analysis by digital droplet PCR (ddPCR) confirmed insertion of the coWAS cassette in 61% of $CD34^+$ HSPCs when editing an average of 115×10^6 cells, and in 67% of CD34+ HSPCs when using a medium-scale setup involving 5×10^6 cells per experimental replicate. (Figure 1C). HDR rates were stable throughout the *in vitro* culture up to 14 days after editing and in

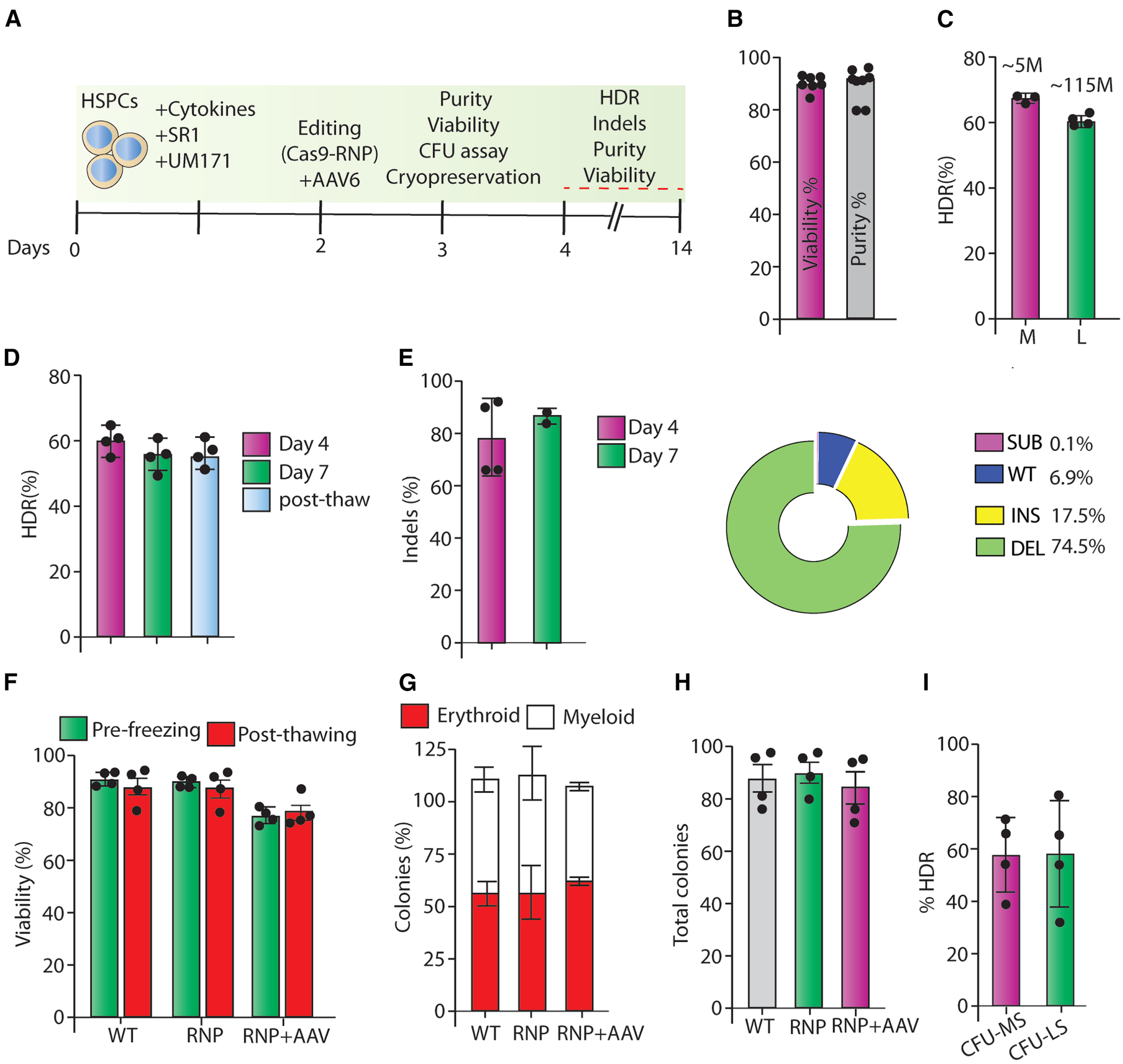

Figure 1. Feasibility of large-scale HSPC gene editing under cGMP-compliant conditions

(A) Schematic overview of the experimental workflow and timeline. (B) Viability and purity (CD34$^+$ cell content) of hematopoietic stem and progenitor cells (HSPCs) isolated from leukapheresis derived from healthy donors (*n* = 7 biological replicates), assessed by flow cytometry. (C) Knockin efficiency of the WAS cDNA cassette in HSPCs from medium-scale (*n* = 4 biological replicates) and large-scale (*n* = 4 biological replicates) experiments, determined by droplet digital PCR (ddPCR). (D) Knockin efficiency in large-scale edited HSPCs as assessed by ddPCR at day 4 and day 7 post editing and after cryopreservation. (E) Bar graph showing insertion/deletion (InDel) frequencies in genomic DNA from RNP-edited HSPCs at day 4 and day 14 post-editing, determined by next-generation sequencing (NGS). The pie chart on the right displays the distribution of predominant mutations identified by NGS. (F) Viability of manipulated (RNP and RNP+AAV) and unmanipulated (WT) HSPCs, evaluated before and after cryopreservation, as assessed via DAPI staining and flow cytometry (*n* = 4 biological replicates). (G) Bar graphs showing the percentage of myeloid (white) and erythroid (red) colonies formed in semi-solid cultures by wild-type (WT), RNP-edited, and RNP+AAV-edited/transduced HSPCs from healthy donors (*n* = 4 biological replicates). (H) Total number of colonies generated in solid cultures as described in (G). (I) Frequency of WAS cassette knockin assessed in colonies generated in semisolid cultures by RNP+AAV HSPCs edited at large (LS) and medium (MS) scale (*n* = 4 biological replicates). The fraction of colonies harboring monoallelic knockin in *WAS* on the X chromosome is presented. Because HSPCs are from male donors, only one WAS copy is amenable to editing, hence colonies can be either edited or not edited.
Data are presented as mean ± SD.

the cryopreserved product upon defrosting and infusion into animals (Figure 1D). Indel rates in electroporated samples (RNP) reached up to 95% (range 67%–95%) and again remained stable throughout the *in vitro* culture (Figure 1E). This underscores absence of toxicity or negative selection of large-scale manipulated cells *in vitro*, further confirmed by >75% of cell viability detected both before and after cryopreservation of the edited cell product, with negligible differences compared to unmanipulated or RNP controls also in terms of cell recovery after thawing (Figures 1F and S3). Importantly, unmanipulated (wild-type, WT) and manipulated

(RNP and RNP+AAV) HSPCs showed comparable clonogenic potential in solid culture, with no significant differences in the overall quantity of colony-forming units (CFU) and no evidence of lineage skewing among the experimental groups (Figures 1G and 1H). Assessment of HDR frequency in randomly picked single CFU colonies showed an average rate of 59% of coWAS targeted integration in HSPCs edited at both large and medium scale (Figure 1I), suggesting efficient editing of cells with repopulating capacity. Overall, these results show that our developed GMP-compliant protocol for clinical scale manufacturing of gene edited HSPCs met all the required specifications for cell product potency (>50% gene correction), cell purity (>70% CD34+), cell viability (>70%), and colony forming capacity in solid cultures (>50 colonies every 500-plated cells).

Robust engraftment of HSPCs manufactured at large scale

To test the ability of the manufactured product to support hematopoietic engraftment and reconstitution in primary recipients, a total of 215 million large-scale edited and cryopreserved HSPCs from 4 different healthy donors (SU-1 to SU-4) were injected intravenously (i.v.) into sub-lethally irradiated non-obese diabetic-scid-gamma (NSG) mice, alongside WT and RNP controls (as detailed in Figures 2A and 2B and materials and methods). Evaluation of engraftment potential and hematopoietic reconstitution, together with HDR and indel frequencies within the CD45+ human cell compartment, was carried out 14 weeks after transplantation. Overall, *ex vivo* cultured and/or edited HSPCs showed robust engraftment capacity, with a median human chimerism in the bone marrow (BM) of around 36% (range 3%–68%), 33% (range 8%–61%), and 30% (range 3%–71%) for WT, RNP, and RNP-AAV, respectively (Figures 2C and S4). The human graft in the BM was multilineage and consisted of mostly CD19+ B cells and CD33+ myeloid cells, as usually seen in NSG mice (Figures 2D and S5). We also observed comparable chimerism in the human CD34+ HSPC, multipotent progenitor (MPP) and HSC compartments among experimental groups (Figures 2E andS6A). While the percentage of engraftment in the BM for the large-scale manufactured products was slightly, although non-significantly, decreased compared to unmanipulated cells, we observed comparable engraftment rates in the periphery (Figures 2F, 2G, and S4). DdPCR analysis on sorted human CD45+ cells from the BM of transplanted mice revealed an average of 39.4% of indels in RNP and 10.1% of HDR-mediated coWAS knock-in in RNP+AAV samples, respectively with a similar trend being also observed in CD45+ cells collected from spleen, PB, and thymus, indicating engraftment of edited HSPCs and their correct differentiation in all the hematopoietic organs (Figures 2H, S6, and S7).

The decrease in the engraftment frequency of HSPCs corrected via HDR over the course of long-term transplantation studies in mice and in patients is a well-known phenomenon, largely ascribed to poor stem cell editing and procedure-related cell toxicity. We therefore sought to investigate the impact of our large-scale gene editing protocol on HSPC gene expression profile by comparing the transcriptome of unmanipulated, electroporated-only (RNP) and fully gene-edited (RNP+AAV) HSPCs collected 2 days after editing, the usual time point for product infusion into patients when treated via gene therapy. Principal component analysis (PCA) highlighted distinct expression profiles imputable mainly to the different donor sources (PC1 and PC2), suggesting minimal influence on the overall transcriptome by the gene editing procedure; only 16% of the variance was indeed attributed to cell manipulation (PC3) and only in the presence of AAV transduction (Figure S8A). This is reflected by the low number of differentially expressed genes (DEGs) retrieved, with 149 and 26 DEGs in AAV+RNP when compared to WT and RNP, respectively (false discovery rate, FDR<0.05; Fc > 2) (Figure 3A). No differential gene expression was observed when comparing WT versus RNP condition, underscoring the lack of toxicity caused by the electroporation procedure optimized in this study (Figures 3B, S8B, and S8C). Since all DEGs emerging from the AAV+RNP versus RNP comparison were shared with the AAV+RNP versus WT comparison (Figure S8D), we focused on the latter and performed a functional enrichment analysis to investigate the presence of pathways and biological processes related to the transcriptome change after HDR-mediated gene editing. Genes differentially expressed in the RNP+AAV condition belong to very interconnected categories in the MSigDB database.,[18] including cell cycle, apoptosis, cell stress, interleukin signaling, DNA repair, and TP53 signaling (Figure 3B). Interestingly, 14/72 genes upregulated in the RNP+AAV condition were direct transcriptional targets of TP53, underscoring the presence of a stress-related HSPC response to the use of AAV donors for HDR, as previously observed.[11,19]

In light of these evidences, and with the aim to increase the frequency of engraftment of HSPCs with a correct coWAS knock-in in the BM of recipient mice, we modified the manufacturing protocol to include the use of an inhibitor of 53BP1, whose inhibition promotes HDR over NHEJ following DSBs and reduces gene editing-mediated cell death and toxicity,[12,14,20] and optimized AAV doses via a response surface methodology approach,[21] to achieve efficient knock-in frequencies while preserving cell fitness. HSPCs from different healthy donors were electroporated with RNPs and transduced with the AAV donor vector under three conditions: at a multiplicity of infection (MOI) of 25,000 with or without i53 mRNA (referred to as AAV 25K and AAV 25K i53, respectively), and at a MOI of 2,000 with i53 mRNA (AAV 2K i53). All manipulations were performed within the framework of our optimized large-scale manufacturing protocol. Culture and manipulation of HSPCs with the presence of i53 mRNA greatly reduced p21 expression irrespective of the AAV doses used (Figure S8E). DdPCR revealed correct knock-in of the coWAS cassette in the *WAS* locus, with an HDR rate of 26.8% in the AAV 25K condition, which decreased to 18.5% when scaling down AAV dose by 10-fold (Figure 3C). The addition of i53 to the culture medium resulted in a significant increase in knock-in efficiency with both AAV doses tested, confirming the role of i53 in the regulation of DNA repair pathways favoring HDR.[12,14] HSPCs placed in solid cultures gave rise to a significantly increased numbers of colonies when i53 was supplemented to the culture media, and the effect was further and significantly enhanced when combined with reduced

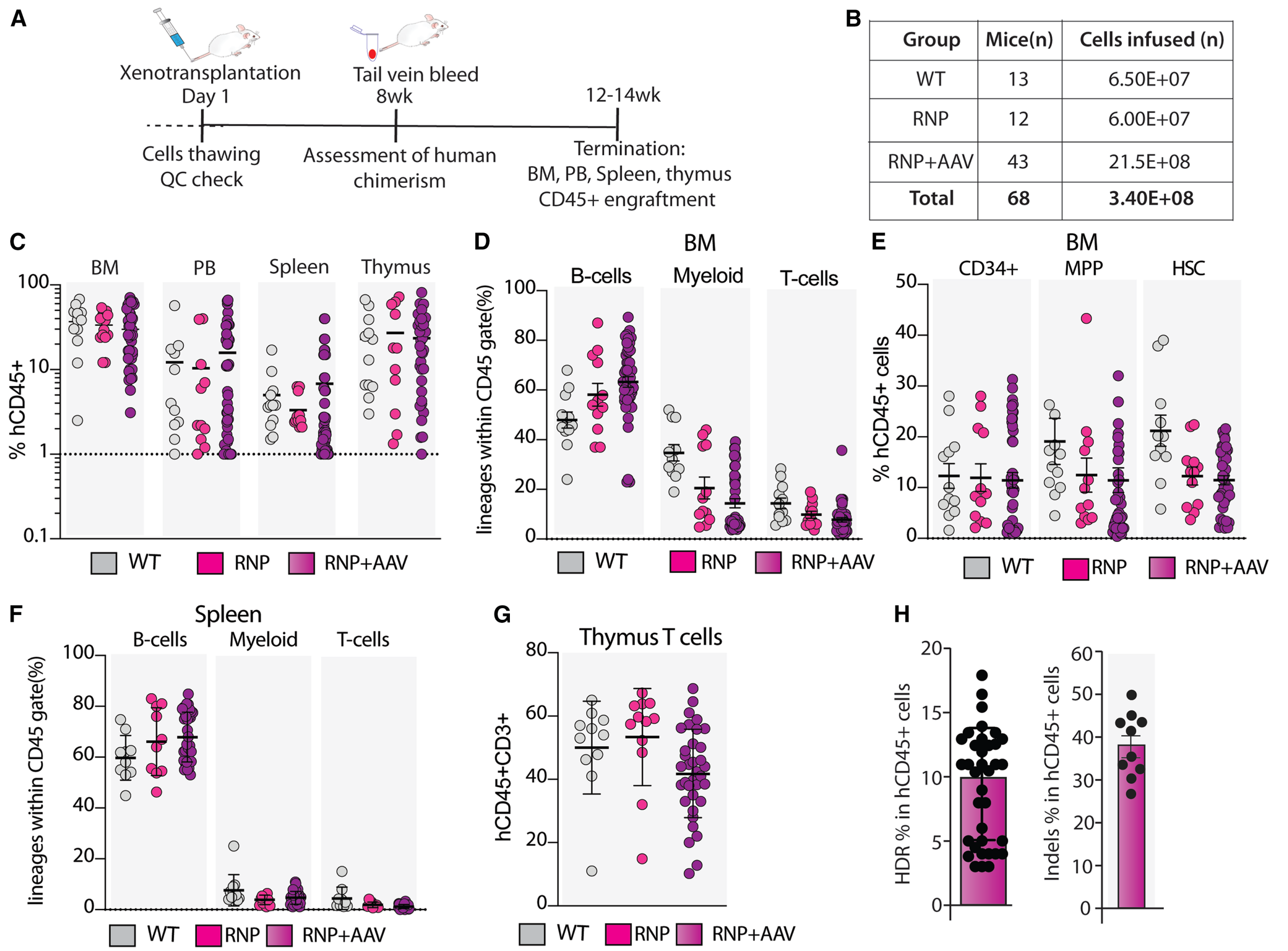

Group	Mice(n)	Cells infused (n)
WT	13	6.50E+07
RNP	12	6.00E+07
RNP+AAV	43	21.5E+08
**Total**	**68**	**3.40E+08**

Figure 2. Effective engraftment and multilineage differentiation of large-scale gene-edited HSPCs *in vivo*

(A) Schematic representation of the *in vivo* experimental design using the NSG mouse model. (B) Summary table indicating the total number of HSPCs injected and the number of recipient animals per experimental group. (C) Human chimerism levels, expressed as percentages of CD45+ cells, in bone marrow (BM), spleen, peripheral blood (PB), and thymus of transplanted NSG mice, assessed 14 weeks post-transplantation. (D) Proportional distribution of human hematopoietic lineages (B cells, T cells, and myeloid cells) within the human (hCD45$^+$) cell compartment in the BM. (E) Distribution of human stem and progenitor subsets—multipotent progenitors (MPP), hematopoietic stem cells (HSCs), and CD34$^+$ cells—within the BM human cell population in transplanted mice. (F) Lineage distribution of human hematopoietic cells in the spleen of transplanted mice, as described in (D). (G) Frequency of CD3$^+$ T cells within the hCD45$^+$ population in the thymi of transplanted mice across all experimental groups. (H) Quantification of WAS cassette integration (HDR) and InDel formation in human CD45+ cells isolated from the BM of mice transplanted with RNP+AAV HSPCs, measured by ddPCR and Sanger sequencing, respectively. Each dot represents data retrieved from one mouse in figure C–H.
Data are presented as mean ± SD, except for panels C–E where mean ± SD was used.

AAV doses, with almost a 4-fold increase in colony numbers compared to the AAV 25k condition (Figure 3D). Two days post editing, cells were transplanted into NSG mice alongside unmanipulated controls and engraftment was assessed 12–14 weeks later. We observed an average of 45% (range 42%–47%) engraftment in the BM and 37% (range 32%–43%) in peripheral hematopoietic organs in all experimental conditions where AAV was employed, with a small but significant decrease compared to unmanipulated and RNP controls, especially in the BM and in the spleen (Figure 3E). DdPCR analysis on human CD45+ cells sorted from the BM, PB, and spleen of transplanted mice showed a visible trend toward increased engraftment of RNP+AAV manipulated cells when i53 mRNA was included in the culture media, which was even more pronounced when combined with lower AAV doses (Figure 3F). If compared to pre-transplant HDR rates, AAV 2K i53 HSPCs displayed the lowest drop in HDR post-transplant in all hematopoietic organs analyzed (average of 7.6, 5, and 3.8-fold decrease in the BM, spleen, and PB, respectively) followed by AAV 25K i53 (average of 18, 9.1, and 5.9-fold decrease in the BM, spleen, and PB, respectively) and AAV 25K (average of 31.4, 22, and 44-fold decrease in in the BM, spleen, and PB, respectively) (Figure 3G), further confirming the detrimental effect that AAV donors at high doses exert on edited

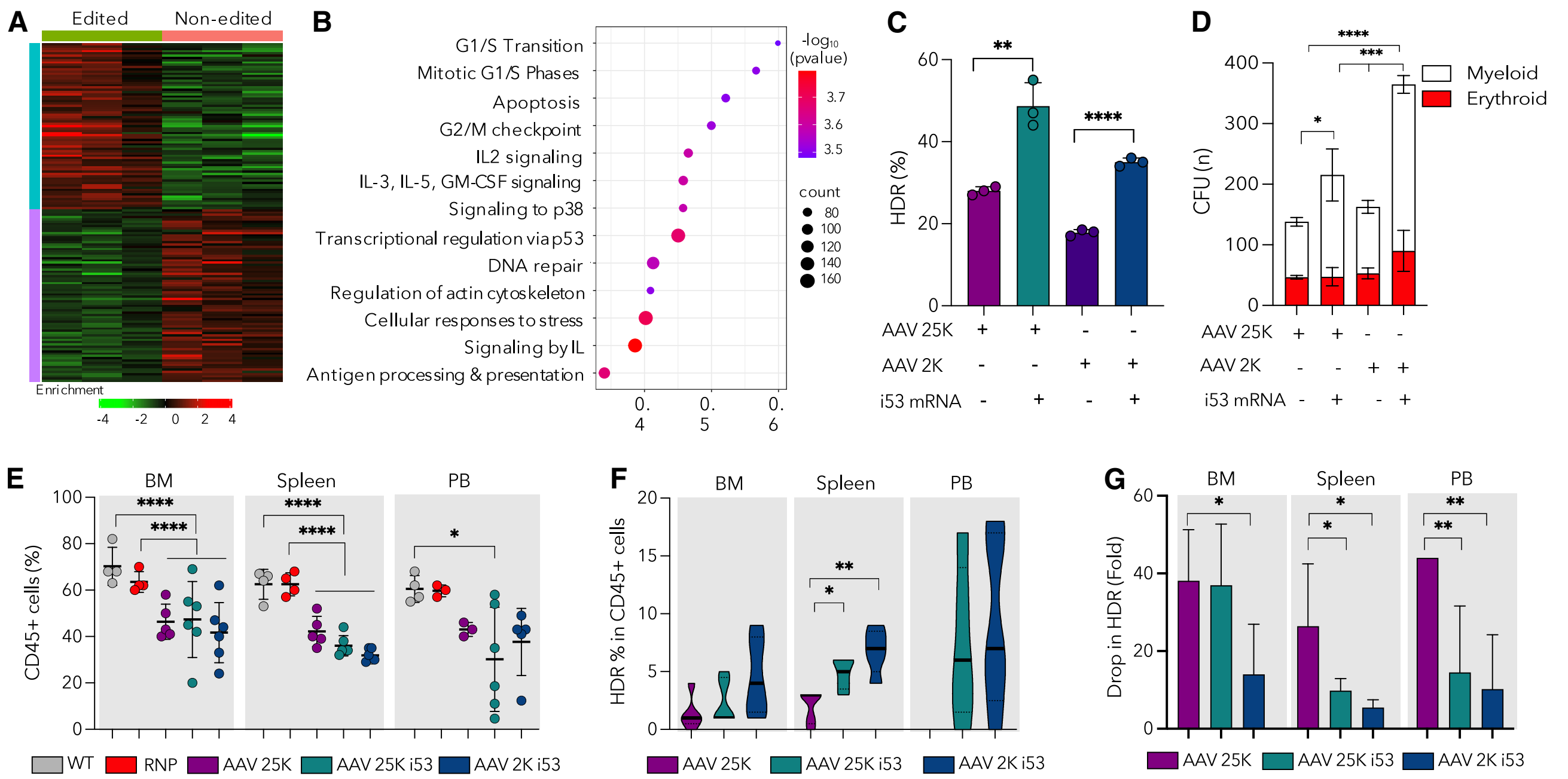

Figure 3. Dampening of p53 signaling improves engraftment of HSPCs that underwent HDR

(A) Representative hierarchical clustering map of differentially expressed genes when comparing unedited (WT) and edited (RNP+AAV) HSPCs transcriptomes (n = 3 biological replicates). The blue bar indicates the cluster of genes upregulated in edited HSPCs, while the purple bar indicates the cluster of genes downregulated in edited HSPCs. (B) Pathway enrichment bubble plot, representing the pathways most enriched in differentially expressed genes when comparing unedited (WT) and edited (RNP+AAV) HSPCs transcriptomes. p values are represented by bubble color, gene counts are represented by bubble size. (C) Frequency of HDR observed in RNP+AAV HSPCs edited using different AAV MOIs with or without the addition of i53 mRNA. (D) Bar graphs showing the percentage of myeloid (white) and erythroid (red) colonies formed in semi-solid cultures by RNP+AAV HSPCs edited using different conditions as described in (C). ****$p < 0.0001$, ***$p < 0.001$, *$p < 0.05$; two-way ANOVA with Tukey multiple comparison test. (E) Human chimerism levels, expressed as percentages of CD45+ cells, in bone marrow (BM), spleen and peripheral blood (PB) of transplanted NSG mice, assessed 12–14 weeks post-transplantation. Each dot represent data retrieved from one mouse (n = 4–6 mice per experimental group; ****$p < 0.0001$, *$p < 0.05$; 1-way ANOVA with Tukey multiple comparison test). (F) Quantification of WAS cassette integration (HDR) in human CD45+ cells isolated from the BM, spleen and PB of mice transplanted with RNP+AAV HSPCs edited using different conditions, measured by ddPCR. Each dot represent data retrieved from one mouse (n = 4–6 mice per experimental group; **$p < 0.01$, *$p < 0.05$; two-way ANOVA with Tukey multiple comparison test). (G) Bar plot representing the drop in HDR frequency post-transplantation for each experimental condition analyzed. The drop was calculated as fold decrease in HDR frequency observed for each experimental mice (n = 4–6 mice per experimental group; **$p < 0.01$, *$p < 0.05$; two-way ANOVA with Tukey multiple comparison test) at 12–14 weeks post-transplantation in CD45+ cells in the BM, spleen and PB, compared to pre-transplant HDR rates observed in HSPCs for each experimental condition (C).
Data are presented as mean ± SD.

HSPCs and the benefit of fine-tuning multiple manipulation conditions to achieve better therapeutic outcomes.

No detectable *in vivo* toxicity after transplantation of HSPCs produced at clinical scale

To assess the potential for *in vivo* toxicity and tumorigenesis as a result of transplantation of large-scale edited HSPCs, we closely monitored transplanted mice for the development of adverse clinical signs over the 14 weeks following HSPCs infusion (Figure 4A). From a toxicology point of view, no mice experienced weight loss after BM conditioning and HSPCs transplantation and overall, all mice gained weight regularly and survived until the end of the experiment (Figure 4B). PB was collected at termination for hematological analysis, showing no significant difference in the complete blood count and leukocyte differential among experimental groups, suggesting absence of major toxic effects (Figures 4C and 4D). Histopathological analysis of BM smears and spleen sections of 55 transplanted mice carried out in certified good laboratory practice (GLP) conditions confirmed presence of all precursor/progenitor lineages as well as absence of any gross lesions or adverse effects attributable to cellular infusion. Furthermore, no evidence of morphologically disrupted hematopoiesis was reported in all the mice samples analyzed, confirming absence of tumorigenesis (Figures 4E, 4F, and S9A).

Extensive genotoxicity assessment demonstrates safety of the gene editing platform

A key objective of this preclinical study is to assess the safety of the gene editing platform by ensuring efficient on-target genetic correction while minimizing off-target effects. Indeed, one of the primary risks associated with CRISPR technology is the potential for unintended genetic modifications and rearrangements at both on- and off-target sites, which could introduce mutations that may lead to oncogenic transformation, posing a significant concern for clinical

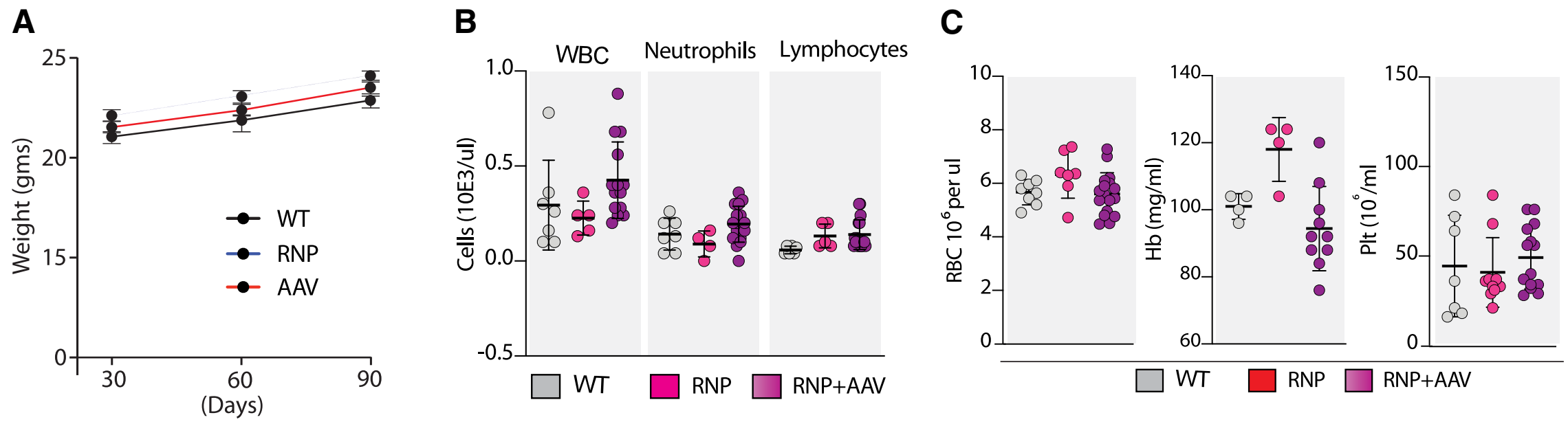

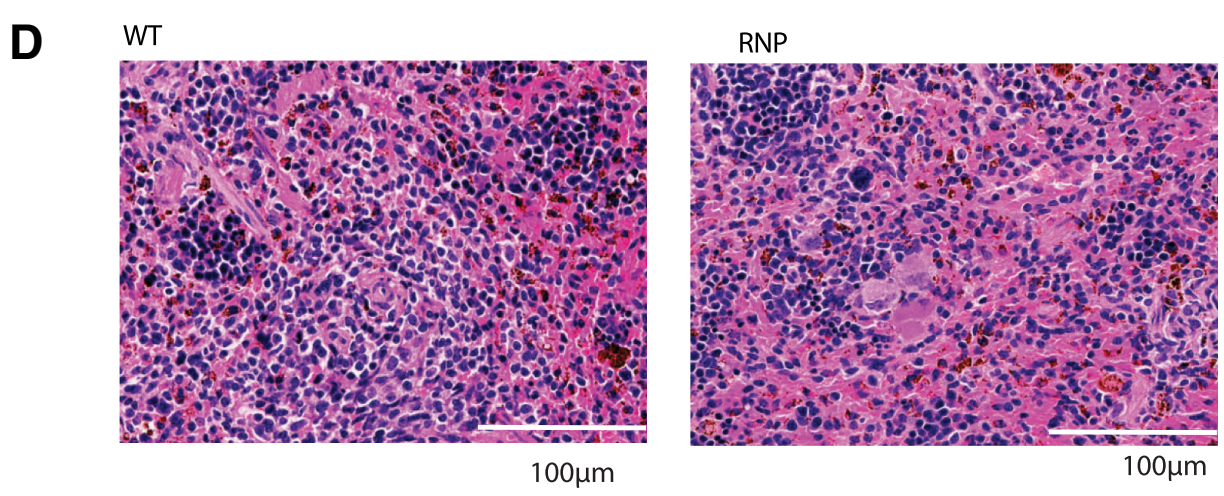

E

Group	Mice(n)	BM	Spleen
WT	13	WNL	Mild EMH
RNP	3	WNL	Mild EMH
RNP+AAV	39	WNL	Mild EMH
**Total**	**55**		

WNL: Within normal limits
EMH: Extramedullary Hematopoiesis

Figure 4. No evidence of *in vivo* toxicity upon transplantation of HSPCs manufactured at clinical scale

(A) Body weight measurements of NSG mice from transplantation through to the experimental endpoint. (B and C) Hematological parameters (white blood cells, red blood cells, and platelets) measured in PB of xenotransplanted NSG mice at 14 weeks post-injection. Each dot represents data retrieved from one mouse. (D) Representative images from histopathological analysis of spleen sections stained with hematoxylin and eosin (H&E), captured at 20× magnification. Images shown were taken from mice transplanted with unmanipulated (WT) or edited (RNP+AAV) HSPCs. (E) Summary table of histopathological evaluations from bone marrow (BM) smears and spleen sections. BM smears demonstrated expected cellular morphology and normal levels of hematopoietic precursors across all groups. Mild to minimal extramedullary hematopoiesis (EMH) observed in spleen sections was consistent across experimental groups.
Data are presented as mean ± SD.

applications involving engineered nucleases.[22] In this study, we have taken a comprehensive approach for evaluating the safety and toxicity of our gene edited product manufactured at scale, using a combination of DNA- and RNA sequencing (RNA-seq)-based analyses and pathology assays in a time course fashion, both *in vitro* and *in vivo* (Figure 5A). Sampling and off-target analysis at different time points and especially before and after transplantation allows not only the identification of undesired genetic modifications in the product, but also the assessment of their functional relevance, such as their impact on selection of cells bearing specific off-target modifications or their potential to develop malignancies, or unbalanced hematopoiesis.

To first detect off-target sites in gene edited HSPC products, we performed deep sequencing of 15 off-target sites retrieved using the in silico COSMID software based on sequence similarity with the gRNA and previously screened in WAS patient samples.[6] After validation by NGS, only one site returned significant, although at low frequency, indels in *COL23A1* intron 5 in one HSPC donor source (named as OT1) and as such it was chosen for off-target analysis in large- scale edited cells (Figure 5B). In parallel, further 6 genomic sites retrieved by an unbiased genome wide GUIDE-seq analysis[6] were added to the pool of potential off-target loci to be screened in the large-scale products (OT2-OT7) (Table S1). Importantly, only one potential off-target site was reproducibly retrieved by both platforms. We performed deep sequencing of seven genomic sites on gDNA extracted from large-scale edited CD34$^+$ HSPCs (SU-1 to SU-4) four days post-editing. Significant indels were detected only at the OT1 site, and only in one of the four donor-derived scale-up products treated with either RNP alone or RNP+AAV, when compared to unmanipulated WT controls. (Figure 5C). When the same sites were analyzed on cells cultured for one additional week (day 14 post editing) we could not detect unwanted genetic modification at any of the sites interrogated, suggesting lack of any selection of cells bearing indels at sites other than *WAS* (Figure 5D). To evaluate the emergence of new off-target modifications or the persistence of those observed *in vitro,* indicative of their potential functional relevance, we performed deep sequencing of the seven predicted off-target sites on human CD45$^+$ cells isolated from the BM of mice 14 weeks after transplantation with SU-1 to SU-4 HSPCs. Confirming the data obtained at day 14, the latest time point of *in vitro* culture, indels at off- target sites were not retrieved in most of engrafted cells (Figure 5E); however, we surprisingly did detect indels at the OT1 site from cells retrieved from the BM of one of the 13 mice transplanted with RNP+AAV CD34+ HSPCs from SU-3, despite lack of detection pre-transplant (Figure 5F). Off-target analysis was also performed in the two additional HSPC donor sources used for i53 testing, where we could not detect any non-specific editing at any of the 7 tested genomic sites pre- and post-transplant, further highlighting the overall low off-targeting rates across donor

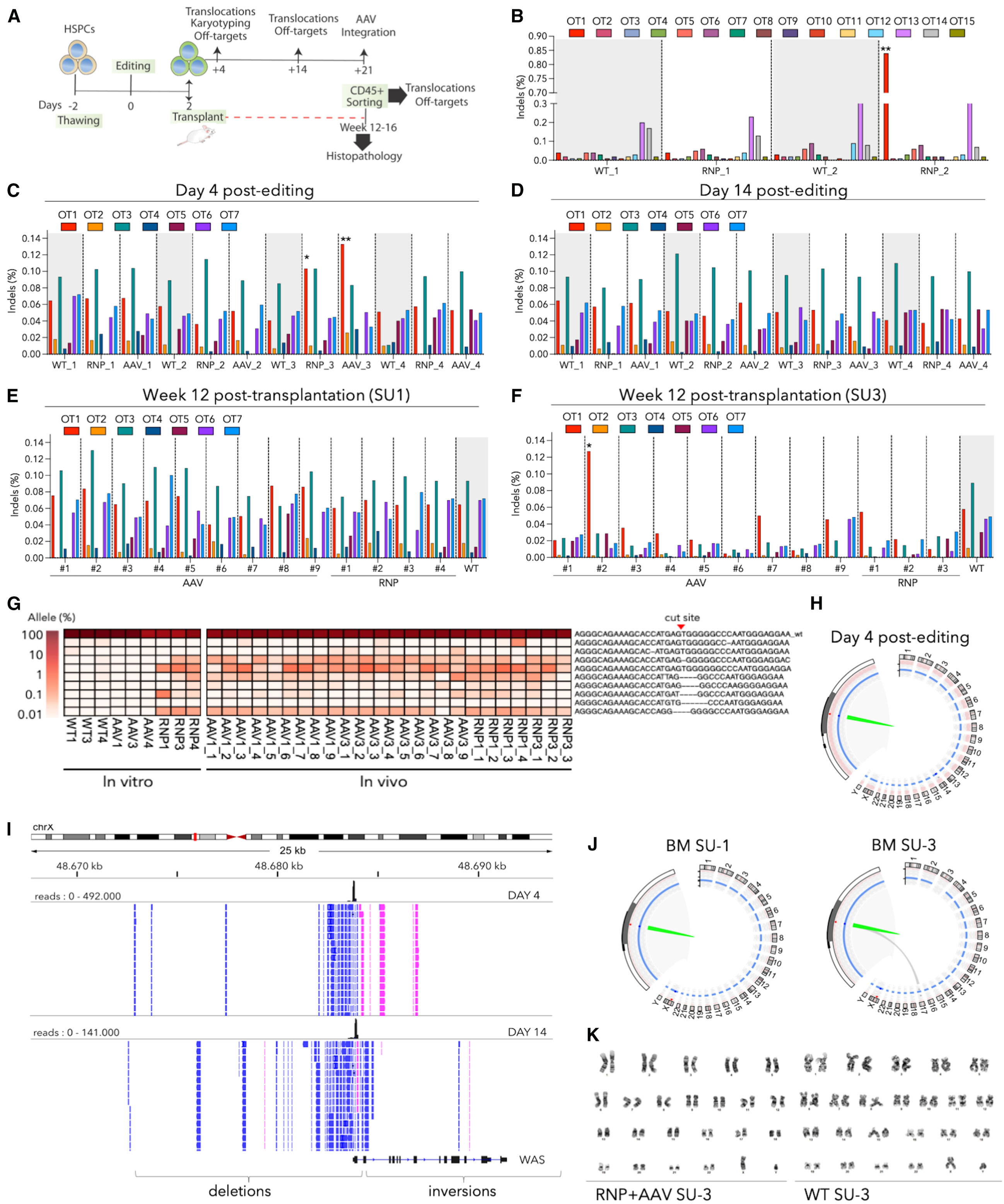

(legend on next page)

sources and following transient 53BP1 inhibition (Figures S9B and S9C).

In parallel, we also performed a mutational tracking analysis of gene edited cells before and after transplantation by assessing the frequency of the top 10 most abundant allele variants at the on-target gRNA cutting site. While in WT and scaled-up RNP+AAV samples we detected mostly alleles with *WAS* wild type sequence, reflecting absence of manipulation and correct coWAS knock-in respectively, RNP samples exhibited enrichment in specific allele variants resulting from NHEJ-mediated DSB repair, which were consistent among donor replicates (Figure 5G). Tracking of these variants in BM samples 14 weeks after transplantation revealed similar patterns of allele frequency without evidence of selection of clones bearing specific indels at the *WAS* locus, while confirming engraftment of HSPCs that underwent on-target gene editing.

Off-target CRISPR-Cas-induced modifications represent just one of many chromosomal rearrangements that can result from targeted gene editing and thus thorough investigation of possible deletions, translocations and gross chromosomal disruptions at on- and off-target sites is mandatory to provide a comprehensive view of the safety of the approach. To assess this, we performed chromosomal aberrations analysis by single targeted linker-mediated PCR sequencing (CAST-seq) analysis on edited HSPCs at day 4 and day 14 post editing using the methodology described previously.[23] CAST-seq technology can detect on- and off-target translocations down to a frequency of 1 in 10,000 cells and searches for genome wide translocations in an unbiased way. By analyzing all manufactured products at both day 4 and day 14 after editing, we could not detect any major chromosomal aberration, further confirming absence of consistent DSB formation at off-target sites with a second, unbiased technology (Figure 5H). We could only observe rare events of large deletions and inversion happening at the on-target *WAS* genomic locus as a consequence of NHEJ-repair; however, as already demonstrated for other sites[23] and as suggested by our mutational tracking analysis, the frequency of these events significantly declined at later culture time point, especially for the inversions, indicating counterselection of cells bearing these rearrangements (Figure 5I). To get a thorough understanding of the functional relevance of large deletions/translocations not detected in the product but that might have appeared *in vivo* upon transplantation, we performed CAST-seq analysis on genomic DNA retrieved from CD45+ engrafted cells in the BM of mice infused with the SU-1 and SU-3 products. While no chromosomal rearrangements were observed in samples retrieved from SU-1-infused mice, we detected a translocation between the on-target site and chromosome 17 in 2 SU-3-infused mice (Figure 5J). The site on chromosome 17 mapped to the 13th intron of the solute carrier family 47 member 2 (*SLC47A2*) gene, which is not a known oncogene. Because the site was neither an off-target nor contained putative gRNA sequences or regions significantly homologous (at least 25bp of homology within a 5-kb window) to the on-target site, this translocation was considered to be prompted by a naturally-occurring break site, likely caused by the strong DNA replication challenge imposed on HSPCs after their transplantation.[24,25] Importantly, deep sequencing confirmed that the translocation happened at very low frequency (Table S2) further validating the safety profile of the platform *in vitro* and most importantly *in vivo*. Karyotype analysis of HSPCs edited at large scale cultured up to 4 days post editing did not reveal any alteration in ploidy or gross chromosomal structural modifications, further confirming the CAST-seq observations (Figures 5K and S10). In summary, these

Figure 5. Comprehensive and longitudinal genotoxicity assessment confirms overall safety of the gene editing platform *in vitro* and *in vivo*

(A) Schematic representation and timeline of the safety study, describing the different assays used to assess genotoxicity *in vitro* and *in vivo*. (B) InDel frequency analysis in WAS patient-derived HSPCs at 15 potential off-target sites retrieved by in silico prediction. On the *x* axis, single samples derived from two different donors (numbered 1–2) are represented; on the *y* axes, the average value from $n = 2$ technical replicates for each off-target site analyzed are represented for each sample; $^{*}p < 0.05$, statistical significance was assessed using *Z* tests corrected by standard deviation calculated from untreated WT controls highlighted in gray in the plot). (C and D) InDel frequency analysis at on-target and predicted off-target sites, performed by targeted amplicon deep sequencing of genomic DNA (gDNA) isolated from edited (RNP and RNP+AAV) HSPCs at day 4 (C) and day 14 (D) post-editing from 4 donors. On the *x* axis, single samples derived from 4 different donors (numbered 1–4) are represented; on the *y* axes, the average value from $n = 2$ technical replicates for each off-target site analyzed are represented for each sample; $^{**}p < 0.01$, $^{*}p < 0.05$, statistical significance was assessed using *Z* tests corrected by standard deviation calculated from untreated WT controls, highlighted in gray in each plot). (E and F) InDel frequency analysis at on-target and predicted off-target sites, performed by targeted amplicon deep sequencing of genomic DNA (gDNA) isolated from CD45+ cells in the BM of mice transplanted with edited (RNP and RNP+AAV) HSPCs from SU-1 ($n = 14$ mice analyzed), (E) or SU-3 ($n = 13$ mice analyzed) (F) On the *x* axis, data derived from each single mouse are represented; on the *y* axes, the average value from $n = 2$ technical replicates for each off-target site analyzed are represented for each mouse/sample; ($^{*}p < 0.05$, statistical significance was assessed using *Z* tests corrected by standard deviation calculated from untreated WT controls highlighted in gray in the plots). (G) Mutational tracking of gene-edited cells pre- and post-transplantation, showing the relative abundance of the top 10 most frequent alleles at the on-target cleavage site. (H) Representative Circos plot of CAST-seq analysis of edited cells at day 4 post-editing, illustrating lack of detection of chromosomal translocations. On-target InDels are shown in green. (I) Qualitative CAST-seq analysis of HSPCs at day 4 and 14 post editing. Representative Integrative Genomics Viewer (IGV) plots illustrate CAST-seq reads surrounding the target site within a window of 25 kb. Mapped CAST-seq reads are represented by bars (only the top 7 lines are shown). Blue and pink bars indicate sequences aligning with the negative or positive strand, respectively. Coverage (i.e., the number of mapped reads) is indicated by histogram plots at the top of the bar plots for each experimental condition. Reads mapped to the left side of the cut site indicate large deletions, while those on the right side indicate inversions or/and translocations with homologous regions. (J) Circos plots representing CAST-seq analysis of CD45+ cells isolated from the BM of NSG mice transplanted with edited (RNP and RNP+AAV) or WT HSPCs from SU-1 and SU-3. CD45+ cells isolated from from $n = 2$ mice per scale-up experiment (SU1 and SU3) were pooled, each utilized in 2 experimental replicates. In SU-3, the plot highlights on-target InDels (green) and a single translocation to chromosome 17 (gray). (K) Representative karyotype of HSPC metaphase spreads analyzed at day 4 post-editing. A total of 270 metaphases from three independent donors were examined, with no chromosomal abnormalities detected.
Data are presented as mean ± SD.

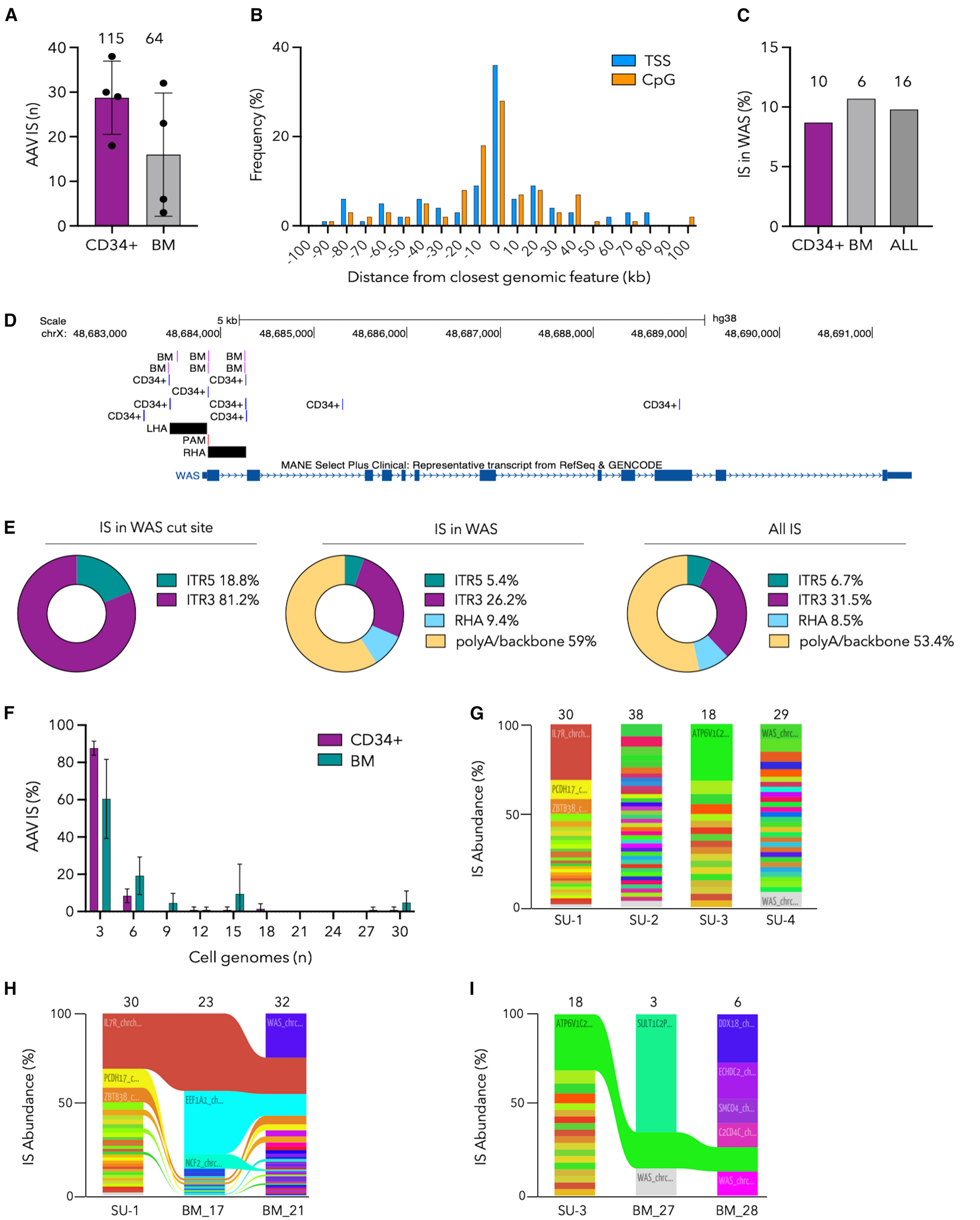

(legend on next page)

(legend on next page)

data highlight the overall safety of our genome editing strategy applied to HSPCs, while underscoring the importance of careful, patient-tailored and continuous assessment of the consequences of CRISPR-Cas activity post transplantation.

AAV donor vector is preferentially trapped at the WAS locus and marks bone marrow repopulating HSPCs

Because our gene editing approach provides HDR-mediated integration of the corrective cassette via AAV transduction, and because many recent reports have documented AAV integration into the host genome and possible related genotoxicity in murine models, we undertook unbiased genome-wide retrieval of AAV6 donor vector integration sites (ISs) in pre- and post-transplantation samples using an established methodology and bioinformatic pipeline.[26] We analyzed genomic DNA from the 4 *ex vivo* edited scale-up products and from BM samples of 4 mice transplanted with SU-1 and SU-3, which were carefully selected based on high engraftment rate of HDR-corrected cells. AAV-genome junctions were amplified using three sets of primers, to identify integration via different portions of the AAV genome (Figure S11A). In the *ex vivo* dataset, we identified 115 non-redundant ISs, ranging from 18 to 39 unique ISs per large scale editing replicate, while in the post transplantation samples a total of 64 ISs were retrieved, with only 9 found in SU-3 transplanted mice, possibly reflecting the lower rate of engraftment in those mice compared to SU-1 ones (Figures 6A and S11B; Table S3). Integrations were found in enriched regions (Figures 6B, S11C, and S11D) and the preferential target for integration was *WAS*, accounting for 9.8% of all the integrations retrieved in the analyzed samples, followed by *SMPDL3B*, which was targeted by 4.3% of integrations (Figure 6C; Table S4). The ISs in *WAS* were either found clustered around the gRNA targeting site, indicative of AAV trapping at the Cas-induced DSB, or around *WAS* endogenous regions also contained in the AAV donor vector homology arms, suggesting integration via homology between vector and host genomic sequences (Figure 6D). These observations were further confirmed when inspecting the regions of AAV found within the amplified AAV-genome junctions; indeed, while all ISs mapping close to the gRNA cutting site contained intact 3′ and 5′ ITRs, indicative of vector trapping within DSBs, the remaining ISs in *WAS* displayed a pattern similar to the ISs retrieved genome-wide, and involved AAV regions such as the polyA, vector backbone or the right homology arm (Figure 6E). Notably, sequences flanking the ISs at *SMPDL3B* show a moderate degree of homology with the right homology arm of the AAV construct, containing part of *WAS* intron 1. Overall, these observations suggest that a portion of the integration events may be driven by sequence homology, a known mechanism in AAV integration.[27] Finally, none of the IS co-mapped with off-target sites either detected by GUIDE-seq or predicted *in silico*, further validating the specificity of our Cas9:gRNA platform.

The employed AAV integration analysis pipeline makes use of fragmented genomic DNA, enabling the estimation of AAV IS abundance by counting the distinct genomic breakpoints.[26] More than 80% and 70% of ISs in *ex vivo*-edited HSPCs and in BM samples respectively were represented by up to 3 genomes/cell, indicating no detectable clonal expansion of AAV IS-bearing cells, within the sensitivity of the technique and duration of the study, when cultured *in vitro* for up to 21 days post editing or when transplanted *in vivo* (Figure 6F). After estimating the number of genomes linked to each AAV IS, we determined the relative abundance of each IS as a percentage of the total ISs identified in each sample. An overall polyclonal pattern was observed in all 4 HSPC scale-up samples (Figure 6G; Table S5), although clones with a relatively higher level of abundance could be identified in the post-transplantation samples because of the small numbers of ISs recovered, such as in the case of BM samples from SU-3 transplanted mice (Figures 6H and 6I; Table S6). Nevertheless, tracking of IS abundance before and after transplantation clearly demonstrated engraftment and long-term repopulating potential of gene edited HSPCs, with many IS-bearing clones being shared between *ex vivo* HSPC and BM retrieved samples (Figures 6H and 6I; Table S7). Analysis of shared ISs between pre- and post-transplant samples also did not provide evidence for genotoxicity or clonal dominance associated with AAV donor integration.

DISCUSSION

In this work, we outline essential preclinical studies aimed at progressing an HDR-based genome editing strategy to treat WAS toward an upcoming phase 1/2 clinical trial. The path from proof-of-concept to clinical application of advanced therapeutic medicinal products (ATMPs) based on the CRISPR-Cas system is complex and currently lacking standardized guidelines from regulatory agencies, resulting in great variability in practices across studies, knowledge

Figure 6. Comprehensive assessment of AAV HDR donor integration in gene-edited HSPCs pre- and post-transplantation

(A) Number of AAV integration retrieved in the scaled-up products (SU-1 to SU-4) *in vitro* and in the BM of mice transplanted with SU-1 and SU-3 HSPCs. (B) AAV integration site distribution around transcription start sites (TSS) and CpG island. (C) Frequency of AAV integrations retrieved within or near the *WAS* locus in *in vitro* samples (CD34+), *in vivo* samples (BM) and in all (ALL) the samples analyzed. The number of unique IS identified in each category is indicated above each column. (D) Representative UCSC Genome Browser snapshot of the *WAS* locus. AAV integration retrieved from edited HSPCs *in vitro* (blue bars) and from CD45+ cells isolated from the BM of transplanted mice (pink bars) are depicted. The gRNA PAM site (red bar) and the left and right homology arms contained in the AAV donor molecule (LHA and RHA, black bars) are also indicated. (E) Pie charts indicating the frequency of AAV features found at the junction breakpoint between the integrated vector and the host genomic region for the different IS datasets as indicated. (F) Bar plot indicating the fraction of cells harboring the same AAV IS number (cell genomes; *x* axis) per each IS mapped (*y* axis), for each dataset (*in vitro* CD34+ and *in vivo* BM). (G–I) Stacked bar plots showing the relative abundance of AAV IS retrieved in HSPCs edited at large scale (G) and in the BM of mice transplanted with SU-1 (H) and SU-3 (I) HSPCs. In each column, every integration is represented by a different color whose height is proportional to the number of genomes retrieved for that specific IS (percentage of IS abundance, *y* axis). The number of unique IS identified in each sample/mouse is indicated above each column. Ribbons connect AAV ISs tracked between samples retrieved before (SU) and after (BM) transplantation. Data are presented as mean ± SD.

fragmentation, and increased research costs, thus posing significant barriers in reaching patients. Our study provides a potential framework, demonstrating one approach to conducting preclinical studies for HSPC-based gene editing strategies in accordance with the latest regulatory guidance on this matter.

Our recent proof-of-concept study provided specificity, toxicity, and efficacy data supportive of continued development of genome editing to treat WAS.[6] The platform relies on CRISPR-Cas9 reagents targeting the start codon of the *WAS* gene and an AAV6 with a codon-optimized *WAS* cassette flanked by *WAS* homology arms, which serves as a template for HDR-mediated repair. We demonstrated that the delivery of editing reagents to WAS HSPCs led to the integration of the therapeutic cassette in the *WAS* locus and to full correction of all functional defects in B- and T-cells, macrophages and platelets both *in vitro* and *in vivo* upon HSPC transplantation. This was achieved through the integration of the corrective gene downstream of the endogenous *WAS* regulatory elements, enabling regulated expression of the WAS protein across all lineages at levels comparable to those in healthy cells. This represents a significant advantage over existing lentiviral-based approaches and underscores the value of site-specific integration achieved through HDR strategies. In this study, we focused on key aspects essential for successful clinical translation, including: (1) the ability to manufacture this ATMP at clinical scale while preserving HSPC fitness and BM repopulating capacity, and (2) the overall safety of the gene editing procedure. The former represents a major challenge in the HDR field, limiting the broader application of these technologies to the treatment of blood disorders that require high levels of donor chimerism following transplantation. Many preclinical reports have indeed highlighted that animals transplanted with *ex vivo* manipulated HSPCs undergoing HDR display an overall lower human engraftment rate than those infused with control cells.[10] Similarly, a clinical trial (NCT04819841) utilizing CRISPR-Cas9 and an AAV6-based HDR template to introduce a functional copy of the β-globin gene for sickle cell disease (SCD)[28] showed promising preclinical results but unsuccessful clinical outcome, as the treated patient experienced incomplete hematopoietic recovery, likely due to cell toxicity associated with the editing process. Evidence indicates that while the frequency of corrected cells *in vivo* remains comparable to input levels soon after transplantation, it markedly diminishes over time, highlighting that primitive long-term HSCs are likely minimally corrected, underrepresented in the transplant product, or impaired in their engraftment, and self-renewal ability post-manipulation.[10,19,29] Therefore, thorough investigation on the quality of the manufacturing product is mandatory for the clinical success of the gene editing strategy proposed. In this study, we established a clinically ready protocol for gene editing that ensures preservation of key quality features of HSPCs when manipulated at large scale. Considering that for a successful autologous stem cell transplant, it is desirable to infuse 2.5–6 $\times$ 10^6/kg (European Society of Bone Marrow Transplantation guidelines),[30] and that higher doses (5–10 $\times$ 10^6/kg or more) have been associated with better engraftment and reduced complications,[31,32] we edited an average of 110 $\times$ 10^6 cells per each donor source used to mimic a possible manufacturing scenario. By using GMP-grade reagents and optimized culture and electroporation procedures, we were able to achieve HDR rates comparable to those observed when editing at medium and small scales, while attaining viability rates comparable to unmanipulated cells both in the fresh and in the cryopreserved product. Most importantly, large-scale edited cells and control cells generated comparable numbers of colonies in solid cultures and engrafted the bone marrow of recipient mice at similar frequencies, supporting preservation of HSPC repopulating activity in primary recipients despite *ex vivo* genetic manipulation. Overall, all 4 manufacturing runs met or exceeded the batch release criteria set out by our GMP unit at the Great Ormond Street Hospital NHS Foundation Trust (UK) for the release of gene therapy-based ATMPs, including cell purity, viability, editing frequency and colony-forming capacity, confirming the robustness and suitability of the devised protocol. Notably, fine-tuning of the electroporation procedure to deliver the CRISPR-Cas RNP complex yielded minimal cell toxicity as highlighted by all QC tests and confirmed by RNA-seq analysis and engraftment rates *in vivo*, representing a significant improvement compared to other reports.[33,34]

A key limitation of our platform is the consistent decline in the percentage of gene-corrected cells following engraftment, a challenge commonly observed in approaches that rely on harnessing the HDR pathway for HSPC correction, as previously discussed.[22] This defect is likely caused by activation of a DNA damage response and senescence-like cell phenotype mediated by p53-and p38 mitogen-activated protein kinase (MAPK)-reactive oxygen species (ROS) signaling pathways.[8,11,13] RNA-seq analysis performed on CD34+ HSPCs edited at large scale compared to unmanipulated controls confirmed the presence of gene expression dysregulation pointing to disruption of cellular pathways linked to apoptosis, cell-cycle arrest, and inflammation in our edited products. Because the use of an AAV donor molecule has been indicated as one of the main culprit[9,11] and because partial reversion of the phenotype and preservation of HSPC function has been demonstrated when pharmacological intervention targeting these pathways has been employed, we further optimized our *ex vivo* gene editing protocols by reducing the AAV burden while increasing HDR via transient 53BP inhibition.[20] The addition of i53 mRNA alone improved outcomes, enhancing colony-forming capacity in semisolid cultures and significantly increasing the number of engrafted, gene-corrected cells in hematopoietic organs. However, the combination of 53BP1 inhibition with a 10-fold reduction in AAV dose yielded even greater benefits, resulting in up to a 5-fold increase in the frequency of engrafted $CD34^+$ cells carrying the *WAS* cassette knock-in. Future work may explore the incorporation of inflammation and senescence inhibitors to the culture media and/or their combination with inhibitors of the TP53 pathway to further boost HSPC fitness while maintaining or increasing knock-in efficiency.[8,13,19] While the results may be skewed by the oligoclonal nature of the mouse model used and as such the definitive ability of edited cells to reconstitute a healthy hemopoiesis post transplantation will only be determined in patients, this piece of data emphasizes the need of careful fine-tuning of manufacturing

protocols to achieve therapeutic outcomes, while shedding light on a promising route to preserve HSPC function paving the way for more effective CRISPR therapies.

Despite the efficiency and versatility of the CRISPR-Cas system, unintended genetic modifications at off-target sites remain a major safety challenge. These off-target edits, which may involve the generation of indels, have the potential to interfere with gene expression and regulation, sometimes leading to harmful consequences such as oncogenic changes. Additionally, genome instability can arise from larger structural alterations at both intended and unintended sites, which can ultimately result in chromosomal loss.[35,36] Therefore, comprehensive profiling of off-targeting events is needed before reaching clinical translation. Despite the development of a large plethora of methodologies to detect such genomic edits, no single method can exhaustively characterize the off-target spectrum in the cell product[37] and as such the use of orthogonal techniques has been recommended by the Food and Drug Administration. In this study, we evaluated genotoxicity in a comprehensive way by combining in silico prediction with a genome-wide, unbiased and *in cellulo* GUIDE-seq method to detect off-targeting, and complemented this with genome stability assessment through CAST-seq, karyotyping and analysis of AAV donor vector integration. The proposed framework is aligned and in several aspects exceeds the depth of analysis performed for the market authorization of Casgevy,[38] although future Investigational New Drug (IND)-enabling studies may foresee the incorporation of additional biochemical approaches to *in cellulo* assays for off-target nomination to enhance the depth of genomic characterization.[16] Off-target detection conducted in edited CD34+ HSPCs from 3 WAS donors and 6 healthy donors revealed an overall favorable safety profile for the selected gRNA targeting *WAS*, but highlighted the need of a personalized approach that takes in consideration Cas9 cutting variance across individuals. Indeed, although Cas9 off-target cutting varies between patient-derived cells primarily because genetic differences at the off-target locus (e.g., SNPs/indels affecting guide complementarity or PAM sequences), epigenetic context, cell state, and DNA repair activity further modulate whether cutting occurs and is detectable.[39–41] Reflective of this, 1 WAS patient and 1 healthy cell donor out of 7 showed significant off target editing at the fifth intron of *COL23A1* in the product examined *in vitro*, with one of them showing emergence of the same pattern 14 weeks after transplantation in one mouse. The same emerged from the CAST-seq analysis, where a low frequency translocation between the on-target and a natural break site on chromosome 17 was detected only in mice transplanted with cells derived from one specific healthy donor, but not in the cryopreserved product before transplantation. This observation also underscores the importance of evaluating the progression and functional implication of a CRISPR-Cas-induced mutation when detected in the manufactured product, which was tackled in this study by an orthogonal and longitudinal analysis at different time points. Indeed, most off-target mutations tend to arise in intronic or other non-coding regions, where they generally pose little risk to cellular function.[42] When mutations do occur in exonic regions, they are often loss-of-function variants that are naturally eliminated through negative selection, making their persistence in the patient unlikely. In contrast, gain-of-function mutations are more worrisome, as they may confer a growth advantage that drives clonal expansion and potentially contributes to cancer development. In our case, the intronic off-target site detected in one scale-up product was lost over the 10-day culture time *in vitro*, suggesting negative selection. However, the same off-target site was found edited in the BM of one out of 12 examined mice 14 weeks post-transplantation, indicating that despite its low frequency, the mutation was harbored by a fraction of BM-repopulating CD34+ HSPCs and was maintained in an *in vivo* setting. This underscores the critical need for patient monitoring following transplantation, as further emphasized by the detection of a de novo chromosomal rearrangement, between the on-target site and a natural DNA break site, observed only after cell engraftment in mice. This rearrangement was likely driven by the proliferative stress placed on a limited pool of edited HSPCs during hematopoietic reconstitution,[24] a phenomenon that might be prevalent in many gene editing clinical trials where a limited number of corrected HSPCs are able to engraft. Whether these undesired genomic aberrations will lead to clonal dominance and tumorigenesis in the patients, it will only be determined with long-term patient surveillance post transplantation. Nevertheless, in our 14 weeks-long *in vivo* experiments, we could not detect any sign of cell and tissue toxicity, as evidenced by the well-being of transplanted mice and absence of lesions in the hematopoietic organs analyzed by immunohistochemistry. Analysis of the BM of mice transplanted with i53-treated HSPCs also did not reveal any insurgence or selection of off-targets bearing clones, as previously observed[12,14]; while we acknowledge that more comprehensive analyses are needed to fully exclude potential long-term effects, particularly in human settings, the transient nature of p53/53BP1 pathway modulation likely limits the risk of sustained disruption of the DNA damage response.[12,14,19,43] Tracking of indels variants at the *WAS* on target locus before and after transplantation did not reveal evidence of skewed proliferation of cells bearing specific mutations at the genomic site, indicating that infusion of edited CD34+ HSPCs that underwent NHEJ-rather than HDR-mediated correction of generated DSBs is likely not harmful to the patient; this observation was also confirmed by counter selection of indels-bearing CD34+ HSPCs *in vivo*. Finally, our comprehensive preclinical study contributed to the characterization of the impact of AAV integration to HSPC engraftment potential and overall genotoxicity, an aspect that has been largely overlooked by the HDR field. Although detection was limited only to a fraction of cells bearing integration of the donor molecule and only to primary recipients, tracking of AAV integration before and after transplantation further highlighted no evidence of clonal selection and abnormal expansion of edited CD34+ HSPCs *in vivo*, with a polyclonal integration pattern and no integrations detected into or nearby oncogenes. Nevertheless, long-term repopulating HSC fitness, potential delayed clonality and consequences of rare off-target and structural variants remain to be fully established

through secondary transplantation and extended post-treatment surveillance in patients.

In summary, we have reported the establishment of a clinically scalable and regulatory-compliant CRISPR-Cas9-AAV6 HDR gene editing platform for diseases that could be treated by *ex vivo* HSPC gene editing, demonstrating functional correction of HSPCs and emphasizing the importance of optimized manufacturing protocols and thorough genotoxicity assessments to ensure efficacy, safety, and long-term engraftment potential. The study outlines a structured, regulatory-aligned approach for translating CRISPR-Cas9-AAV6 HDR strategies into clinical trials, and sets a precedent for regulatory-grade product characterization, potentially serving as a model for other gene-editing therapies.

MATERIALS AND METHODS

Study design

The aim of this preclinical study was to assess efficacy, safety, and clinical-scale manufacturing feasibility of the manufactured product which uses *ex vivo* Cas9-RNP coupled to rAAV6-mediated coWAS donor cassette delivery to correct mutations in the WAS gene in human HSPCs. Our main goal was to comprehensively determine the safety of our gene editing platform to translate it into the next generation of therapeutic tools for WAS.

Ethics and animal approval

For usage of human CD34+ HSPC from healthy and WAS donors, informed written consent was obtained in accordance with the Declaration of Helsinki and ethical approval from the Great Ormond Street Hospital for Children NHS Foundation Trust and the Institute of Child Health Research Ethics (08/H0713/87).

For experiments involving animals, NOD-SCID-IL2Rg$^{-/-}$ (NSG) mice were purchased from Charles River and maintained in specific-pathogen-free (SPF) conditions. Mice were housed in a 12 h day/12 h night cycle with controlled temperature and humidity. The ventilated cages had sterile bedding and everyday supply of sterile food and water in the animal barrier facility at University College London. The procedures involving mice were designed and performed in accordance with UK Home Office regulations, and experiments were conducted after approval by the University College London Animal Welfare and Ethical Review Body (project license 70/8241).

AAV6 vector design, production, and purification

GMP compliant therapeutic AAV6 vector plasmid was generated by cloning codon-optimized 1.5-kb-long WAS cDNA (coWAS) flanked by 405 bp long left and right homology arms described previously[6] into the pBB4-MCS plasmid (Dr. Leszek Lisowski, University of Sydney), with inverted terminal repeats derived from AAV2.

AAV6 vectors were either purchased from Vigene Biosciences or produced in-house as described previously.[6] Briefly, producer AAVPro293T cells (Takara Bio; Cat# 632273) were thawed at 37°C and expanded in Dulbecco's modified Eagle's medium (DMEM) supplemented with 10% fetal bovine serum (FBS) and 1% penicillin/streptomycin. The cells were expanded and plated in 10 cm plates a day before transfection. 24 h later, producer cells were co-transfected with the donor template plasmid (described previously) and pDGM6 Rep/Cap helper plasmid, using a polyethylenimine-based transfection method with a change of medium performed the next day. At 72 h after transfection, the resulting cell lysates and purified media were purified using iodixanol gradients-based ultracentrifugation. Viral genomic titer was determined by ddPCR using ITR-specific primers and probes as listed in Table S2. The final coWAS_AAV6 preps had an average titer of 1×10^{13} vg/mL, which were subsequently aliquoted and stored at −80°C until use.

Isolation and *in vitro* culture of CD34+ HSPCs

Human HSPCs were isolated by the immunomagnetic selection of CD34+ HSPCs from G-CSF mobilized apheresis products from male healthy donors (Caltag Medsystems and All cells). Before CD34+ selection, the apheresis product was washed with CliniMACS PBS/EDTA buffer containing 0.5% (v/v) human serum albumin to remove platelets. CD34+ HSPCs were then either selected manually (SU1&2) or isolated by positive selection on the CliniMACS Plus instrument (Miltenyi Biotec, Bergisch Gladbach, Germany) for SU3&4. Isolated HSPCs were either freshly plated in culture (SU2-4) or cryopreserved in Cryostor freezing medium (CS5; STEMCELL Technologies) (SU-1) and thawed at the time of experiment. HSPCs were cultured at a cell density of 0.5×10^6/mL in StemSpan Serum-Free Expansion Medium II (STEMCELL Technologies, Vancouver, Canada) supplemented with human cytokines: stem cell factor (SCF, 100 ng/mL), thrombopoietin (TPO, 100 ng/mL), Fms-like tyrosine kinase 3 ligand (Flt3-L, 100 ng/mL), interleukin-6 (IL-6, 100 ng/mL), IL-3 (30 ng/mL) and penicillin-streptomycin (20 U/mL), stemregenin1 (0.75 M; STEMCELL Technologies, Vancouver, Canada) and UM171 or UM729 (35 nM; STEMCELL Technologies, Vancouver, Canada).

Gene correction procedure and associated analyses

GMP complaint synthetic sgRNA containing 2-O-methyl-3′-phosphorothioate at the three terminal positions at both 5′ and 3′ ends targeting *WAS* locus was provided by synthego. The *WAS* sgRNA was >99% pure and the target sequence for is 5′-GCAGAAAGCACCATGAGTGGGGG-3′. GMP SpyFi Cas9 nuclease was purchased from Aldevron (Fargo, ND, USA). CD34+ HSPCs were either thawed or cultured from fresh apheresis in the Stemspan SFEMII medium supplemented with early active cytokine cocktail (SCF, TPO, Flt3-L, IL6, and IL3) along with UM171 and Stemregenin-1 for a period of 48 h. RNP electroporation was performed at day 2 using the Maxcyte electroporator. Briefly, the cells were collected, counted, and spun at 300 g for 5 min, following which the supernatant was discarded carefully and the cell pellet was washed with pre-warmed 1 mL of Maxcyte electroporation buffer (HyClone). Large-scale RNP electroporations were performed using a CL1.1 cartridge (suitable for 100–700 M cells) in a total volume of 1.8 mL and with an OC-100 cartridge in a total volume of 100 μL for medium

scale (program HSC-3). Electroporated cells were then plated at a cell density of 1.25×10^6 cells/mL in the cytokine-supplemented medium as described earlier. After 15 min, rAAV6 carrying the coWAS cassette was dispensed onto cells at multiplicities of infection of 2.5×10^4 vector genomes per cell based on titers determined by ddPCR and incubated for 24 h. After 24 h, a medium exchange was performed to dilute or remove any residual coWAS-AAV6, and the CD34+ HSPCs were cultured for an additional 12–24 h before cryopreservation. Additionally, a subset of cells from the three groups was cultured for an additional 2–3 weeks for *in vitro* genotoxicity analysis.

In vitro transcription (IVT) of i53 mRNA

A codon-optimized sequence encoding the 53BP1 inhibitor peptide (i53) was cloned into an IVT backbone vector (Addgene #205251) downstream of the T7 promoter, using NheI/BsmBI at the 5′ end and NotI at the 3′ end to preserve the 3′ UTR and poly(A) tail.

IVT was performed using the MEGAscript T7 Transcription Kit (Thermo Fisher Scientific, AM1334) with full substitution of Uridine with Pseudouridine (TriLink BioTechnologies) and co-transcriptional capping with CleanCap AG (TriLink BioTechnologies). Reactions were incubated at 37°C for 4 h, after which TURBO DNase was added and incubated for a further 15 min at 37°C. mRNA was purified and concentrated using the RNA Clean and Concentrator-100 kit (Zymo Research) according to the manufacturer's instructions and stored at −80°C until use. RNA quality and integrity were confirmed by TapeStation analysis. The resulting i53 mRNA (∼260 nucleotides) was included in the RNP electroporation mixture at the time of HSPC nucleofection, as previously described.[12,14]

Methylcellulose CFU assessment

At day 1 post editing, the HSPCs from the transduced and the control groups were plated into 12-well plates containing MethoCult (STEMCELL Technologies, Vancouver, Canada).

Two weeks later, colonies were appropriately scored based on morphological appearance in a blinded manner. Subsequently, colonies were harvested and lysed for genomic DNA extraction followed by ddPCR analysis for targeted integration of WAS cassette.

Targeted integration analysis by ddPCR

For the measurement of targeted integration of codon-optimized WAS cDNA in the edited samples, ddPCR was performed as previously described.[6] Briefly, genomic DNA was extracted using DNeasy blood and tissue kit (QIAGEN; Cat# 69504) and a total of 60 ng was mixed with 10 μM each of target primer and FAM probe mix, 10 μM each of reference primer and HEX probe mix, 1× ddPCR Supermix probe without dUTP (Bio-Rad, UK) and nuclease-free water. The primers and probes sequences are detailed in Table S2. The individual droplets were generated using QX100 Droplet Generator (Bio-Rad) and subsequently amplified in a Bio-Rad PCR thermocycler. The optimized amplification steps were: step 1: –95°C for 10 min; step 2: (49 cycles)—94°C for 1 min, 60°C for 30 s, 72°C for 2 min; and step 3: 98°C for 10 min. The Droplet Reader and QuantaSoft Software (both from Bio-Rad) were used to record and analyze the positive and negative fluorescence droplets according to the manufacturer's guidelines. The percentage of integration was calculated as the ratio of FAM to HEX signal after normalization against the reference signal.

Karyotype analysis

CD34+ HSPCs from healthy mobilized PB were electroporated as described earlier. After 2–4 days in culture, $3–5\times 10^6$ cells from edited and transduced group as well as WT control cells were incubated overnight with 0.6 μg/mL of mitotic inhibitor Colcemid (KaryoMAX Colcemid Solution in PBS Life Technologies, Carlsbad, CA), thereafter incubated in hypotonic solution (0.075 M potassium chloride (KCl) added dropwise) at 37°C for 25 min, followed by the dropwise addition of the fixative (3:1 methanol/acetic acid). Karyotype analysis was then outsourced to Cambridge Biosciences. The metaphase spreads were aged at room temperature for 5 days and G-banding were performed following standard methods with a few modifications: slides were incubated in Sorensen buffer at room temperature for 2 min, followed by 2 min in Wright stain solution (0.5 mL Wright stain and 1.5 mL of Sorensen buffer). A total of 270 metaphase spread images from three independent HSPC donors and editing experiments were acquired with a Leica DM-2500 Bright Field Microscope and the images were analyzed using the software Cytovision v.7.7.

Genome-edited CD34+ HSPC xenotransplantation studies in NSG mice

Edited cells as well as WT and RNP controls frozen 36–48 h post editing as detailed previously were thawed and 5×10^6 viable cells were injected i.v. into each NSG mouse after sublethal irradiation (2Gray) using a 27 gauge × 0.5-inch needle. Eight weeks post transplantation, mice PB from tail vein was lysed with 1× Red Blood Cells (RBC) lysis buffer (Thermo Fisher Scientific) and stained with anti-human CD45 APC antibody (clone HI30, BioLegend) to evaluate human CD45+ engraftment by fluorescence-activated cell sorting (FACS). At week 14 post transplantation, level of human engraftment and lineage composition were determined in the bone marrow (BM), PB, spleen, and thymus. Briefly, BM cells were harvested by flushing tibiae and femurs with 1× PBS and passing through 40 μm strainer. Mononuclear cells were blocked with Fc blocking solution (BioLegend) and stained with the following anti-human antibodies: CD45 PerCP Cy5.5 (clone HI30, BioLegend), CD19 APC Cy7 (clone HIB19, BioLegend), CD33 PECy7 (clone 67.6, BioLegend), and CD3 APC (clone OKT3, BioLegend) for FACS analysis. To determine human stem cell composition within BM, cells were stained with the following antibody panel: CD34 BV421 (clone 561, BioLegend), CD45 PerCP/Cy5.5 (clone HI30, BioLegend), CD38 APC-Cy7 (clone HIT2, BioLegend), CD90 PE-Cy7 (clone 5E10, BioLegend). Spleens and thymi were grinded against 40 μm strainer and stained for the lineage antibody panel and CD19 APC Cy7 and CD3 APC Abs, respectively.

Histopathological assessment of bone marrow and spleen from transplanted animals

Histopathological analysis on BM smears and spleen sections obtained from transplanted NSG mice was carried out by Histologix in GLP conditions. Spleen samples were fixed in 10% neutral buffered formalin (NBF) for 24–48 h before being transferred to 70% ethanol. For sectioning, 5 μm sections were taken for each spleen sample, picked up on Snowcoat slides, and dried at 37°C overnight. The spleen tissues were trimmed to standardized trimming planes, placed in tissue cassettes, and processed to paraffin wax and sectioned. The slides were stained with hematoxylin and eosin (H&E) and digitally scanned at 20× magnification using a Hamamatsu NanoZoomer v.2. The BM smear preparations were sprayed fixed on to the slide using CytoFix pump spray (CellPath Ltd, UK) and were stained with H&E for pathology review and scanning as mentioned above.

Off-target analyses

Frozen male PB-derived CD34+ HSPCs were cultured for 2 days prior to electroporation with RNP complex as described earlier. At day 4 and 14 post RNP electroporation, genomic DNA was extracted and PCR was performed using off-target primers for the combined sites retrieved from COSMID and Guide-seq.[6] PCR amplicons of WT cells were taken as negative untreated control. Forward and reverse off-target primers were designed around the predicted off-target sites in order to amplify fragments of ∼200–300 bp (Table S2). The PCR-purified amplicons were subjected to end repair, adaptor ligation and an indexing PCR using the NEBNext Ultra II DNA library prep kit for Illumina (New England BioLabs) according to the manufacturer's instructions. The denatured amplicons were loaded at 12 pM into the Illumina MiSeq Reagent Kit V2 - 500 cycle according to the manufacturer's instruction. The FASTQ files were analyzed for indels using the command line version of CRISPResso49 considering 40 bp around the supposed cleavage site and not considering substitutions to reduce background signals. The derived INDEL proportions of the treated samples were statistically compared to the corresponding untreated values in a one-tailed Z-test. For this purpose, the RNP treated values were corrected by the standard deviation of the untreated samples to account for the variability of the measurements.

Detection of translocations: CAST-seq

CAST-seq was performed as described in Turchiano et al. 2020.[23] Genomic DNA was harvested from the sample before and after xenotransplant, fragmented and linker ligated using the NEBNext Ultra II FS DNA Library Prep Kit and NEBNext Ultra II Ligation Master Mix and Ligation Enhancer (New England Biolabs). Specific primers were designed for the WAS locus: WAS_Bait: TCCAAGCATCTCAAAGAGTC and WAS_Decoy: GCAGGGTAAGAAGAGGAAC for the first PCR; and WAS_Nested: GACTGGAGTTCAGACGTGTGCTCTTCCGATCTGGAGGGTATGTTCTGCTGAACC for the second PCR. For CAST-seq of the *ex vivo* samples, 500 ng of gDNA was used as starting material, while for the samples post-transplantation 20–100 ng were used. The PCR reactions were purified and barcoded with NEB Next Multiplex Oligos from Illumina (New England Biolabs). AMPure XP beads purification (Beckman Coulter) was set at 0.8× volume to select fragments >180 bp. The libraries were sequenced through the Illumina MiSeq platform and analyzed with the CAST-seq pipeline (https://github.com/AG-Boerries/CAST-Seq) calling as true mutation events sites that were significantly enriched with respect to the UT control and present in at least two replicates.

Indel deep sequencing analysis for mutational tracking

Off-target sites identified with the CAST-seq, GUIDE-seq, and COSMID tool were subjected to direct PCR amplification and deep sequencing. Amplicons of ca. 300 bp were barcoded using NEBNext_Ultra II DNA library prep kit for Illumina and sequenced through the Illumina MiSeq platform. The sequencing adapters were trimmed using cutadapt[44] using the parameters -j 5 -m 20 -u -1 -U -1. The reads were subsequently analyzed using the CRISPResso2 pipeline,[45] considering an indel window of 10 bp around the putative cleavage site and disregarding substitutions (parameters: –quantification_window_size 10 –ignore_substitutions). The indel proportions obtained from the edited samples were compared with their relative untreated control by means of the one-tailed *Z* test corrected by the standard deviation from the untreated control proportions to account for measurement variability. The mutation tracking analysis was derived by parsing the variant frequency data generated by CRISPResso2 and intersecting the mutation across samples with R package. Any variant which was marked as "unedited" by CRISPResso2 was considered WT for the target site and any variant not detected in a particular sample was given a value of zero. For visualization, the frequency of each variant from sample WT was subtracted from the frequency of that variant in every other sample. The selection of the top 10 up- and down-represented variants was based on the difference between sample WT and the mouse-derived samples. Heatmaps were generated with the pheatmap package.[46]

IS distribution analysis

To assess AAV integration, we adopted a sonication-based linker-mediated PCR method (SLiM), as previously described.[26] Briefly, genomic DNA was sheared using a Covaris E220 Ultrasonicator (Covaris Inc),[47,48] generating fragments with a target size of 1,000 bp. The fragmented DNA was subjected to end repair, 3′ adenylation, and ligation (NEBNext Ultra DNA Library Prep Kit for Illumina, New England Biolabs) to custom linker cassettes (LCs; Integrated DNA Technologies). LC sequences contained an 8-nucleotide barcode for sample identification. Ligation products were subjected to 35 cycles of exponential PCR with primers (available upon request) complementary to different regions of the AAV genomes and to the LC. For each set of AAV-specific primers, the procedure was performed using ∼50–100 ng of sheared DNA. Then, 10 additional PCR cycles were run to include the sequences required for sequencing and a second 8-nucleotide DNA barcode. PCR products were quantified via qPCR using the Kapa Biosystems Library Quantification Kit for Illumina, following the manufacturer's instructions. qPCR was performed in triplicates on each PCR product diluted

10:3, and the concentrations were calculated by plotting the average cycle threshold values against the provided standard curve. Finally, the amplification products were sequenced using Illumina Next/Novaseq platforms (Illumina). After sequencing, a dedicated bioinformatics pipeline, recombinant adeno-associated vector integration analysis (RAAVioli), was used to analyze the amplified sequences for integration site identification.[49] Briefly, a custom Python software was used to identify integration loci and vector rearrangements using concise idiosyncratic gapped alignment report string. Reads characterized by the same AAV/human genome break point and the same number and type of AAV rearrangements were considered identical ISs. Potential indels between the AAV junction and genomic locus were considered to distinguish 1 or 2 ISs: 2 reads from the same PCR reaction were assigned to the same IS if both alignments on human genome and the AAV junctions are aligned within a window of 20 bases. The abundance of each IS was measured by counting, for the same vector/cell genome junctions (IS), the number of different DNA fragments containing a genomic segment variable in size depending on the shear site position, and that will be unique for each different cell genome contained in the starting cell population. Therefore, the number of different shear sites assigned to an IS will be proportional to the initial number of contributing cells in the population studied, thus the clonal abundance of each IS in the starting sample does not consider the biases introduced with PCR amplification. Only integration events identified by at least 2 independent DNA fragments were considered in the analyses, to remove potential PCR artifacts.

Statistical analysis

All statistical tests on experimental groups were done using Prism7 GraphPad Software. The exact statistical tests used for each comparison are noted in the figure legends. For making multiple comparisons, one-way ANOVA Kruskal-Wallis multiple comparisons test with one variable or two-way ANOVA with Bonferroni post-test in case of two variables. For comparing the average mean of two sample groups, we used the unpaired Student's *t* test to reject the null hypothesis ($p < 0.05$). For *in vivo* experiment analyses where different CD34+ cell donors were used and transplanted into mice, statistical analyses were performed using a mixed-effects model (REML) in GraphPad Prism, treating donor as a random effect and experimental condition as a fixed effect. Individual data points corresponding to each donor and mouse are shown in the relevant plots.

DATA AND CODE AVAILABILITY

The data supporting the findings of this study are available within the article and its supplementary materials. Additional data or materials, including protocols and reagents, are available from the corresponding author upon reasonable request.

ACKNOWLEDGMENTS

We would like to thank all past and current members of the Cavazza and Santilli groups for their contribution in conceptualizing this study and experimental support. We thank Dr. Ayad Eddaoudi and his team (Flow Cytometry Core Facility, University College London) for assistance with flow cytometry and the team at UCL Genomics Core Facility, University College London for assistance with NGS sequencing. We also thank Laura Rudilosso (San Raffaele Telethon Institute for Gene Therapy, Italy) for her experimental contribution to AAV integration analysis. This research, A.C., and A.N. were supported by the GOSH Children's Charity-LifeArc Therapeutic Accelerator Grant (VS0420), the Sparks-GOSH Children's Charity Grant (V4522), the Wellcome Trust (217112/Z/19/Z), and the Fondo Italiano per la Scienza (FIS2) consolidator grant awarded by the Italian Ministry of University and Research (FIS-2023-00171). T.E.W. was supported by the Jeffrey Modell Foundation. G.S., A.J.T., and A.C. were also supported by the NIHR Biomedical Research Center at the Great Ormond Street Hospital for Children NHS Foundation Trust and University College London.

AUTHOR CONTRIBUTIONS

A.N. designed and performed experiments, analyzed, and illustrated data; W.V., T.E.W., F.Z., R.G., E.G.C., C.C., G.T., and D.C. performed experiments and analyzed data; H.A. contributed to the design of experiments and illustrated data; E.M., A.J.T., G.S., D.C., G.T., and A.C. contributed to the design and conceptualization of the study or parts of it; A.C. designed and supervised the study and the experiments, analyzed data, provided funding, and wrote the manuscript. All authors reviewed the manuscript.

DECLARATION OF INTERESTS

The WAS gene editing platform described in this study was licensed out by University College London (A.C., A.N., G.T., and A.J.T.) to Danaus Pharmaceuticals. A.C., A.N., G.T., and A.J.T. may receive royalties from Danaus Pharmaceuticals. A.J.T. is on the Scientific Advisory Board of Orchard Therapeutics, Generation Bio, Carbon Biosciences, and 4BIO Capital. G.T. is presently employed by AstraZeneca and may be AstraZeneca shareholders. E.G.C. is presently employed by Glaxo Smith Kline and may be Glaxo Smith Kline shareholders.

SUPPLEMENTAL INFORMATION

Supplemental information can be found online at https://doi.org/10.1016/j.omta.2026.201816.

REFERENCES

1. Allen, D., Knop, O., Itkowitz, B., Kalter, N., Rosenberg, M., Iancu, O., Beider, K., Lee, Y.N., Nagler, A., Somech, R., and Hendel, A. (2023). CRISPR-Cas9 engineering of the RAG2 locus via complete coding sequence replacement for therapeutic applications. Nat. Commun. *14*, 6771. https://doi.org/10.1038/s41467-023-42036-5.
2. Bahal, S., Zinicola, M., Moula, S.E., Whittaker, T.E., Schejtman, A., Naseem, A., Blanco, E., Vetharoy, W., Hu, Y.T., Rai, R., et al. (2024). Hematopoietic stem cell gene editing rescues B-cell development in X-linked agammaglobulinemia. J. Allergy Clin. Immunol. *154*, 195–208.e8. https://doi.org/10.1016/j.jaci.2024.03.003.
3. Castiello, M.C., Brandas, C., Ferrari, S., Porcellini, S., Sacchetti, N., Canarutto, D., Draghici, E., Merelli, I., Barcella, M., Pelosi, G., et al. (2024). Exonic knockout and knockin gene editing in hematopoietic stem and progenitor cells rescues RAG1 immunodeficiency. Sci. Transl. Med. *16*, eadh8162. https://doi.org/10.1126/scitranslmed.adh8162.
4. Dever, D.P., Bak, R.O., Reinisch, A., Camarena, J., Washington, G., Nicolas, C.E., Pavel-Dinu, M., Saxena, N., Wilkens, A.B., Mantri, S., et al. (2016). CRISPR/Cas9 beta-globin gene targeting in human haematopoietic stem cells. Nature *539*, 384–389. https://doi.org/10.1038/nature20134.
5. Fañanas-Baquero, S., Quintana-Bustamante, O., Dever, D.P., Alberquilla, O., Sanchez-Dominguez, R., Camarena, J., Ojeda-Perez, I., Dessy-Rodriguez, M., Turk, R., Schubert, M.S., et al. (2021). Clinically relevant gene editing in hematopoietic stem cells for the treatment of pyruvate kinase deficiency. Mol. Ther. Methods Clin. Dev. *22*, 237–248. https://doi.org/10.1016/j.omtm.2021.05.001.
6. Rai, R., Romito, M., Rivers, E., Turchiano, G., Blattner, G., Vetharoy, W., Ladon, D., Andrieux, G., Zhang, F., Zinicola, M., et al. (2020). Targeted gene correction of human hematopoietic stem cells for the treatment of Wiskott - Aldrich Syndrome. Nat. Commun. *11*, 4034. https://doi.org/10.1038/s41467-020-17626-2.
7. Rai, R., Steinberg, Z., Romito, M., Zinghirino, F., Hu, Y.T., White, N., Naseem, A., Thrasher, A.J., Turchiano, G., and Cavazza, A. (2024). CRISPR/Cas9-Based Disease Modeling and Functional Correction of Interleukin 7 Receptor Alpha Severe Combined Immunodeficiency in T-Lymphocytes and Hematopoietic Stem Cells. Hum. Gene Ther. *35*, 269–283. https://doi.org/10.1089/hum.2023.100.
8. Della Volpe, L., Midena, F., Vacca, R., Tavella, T., Alessandrini, L., Farina, G., Brandas, C., Lo Furno, E., Giannetti, K., Carsana, E., et al. (2024). A p38

MAPK-ROS axis fuels proliferation stress and DNA damage during CRISPR-Cas9 gene editing in hematopoietic stem and progenitor cells. Cell Rep. Med. *5*, 101823. https://doi.org/10.1016/j.xcrm.2024.101823.

9. Ferrari, S., Jacob, A., Cesana, D., Laugel, M., Beretta, S., Varesi, A., Unali, G., Conti, A., Canarutto, D., Albano, L., et al. (2022). Choice of template delivery mitigates the genotoxic risk and adverse impact of editing in human hematopoietic stem cells. Cell Stem Cell *29*, 1428–1444.e9. https://doi.org/10.1016/j.stem.2022.09.001.

10. Lee, B.C., Gin, A., Wu, C., Singh, K., Grice, M., Mortlock, R., Abraham, D., Fan, X., Zhou, Y., AlJanahi, A., et al. (2024). Impact of CRISPR/HDR editing versus lentiviral transduction on long-term engraftment and clonal dynamics of HSPCs in rhesus macaques. Cell Stem Cell *31*, 788. https://doi.org/10.1016/j.stem.2024.04.005.

11. Schiroli, G., Conti, A., Ferrari, S., Della Volpe, L., Jacob, A., Albano, L., Beretta, S., Calabria, A., Vavassori, V., Gasparini, P., et al. (2019). Precise Gene Editing Preserves Hematopoietic Stem Cell Function following Transient p53-Mediated DNA Damage Response. Cell Stem Cell *24*, 551–565.e8. https://doi.org/10.1016/j.stem.2019.02.019.

12. Baik, R., Cromer, M.K., Glenn, S.E., Vakulskas, C.A., Chmielewski, K.O., Dudek, A.M., Feist, W.N., Klermund, J., Shipp, S., Cathomen, T., et al. (2024). Transient inhibition of 53BP1 increases the frequency of targeted integration in human hematopoietic stem and progenitor cells. Nat. Commun. *15*, 111. https://doi.org/10.1038/s41467-023-43413-w.

13. Conti, A., Giannetti, K., Midena, F., Beretta, S., Gualandi, N., De Marco, R., Carsana, E., Varesi, A., Tavella, T., Alessandrini, L., et al. (2025). Senescence and inflammation are unintended adverse consequences of CRISPR-Cas9/AAV6-mediated gene editing in hematopoietic stem cells. Cell Rep. Med. *6*, 102157. https://doi.org/10.1016/j.xcrm.2025.102157.

14. De Ravin, S.S., Brault, J., Meis, R.J., Liu, S., Li, L., Pavel-Dinu, M., Lazzarotto, C.R., Liu, T., Koontz, S.M., Choi, U., et al. (2021). Enhanced homology-directed repair for highly efficient gene editing in hematopoietic stem/progenitor cells. Blood *137*, 2598–2608. https://doi.org/10.1182/blood.2020008503.

15. Rai, R., Naseem, A., Vetharoy, W., Steinberg, Z., Thrasher, A.J., Santilli, G., and Cavazza, A. (2023). An improved medium formulation for efficient ex vivo gene editing, expansion and engraftment of hematopoietic stem and progenitor cells. Mol. Ther. Methods Clin. Dev. *29*, 58–69. https://doi.org/10.1016/j.omtm.2023.02.014.

16. Blattner, G., Cavazza, A., Thrasher, A.J., and Turchiano, G. (2020). Gene Editing and Genotoxicity: Targeting the Off-Targets. Front Genome *2*, 613252. https://doi.org/10.3389/fgeed.2020.613252.

17. Jeffers, M.S., Xi, C.E., Bapuji, R., Wotherspoon, H., Kimmelman, J., Bedford, P., McIsaac, D.I., Lalu, M.M., and Fergusson, D.A. (2024). Synthesizing regulatory guidance for demonstrating preclinical efficacy and translating promising cell therapies to early phase clinical trials: a scoping review. BMC Med. *22*, 487. https://doi.org/10.1186/s12916-024-03690-8.

18. Castanza, A.S., Recla, J.M., Eby, D., Thorvaldsdóttir, H., Bult, C.J., and Mesirov, J.P. (2023). Extending support for mouse data in the Molecular Signatures Database (MSigDB). Nat. Methods *20*, 1619–1620. https://doi.org/10.1038/s41592-023-02014-7.

19. Ferrari, S., Jacob, A., Beretta, S., Unali, G., Albano, L., Vavassori, V., Cittaro, D., Lazarevic, D., Brombin, C., Cugnata, F., et al. (2020). Efficient gene editing of human long-term hematopoietic stem cells validated by clonal tracking. Nat. Biotechnol. *38*, 1298–1308. https://doi.org/10.1038/s41587-020-0551-y.

20. Canny, M.D., Moatti, N., Wan, L.C.K., Fradet-Turcotte, A., Krasner, D., Mateos-Gomez, P.A., Zimmermann, M., Orthwein, A., Juang, Y.C., Zhang, W., et al. (2018). Inhibition of 53BP1 favors homology-dependent DNA repair and increases CRISPR-Cas9 genome-editing efficiency. Nat. Biotechnol. *36*, 95–102. https://doi.org/10.1038/nbt.4021.

21. Whittaker, T.E., Moula, S.E., Bahal, S., Bakri, F.G., Hayajneh, W.A., Daoud, A.K., Naseem, A., Cavazza, A., Thrasher, A.J., and Santilli, G. (2024). Multidimensional Response Surface Methodology for the Development of a Gene Editing Protocol for p67(phox)-Deficient Chronic Granulomatous Disease. Hum. Gene Ther. *35*, 298–312. https://doi.org/10.1089/hum.2023.114.

22. Lee, B.C., Lozano, R.J., and Dunbar, C.E. (2021). Understanding and overcoming adverse consequences of genome editing on hematopoietic stem and progenitor cells. Mol. Ther. *29*, 3205–3218. https://doi.org/10.1016/j.ymthe.2021.09.001.

23. Turchiano, G., Andrieux, G., Klermund, J., Blattner, G., Pennucci, V., El Gaz, M., Monaco, G., Poddar, S., Mussolino, C., Cornu, T.I., et al. (2021). Quantitative evaluation of chromosomal rearrangements in gene-edited human stem cells by CAST-Seq. Cell Stem Cell *28*, 1136–1147.e5. https://doi.org/10.1016/j.stem.2021.02.002.

24. Flach, J., Bakker, S.T., Mohrin, M., Conroy, P.C., Pietras, E.M., Reynaud, D., Alvarez, S., Diolaiti, M.E., Ugarte, F., Forsberg, E.C., et al. (2014). Replication stress is a potent driver of functional decline in ageing haematopoietic stem cells. Nature *512*, 198–202. https://doi.org/10.1038/nature13619.

25. Mehta, A., and Haber, J.E. (2014). Sources of DNA double-strand breaks and models of recombinational DNA repair. Cold Spring Harb. Perspect. Biol. *6*, a016428. https://doi.org/10.1101/cshperspect.a016428.

26. Calabria, A., Cipriani, C., Spinozzi, G., Rudilosso, L., Esposito, S., Benedicenti, F., Albertini, A., Pouzolles, M., Luoni, M., Giannelli, S., et al. (2023). Intrathymic AAV delivery results in therapeutic site-specific integration at TCR loci in mice. Blood *141*, 2316–2329. https://doi.org/10.1182/blood.2022017378.

27. Sabatino, D.E., Bushman, F.D., Chandler, R.J., Crystal, R.G., Davidson, B.L., Dolmetsch, R., Eggan, K.C., Gao, G., Gil-Farina, I., Kay, M.A., et al. (2022). Evaluating the state of the science for adeno-associated virus integration: An integrated perspective. Mol. Ther. *30*, 2646–2663. https://doi.org/10.1016/j.ymthe.2022.06.004.

28. Lattanzi, A., Camarena, J., Lahiri, P., Segal, H., Srifa, W., Vakulskas, C.A., Frock, R.L., Kenrick, J., Lee, C., Talbott, N., et al. (2021). Development of beta-globin gene correction in human hematopoietic stem cells as a potential durable treatment for sickle cell disease. Sci. Transl. Med. *13*, eabf2444. https://doi.org/10.1126/scitranslmed.abf2444.

29. Maganti, H.B., Bailey, A.J.M., Kirkham, A.M., Shorr, R., Pineault, N., and Allan, D.S. (2021). Persistence of CRISPR/Cas9 gene edited hematopoietic stem cells following transplantation: A systematic review and meta-analysis of preclinical studies. Stem Cells Transl. Med. *10*, 996–1007. https://doi.org/10.1002/sctm.20-0520.

30. Carreras, E. (2019). In In the EBMT Handbook: Hematopoietic Stem Cell Transplantation and Cellular Therapies, E. Carreras, C. Dufour, M. Mohty, and N. Kroger, eds. (Springer). https://doi.org/10.1007/978-3-030-02278-5.

31. Scheid, C., Draube, A., Reiser, M., Schulz, A., Chemnitz, J., Nelles, S., Fuchs, M., Winter, S., Wickramanayake, P.D., Diehl, V., and Söhngen, D. (1999). Using at least 5x10(6)/kg CD34+ cells for autologous stem cell transplantation significantly reduces febrile complications and use of antibiotics after transplantation. Bone Marrow Transplant. *23*, 1177–1181. https://doi.org/10.1038/sj.bmt.1701748.

32. Tucci, F., Frittoli, M., Barzaghi, F., Calbi, V., Migliavacca, M., Ferrua, F., Fumagalli, F., Lorioli, L., Castagnaro, L., Facchini, M., et al. (2019). Bone marrow harvesting from paediatric patients undergoing haematopoietic stem cell gene therapy. Bone Marrow Transplant. *54*, 1995–2003. https://doi.org/10.1038/s41409-019-0573-6.

33. Schmiderer, L., Subramaniam, A., Žemaitis, K., Bäckström, A., Yudovich, D., Soboleva, S., Galeev, R., Prinz, C.N., Larsson, J., and Hjort, M. (2020). Efficient and nontoxic biomolecule delivery to primary human hematopoietic stem cells using nanostraws. Proc. Natl. Acad. Sci. USA *117*, 21267–21273. https://doi.org/10.1073/pnas.2001367117.

34. Vavassori, V., Ferrari, S., Beretta, S., Asperti, C., Albano, L., Annoni, A., Gaddoni, C., Varesi, A., Soldi, M., Cuomo, A., et al. (2023). Lipid nanoparticles allow efficient and harmless ex vivo gene editing of human hematopoietic cells. Blood *142*, 812–826. https://doi.org/10.1182/blood.2022019333.

35. Boutin, J., Cappellen, D., Rosier, J., Amintas, S., Dabernat, S., Bedel, A., and Moreau-Gaudry, F. (2022). ON-Target Adverse Events of CRISPR-Cas9 Nuclease: More Chaotic than Expected. CRISPR J. *5*, 19–30. https://doi.org/10.1089/crispr.2021.0120.

36. Lopes, R., and Prasad, M.K. (2024). Beyond the promise: evaluating and mitigating off-target effects in CRISPR gene editing for safer therapeutics. Front. Bioeng. Biotechnol. *11*, 1339189. https://doi.org/10.3389/fbioe.2023.1339189.

37. Cromer, M.K., Majeti, K.R., Rettig, G.R., Murugan, K., Kurgan, G.L., Bode, N.M., Hampton, J.P., Vakulskas, C.A., Behlke, M.A., and Porteus, M.H. (2023). Comparative analysis of CRISPR off-target discovery tools following ex vivo editing of CD34(+) hematopoietic stem and progenitor cells. Mol. Ther. *31*, 1074–1087. https://doi.org/10.1016/j.ymthe.2023.02.011.

38. Kerwash, E., Sajic, M., Rantell, K.R., McBlane, J.W., Johnston, J.D., Niewiarowska, A., Butler, A.S., and Cole, S. (2024). Regulatory Assessment of Casgevy for the Treatment of Transfusion-Dependent beta-Thalassemia and Sickle Cell Disease with Recurrent Vaso-Occlusive Crises. Curr. Issues Mol. Biol. *46*, 8209–8225. https://doi.org/10.3390/cimb46080485.

39. Cancellieri, S., Zeng, J., Lin, L.Y., Tognon, M., Nguyen, M.A., Lin, J., Bombieri, N., Maitland, S.A., Ciuculescu, M.F., Katta, V., et al. (2023). Human genetic diversity alters off-target outcomes of therapeutic gene editing. Nat. Genet. *55*, 34–43. https://doi.org/10.1038/s41588-022-01257-y.

40. Scott, D.A., and Zhang, F. (2017). Implications of human genetic variation in CRISPR-based therapeutic genome editing. Nat. Med. *23*, 1095–1101. https://doi.org/10.1038/nm.4377.

41. Verkuijl, S.A., and Rots, M.G. (2019). The influence of eukaryotic chromatin state on CRISPR-Cas9 editing efficiencies. Curr. Opin. Biotechnol. *55*, 68–73. https://doi.org/10.1016/j.copbio.2018.07.005.

42. Smith, R.H., Chen, Y.C., Seifuddin, F., Hupalo, D., Alba, C., Reger, R., Tian, X., Araki, D., Dalgard, C.L., Childs, R.W., et al. (2020). Genome-Wide Analysis of Off-Target CRISPR/Cas9 Activity in Single-Cell-Derived Human Hematopoietic Stem and Progenitor Cell Clones. Genes *11*, 1501. https://doi.org/10.3390/genes11121501.

43. Araki, D., Chen, V., Redekar, N., Salisbury-Ruf, C., Luo, Y., Liu, P., Li, Y., Smith, R.H., Dagur, P., Combs, C., and Larochelle, A. (2025). Post-transplant G-CSF impedes engraftment of gene-edited human hematopoietic stem cells by exacerbating p53-mediated DNA damage response. Cell Stem Cell *32*, 53–70.e8. https://doi.org/10.1016/j.stem.2024.10.013.

44. Martin, M. (2011). Cutadapt Removes Adapter Sequences From High-Throughput Sequencing Reads. EMBnet J. *17*, 10–12. https://doi.org/10.14806/ej.17.1.200.

45. Clement, K., Rees, H., Canver, M.C., Gehrke, J.M., Farouni, R., Hsu, J.Y., Cole, M.A., Liu, D.R., Joung, J.K., Bauer, D.E., and Pinello, L. (2019). CRISPResso2 provides accurate and rapid genome editing sequence analysis. Nat. Biotechnol. *37*, 224–226. https://doi.org/10.1038/s41587-019-0032-3.

46. Kolde, R.e.a. (2019). Pheatmap: pretty heatmaps. R package version *1*, 726.

47. Cesana, D., Calabria, A., Rudilosso, L., Gallina, P., Benedicenti, F., Spinozzi, G., Schiroli, G., Magnani, A., Acquati, S., Fumagalli, F., et al. (2021). Retrieval of vector integration sites from cell-free DNA. Nat. Med. *27*, 1458–1470. https://doi.org/10.1038/s41591-021-01389-4.

48. Gentner, B., Tucci, F., Galimberti, S., Fumagalli, F., De Pellegrin, M., Silvani, P., Camesasca, C., Pontesilli, S., Darin, S., Ciotti, F., et al. (2021). Hematopoietic Stem- and Progenitor-Cell Gene Therapy for Hurler Syndrome. N. Engl. J. Med. *385*, 1929–1940. https://doi.org/10.1056/NEJMoa2106596.

49. Carlo Cipriani, L.R., Dalwadi, D.A., Masseroli, M., Grompe, M., Montini, E., Calabria, A., and Cesana, D. (2025). RAAVioli: A comprehensive approach to characterizing AAV vector integrations and rearrangements. Mol Ther Adv. *34*, 201659. https://www.cell.com/molecular-therapy-family/advances/fulltext/S3117-387X(25)00009-6.

OMTA, Volume *34*

Supplemental information

In-depth characterization of stem cell potency and genotoxicity for clinical-scale *ex vivo* CRISPR-Cas9 gene editing

Asma Naseem, Winston Vetharoy, Thomas E. Whittaker, Federica Zinghirino, Antonio Carusillo, Roopkatha Ghosh, Eduardo Gomez-Castaneda, Hashim Ali, Carlo Cipriani, Eugenio Montini, Adrian J. Thrasher, Giorgia Santilli, Daniela Cesana, Giandomenico Turchiano, and Alessia Cavazza

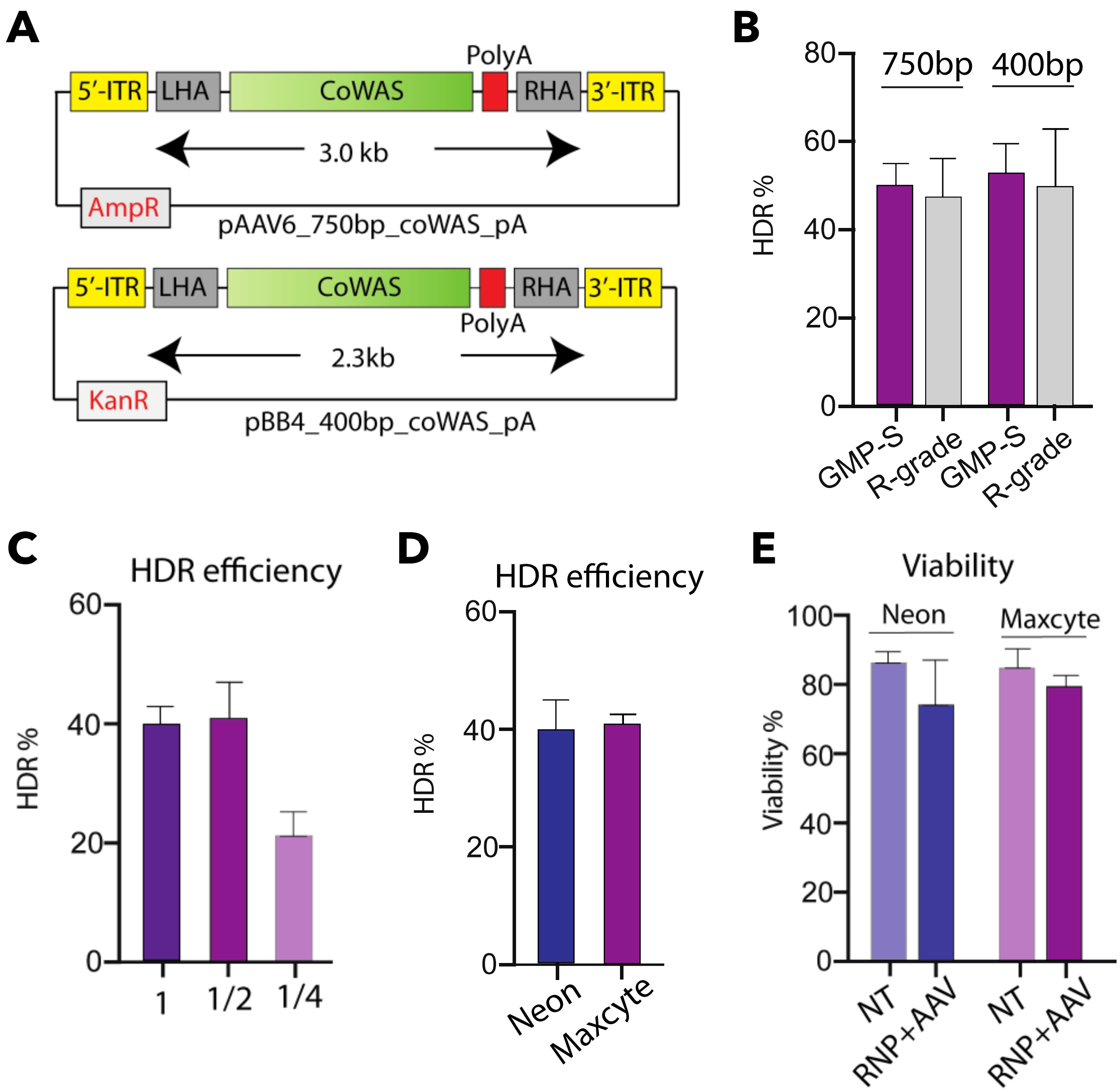

Figure S1. HSPC gene editing scale-up protocol and reagents optimization.
(A) maps of the AAV donor molecules used in the proof-of concept study by Rai et al[1] (pAAV6_750bp_coWAS_pA) and in this study (pBB4_400pb_coWAS_pA). (B) Frequency of coWAS cassette knock-in via HDR in HSPCs (n=3 biological replicates) when using the two different AAV donor vectors (as in (A)) and cGMP- or research-grade reagents. (C) Frequency of coWAS cassette knock-in via HDR in HSPCs (n=3 biological replicates) when using different RNP doses. (D-E) Frequency of coWAS cassette knock-in via HDR in HSPCs (D) and cell viability (E) when electroporating cells with different electroporation machines (n=3 biological replicates). Unmanipulated HSPCs were used as control. Data are presented as mean ± SD.

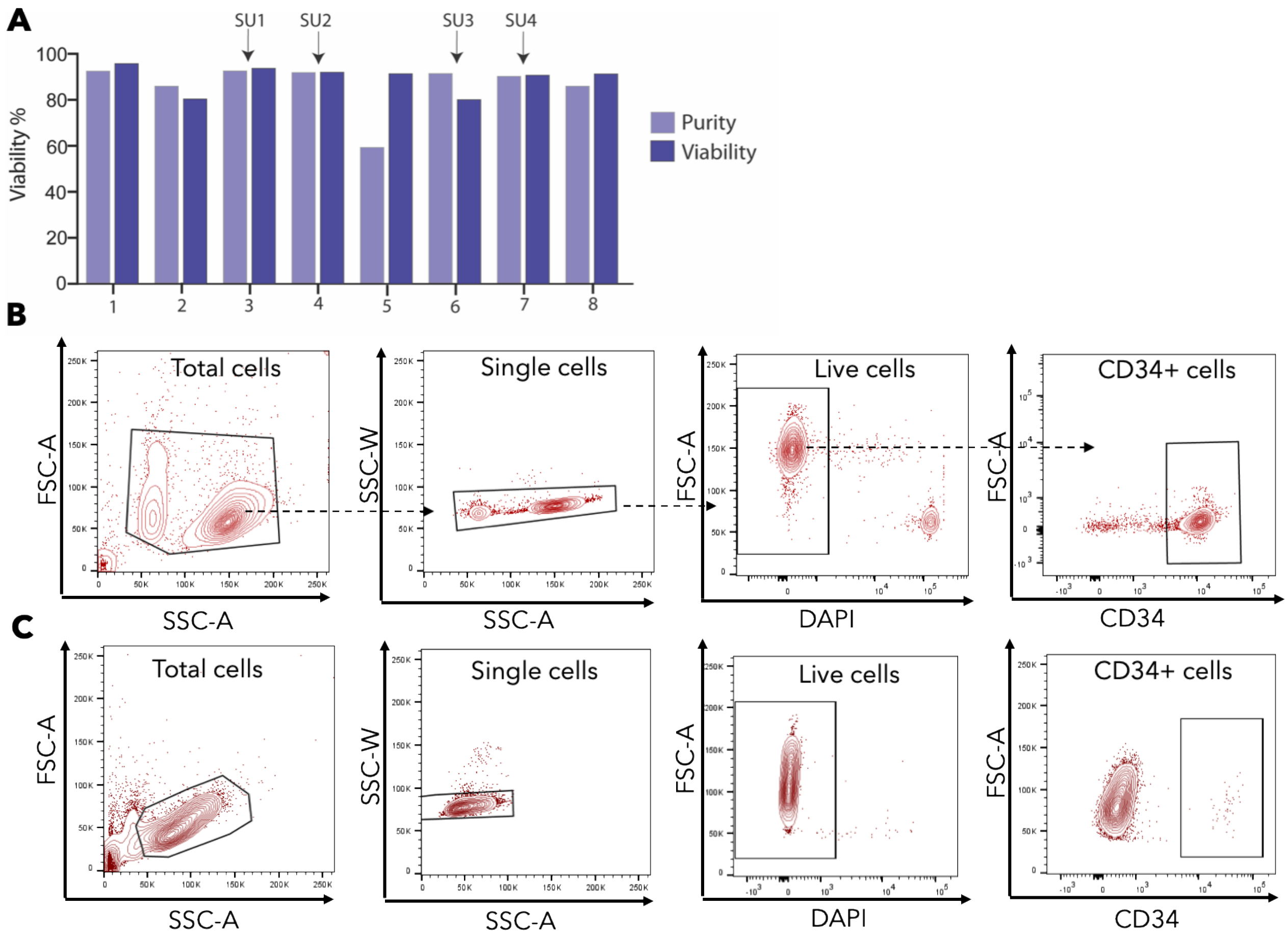

Figure S2: Purity and viability of HSPCs isolated from leukapheresis products (A) CD34+ isolation was carried out from G-CSF mobilised leukapheresis from eight healthy donors. Purity (fraction of CD34+ cells) and viability post selection is shown. Arrows indicate the donors used for the 4 scale ups in this study. (B) Gating strategy used to assess HSPC purity (%CD34+) and viability (DAPI-) in HSPC samples and (C) in unstained controls.

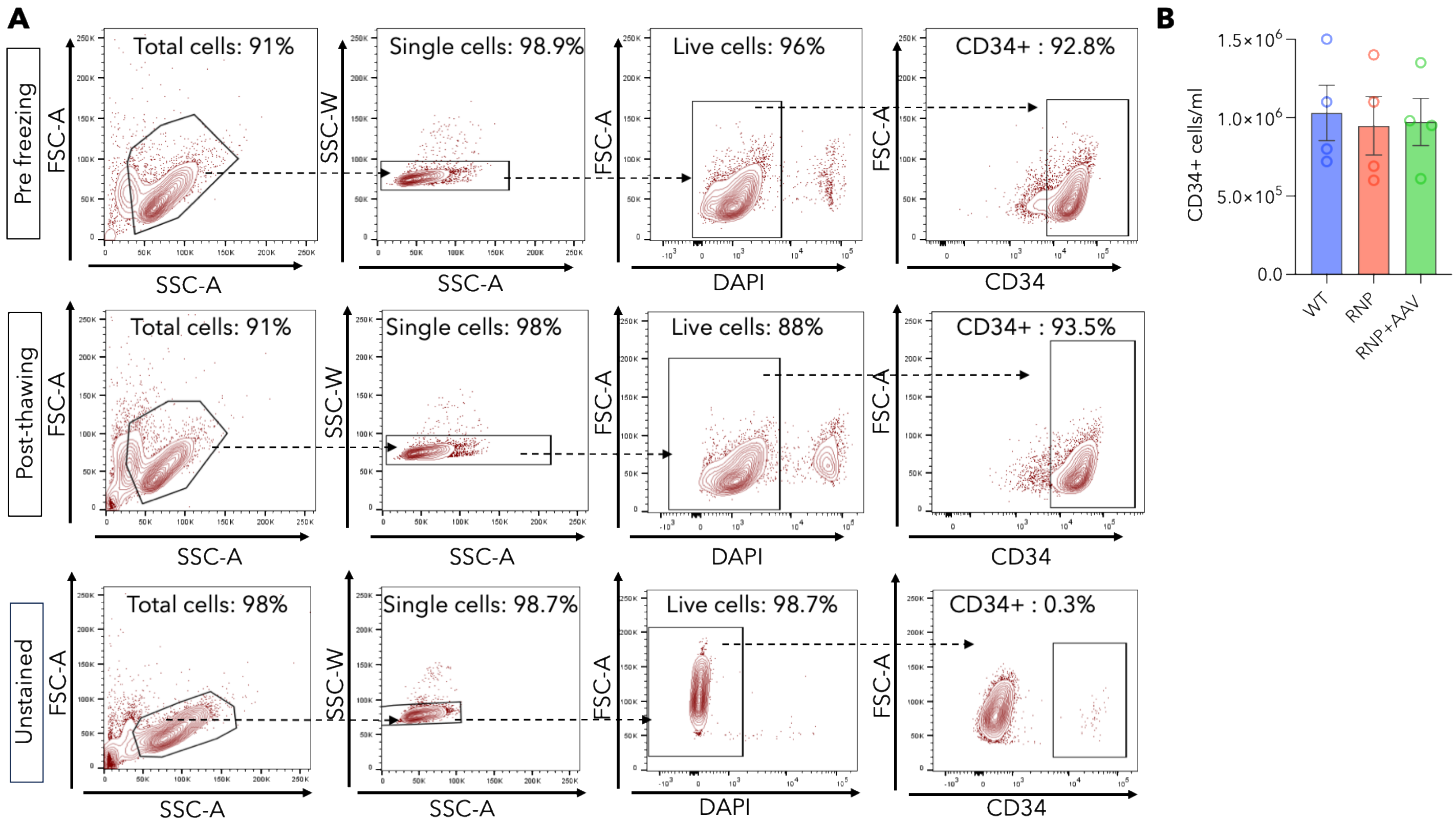

Figure S3: Gating strategy to assess HSPC purity and viability pre- and post-cryopreservation (A) CD34+ cell isolation was carried out from G-CSF mobilised leukapheresis from eight healthy donors. Purity (fraction of CD34+ cells) and viability post selection is shown. Arrows indicate the donors used for the 4 scale ups in this study. (B) Number of CD34+ HSPCs recovered after thawing of the cryopreserved products (number of cells per ml, in 10 ml total; n=4, where each dot represents one donor source). Data are presented as mean ± SD.

Number of mice analysed				
	**SU-1**	**SU-2**	**SU-3**	**SU-4**
**WT**	5	4	2	2
**RNP**	4	4	3	2
**RNP+AAV**	15	16	9	3

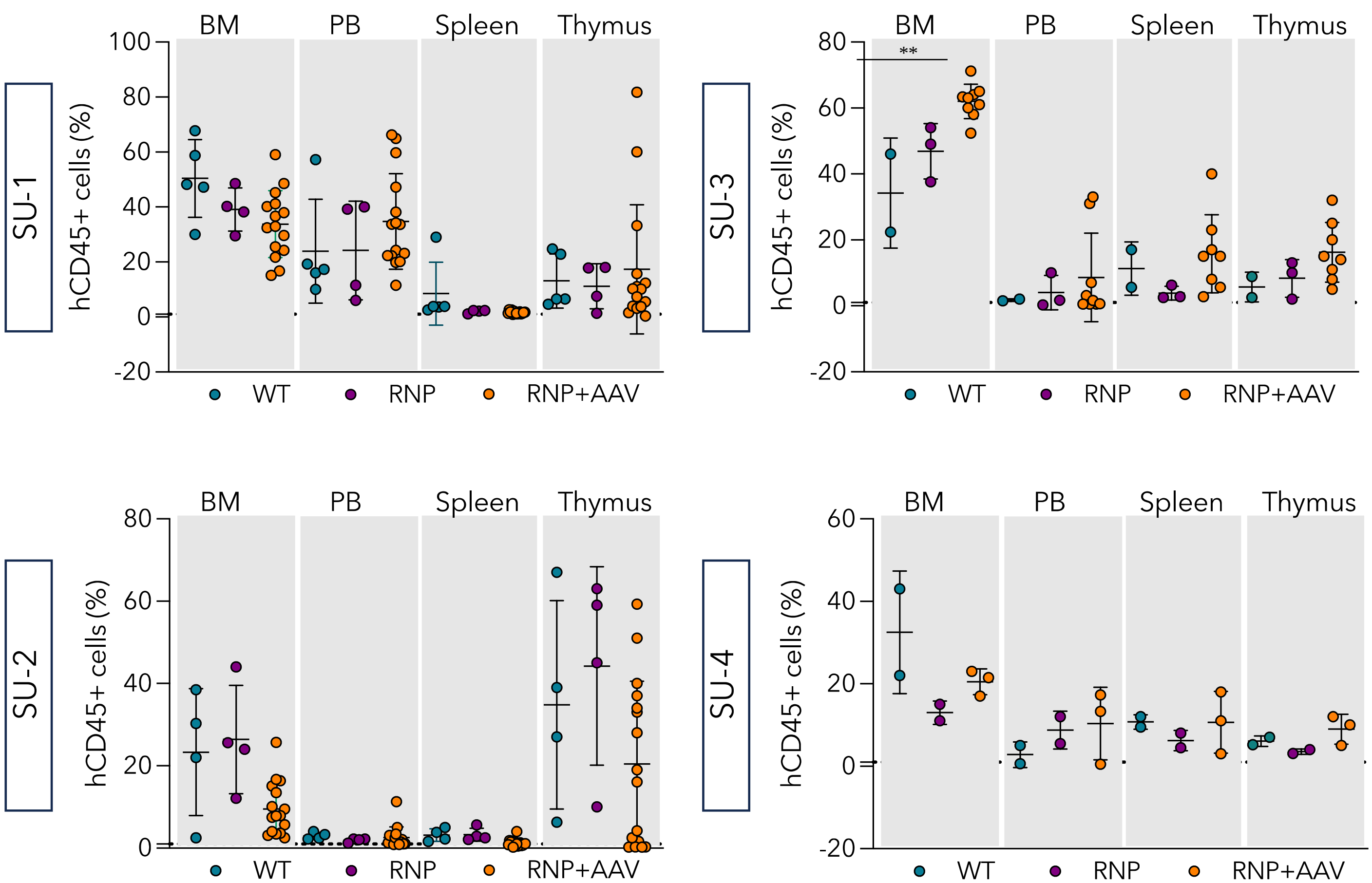

Figure S4: Assessment of human chimerism in xenotransplanted NSG mice. Engraftment frequencies in the bone marrow (BM), peripheral blood (PB), spleen and thymus of transplanted NSG mice at 14 weeks post injection, expressed as the frequency of CD45+ cells detected by flow cytometry, per each single SU product infused

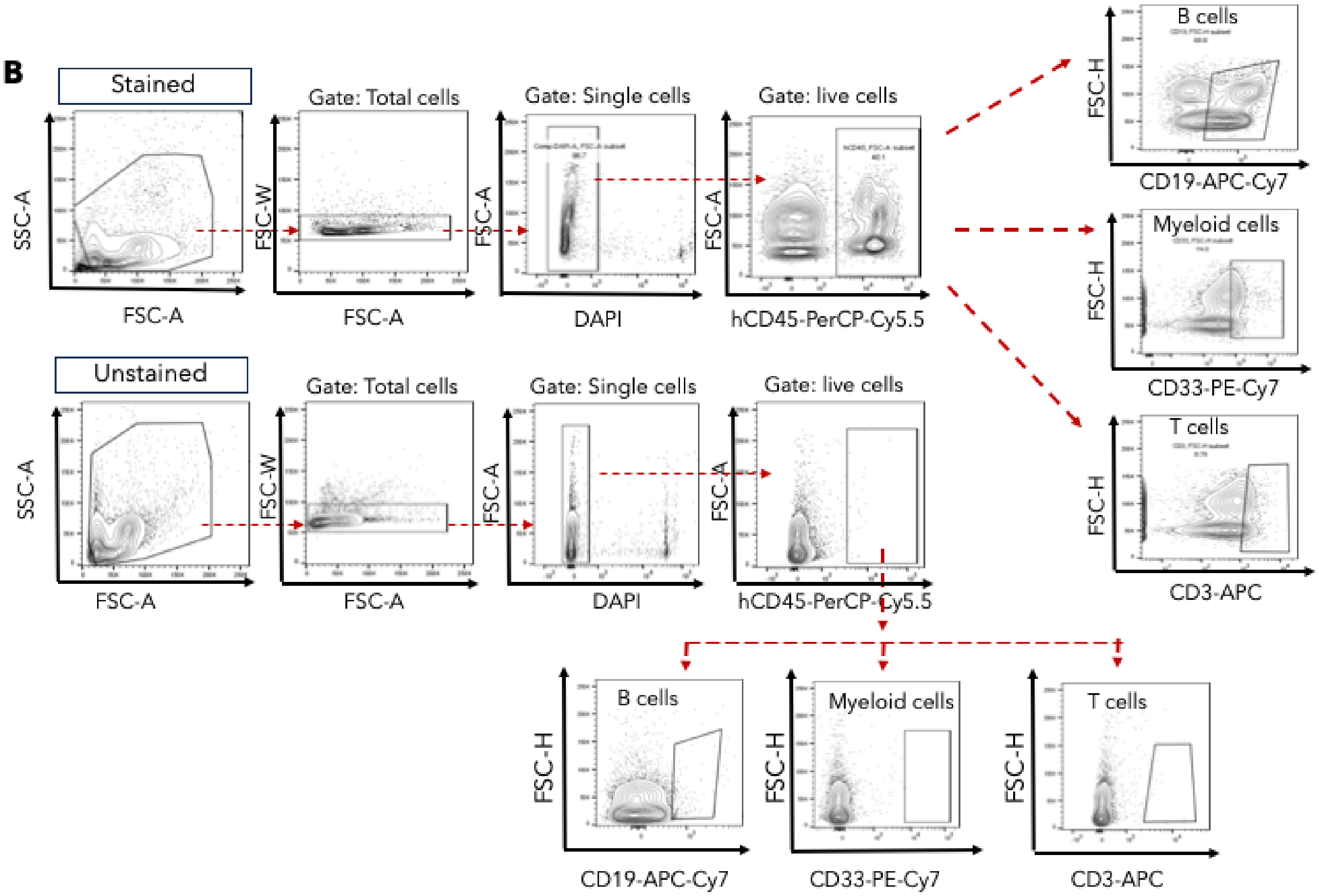

Figure S5: Assessment of human chimerism in xenotransplanted NSG mice. **(A)** Representative gating strategy for the for the analysis of hematopoietic subpopulations in the hematopoietic organs of transplanted mice.

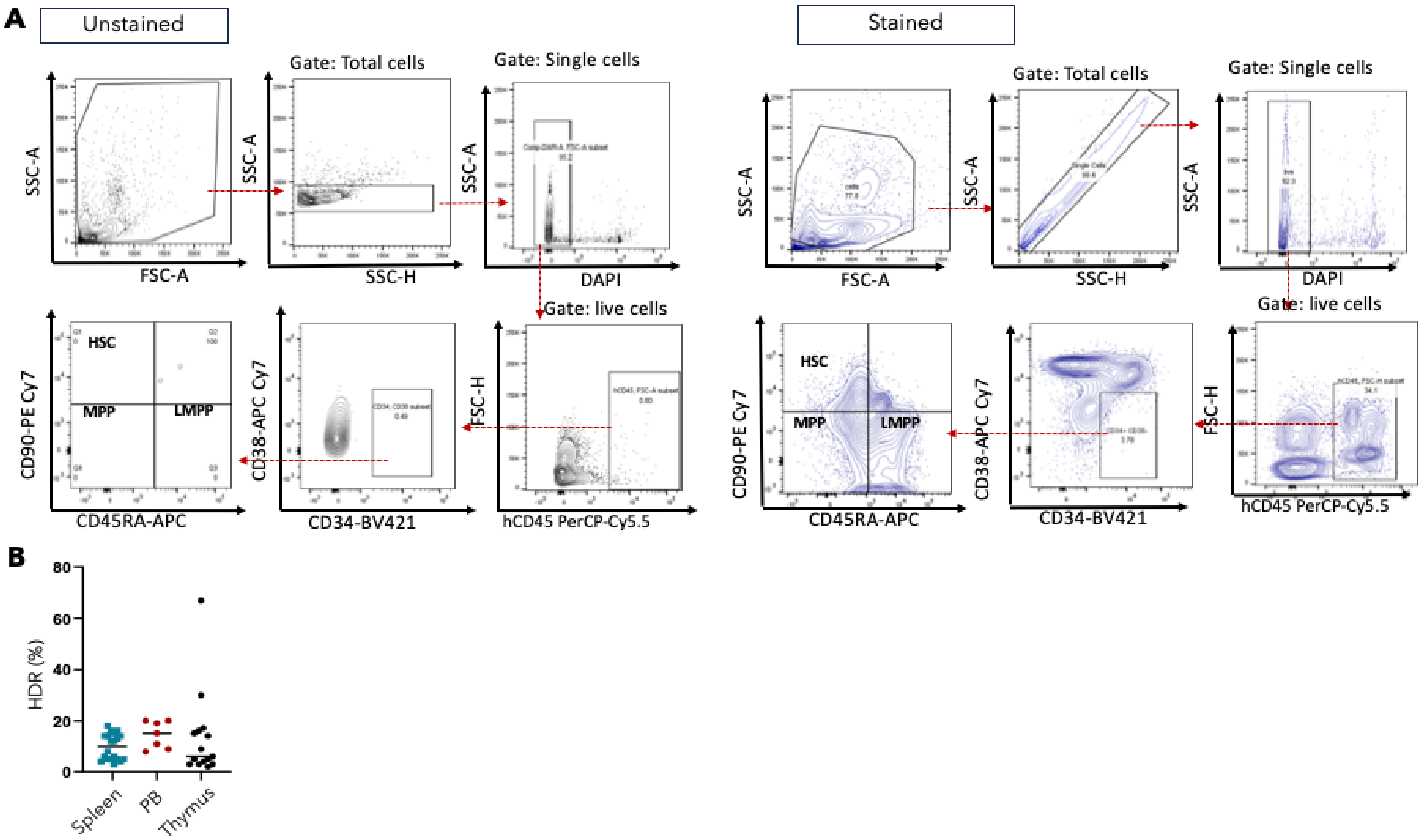

Figure S6: Assessment of human chimerism in xenotransplanted NSG mice. (**A)** Representative gating strategy for the for the analysis of HSPC subpopulations in the bone marrow of transplanted mice. Human cells (hCD45+) were isolated from the BM of mice 14 weeks post injections. hCD45+ population was phenotyped into primitive HSCs (CD34+ CD38- CD90+ CD45RA-), MPPs (CD34+ CD38- CD90- CD45RA-) and CD38+ committed progenitors (CD34+ CD38+). (**B)** Frequency of coWAS cassette integration via HDR in the PB, spleen and thymus of mice transplanted with SU-1 RNP+AAV HSPCs, as detected by ddPCR. Each dot represents a mouse.

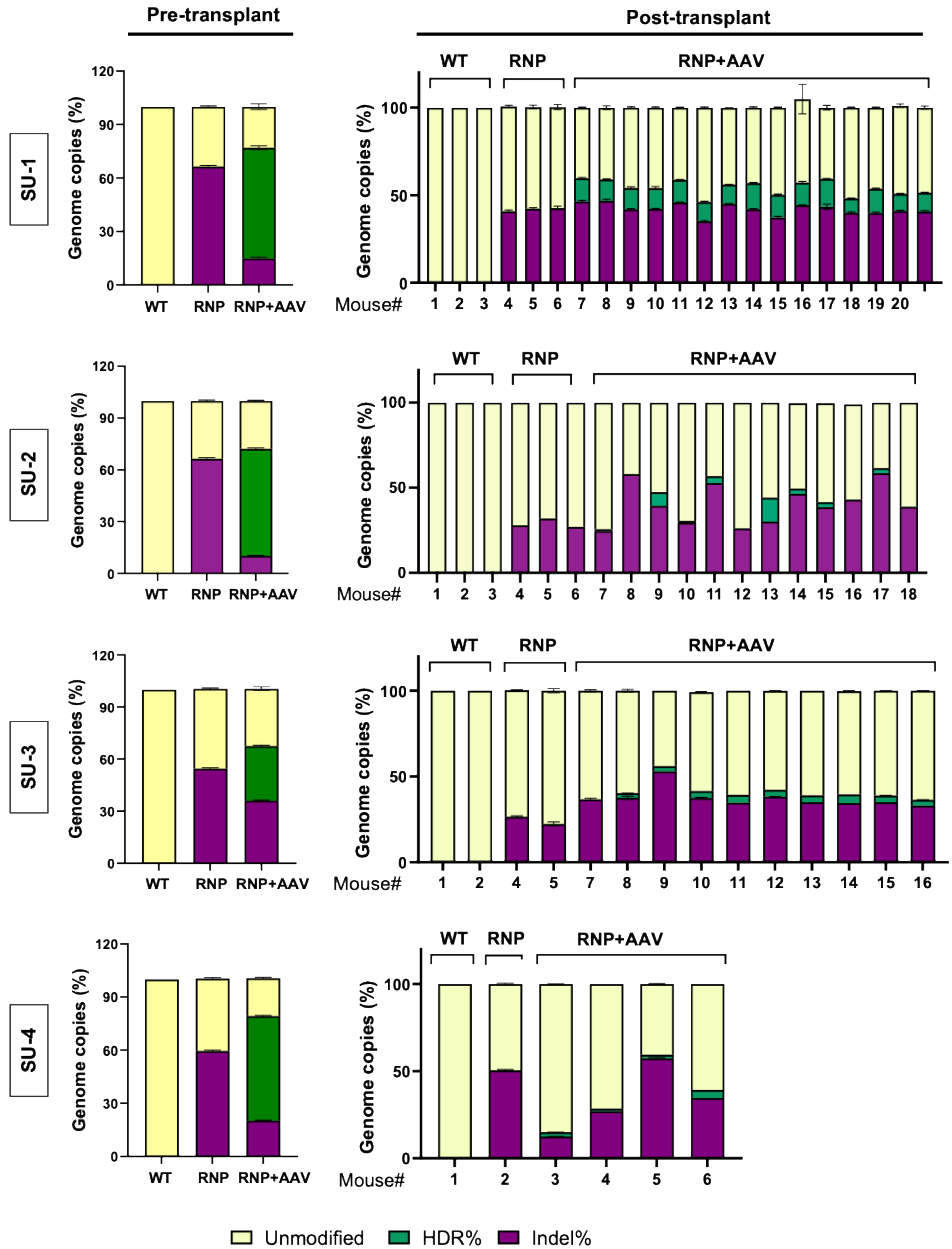

Figure S7: Assessment of gene editing frequency pre and post-transplantation into NSG mice. Fraction of cell genomes with a *WAS* locus unmodified, bearing indels (Indel%) or with coWAS cassette integration (HDR%) in each ex vivo HSPC scale-up product (pre-transplantation) and in CD45+ cells retrieved from the bone marrow of mice transplanted with each of the HSPC product indicated (post-transplantation) as detected by ddPCR. Data are presented as mean ± SD, where n=3 technical ddPCR replicates.

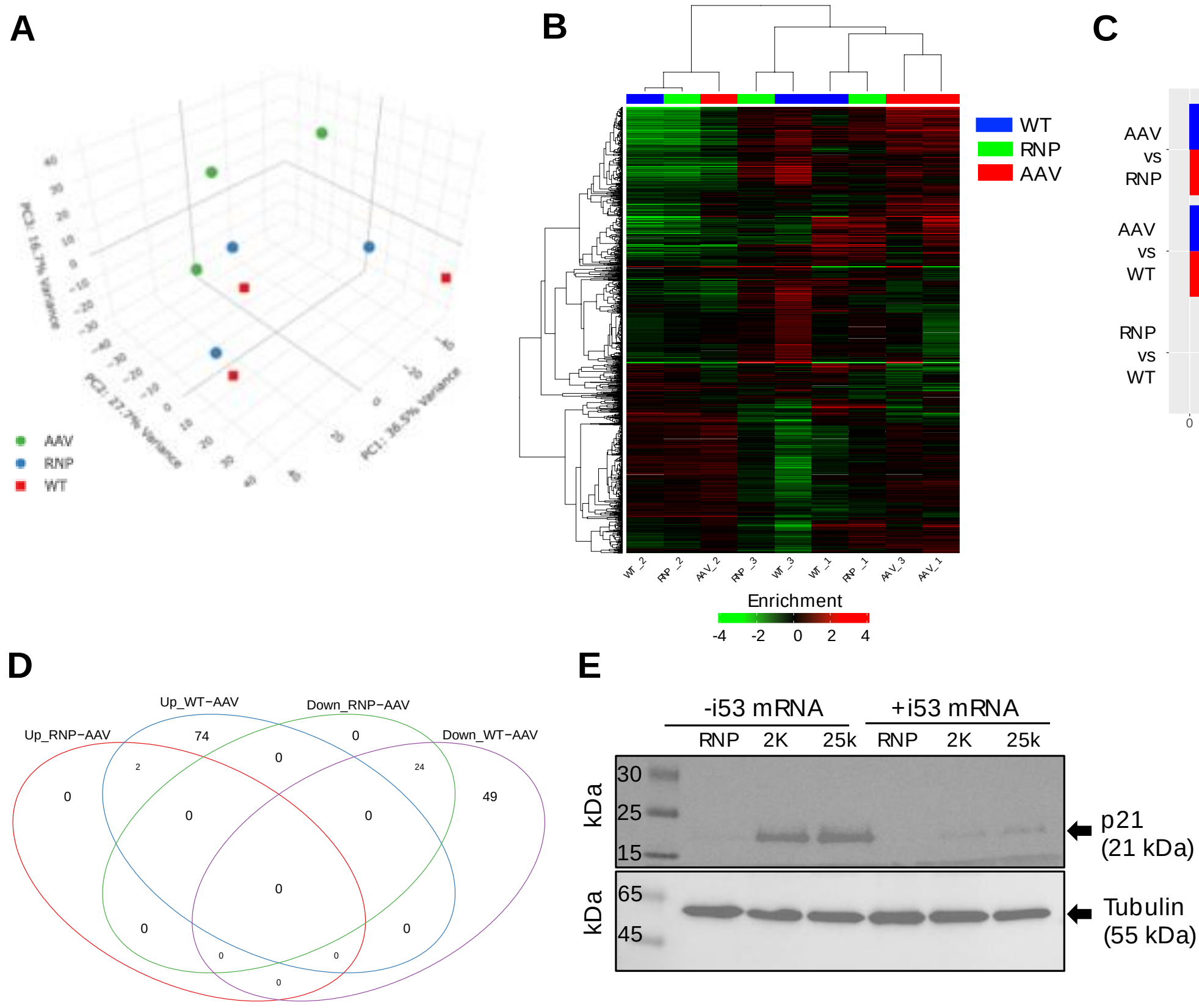

Figure S8: Dampening of p53 signalling improves engraftment of HSPCs that underwent HDR.
(A) Principal Component Analysis conducted on the whole RNA-seq data retrieved from the 3 experimental groups (n=3 biological replicates each). (B) Unsupervised hierarchical clustering of RNA-seq data. (C) Number of genes down- or up-regulated for each pairwise comparison. (D) Venn diagrams displaying the number of differentially expressed genes for each pairwise comparison (RNP vs RNP+AAV or WT vs RNP+AAV) and overlaps among groups. (E) Representative Western blot of p21 expression in HSPCs gene edited using different AAV MOIs and with or without the addition of i53 mRNA. Tubulin was used as a reference for protein quantification.

A

Mouse#	SU1		SU2		SU3		SU4	
	BM smear	Spleen	BM smear	Spleen	BM smear	Spleen	BM smear	Spleen
WT	WNL	EMH	WNL	EMH	WNL	EMH	WNL	EMH
WT	WNL	EMH	WNL	EMH	WNL	EMH	WNL	EMH
WT	WNL	EMH	WNL	EMH	WNL	EMH	WNL	EMH
WT	WNL	EMH	WNL	EMH				
WT	WNL	EMH						
RNP+AAV	WNL	EMH	WNL	EMH	WNL	EMH	WNL	EMH
RNP+AAV	WNL	EMH	WNL	EMH	WNL	EMH	WNL	EMH
RNP+AAV	WNL	EMH	WNL	EMH	WNL	EMH	WNL	EMH
RNP+AAV	WNL	EMH	WNL	EMH	WNL	EMH	WNL	EMH
RNP+AAV	WNL	EMH	WNL	EMH				
RNP+AAV	WNL	EMH	WNL	EMH				
RNP+AAV	WNL	EMH	WNL	EMH				
RNP+AAV	WNL	EMH	WNL	EMH				
RNP+AAV	WNL	EMH	WNL	EMH				
RNP+AAV	WNL	EMH	WNL	EMH				
RNP+AAV	WNL	EMH	WNL	EMH				
RNP+AAV	WNL	EMH	WNL	EMH				
RNP+AAV	WNL	EMH	WNL	EMH				
RNP+AAV	WNL	EMH	WNL	EMH				
RNP+AAV	WNL	EMH	WNL	EMH				
	normal	minimal	mild	moderate	severe			

B

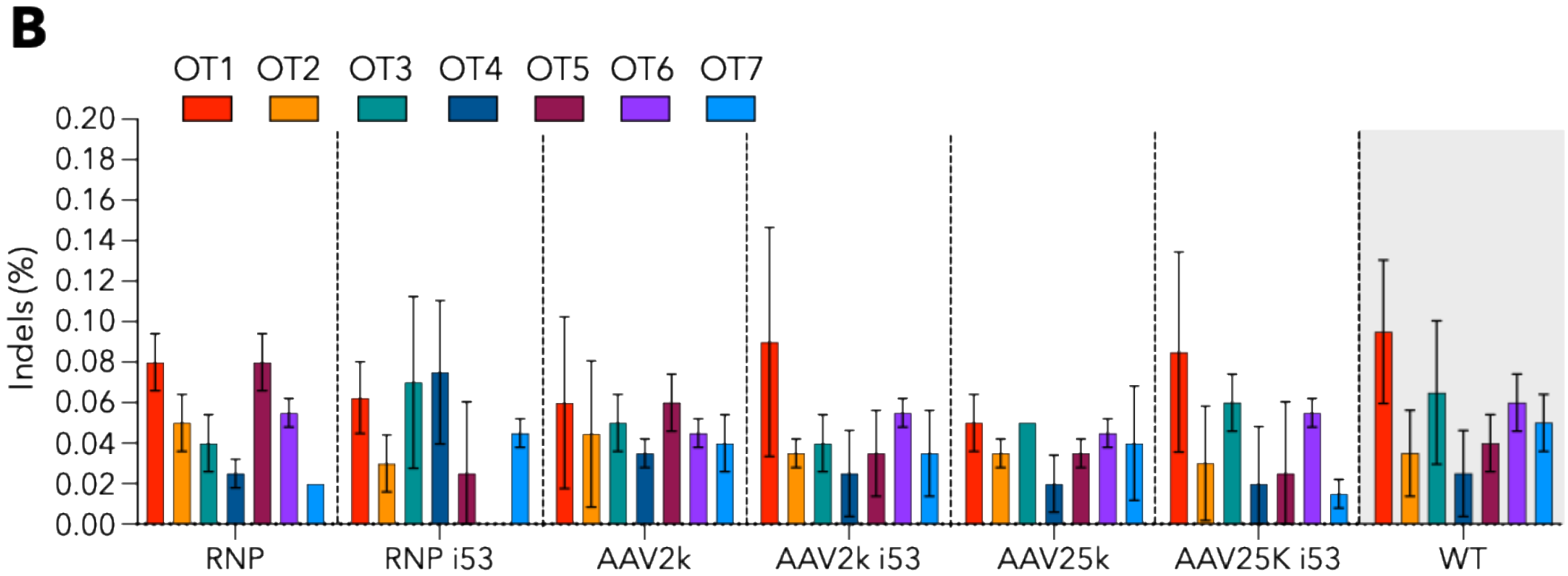

C

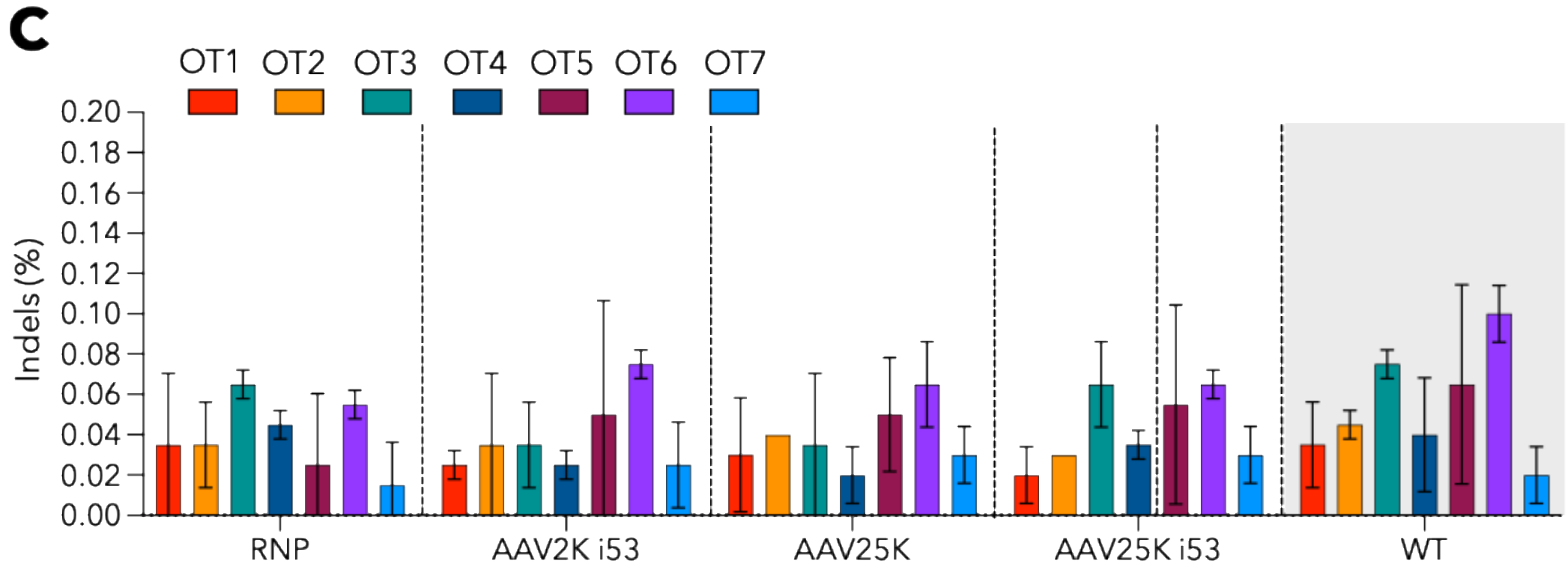

Figure S9. Evaluating the genotoxicity of the gene editing platform in vitro and in vivo. (A) Table summarising histopathological results obtained by analysing BM smears and spleen sections upon H&E staining. BM smears revealed expected morphology and normal levels of precursor cells among the groups. Minimal to mild extra medullary haematopoiesis (EMH) was observed in spleen sections, comparable among the groups.

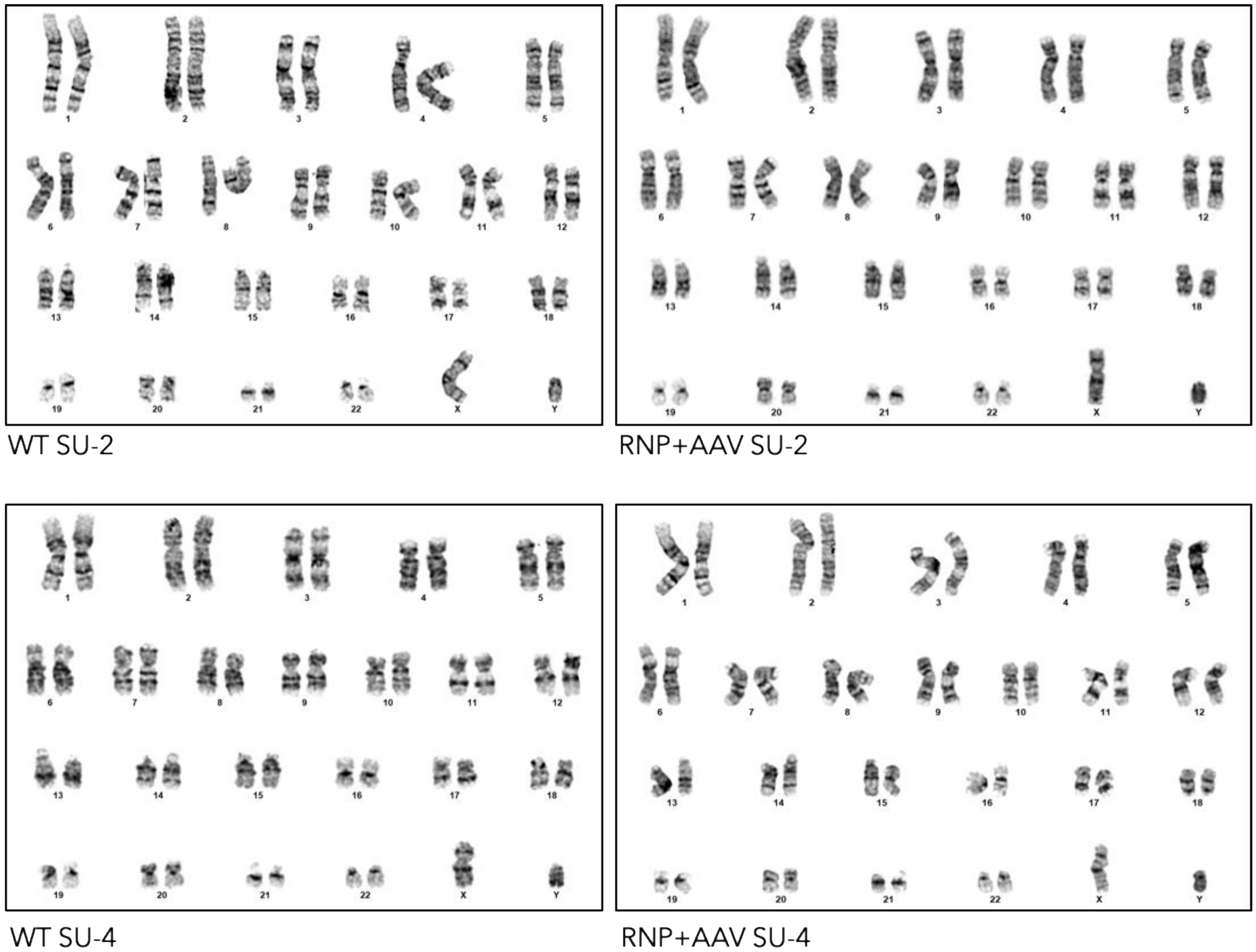

Figure S10. Evaluating the genotoxicity of the gene editing platform in vitro. Karyotypes of edited (RNP+AAV) and unedited (WT) HSPCs from individual scale up experiments.

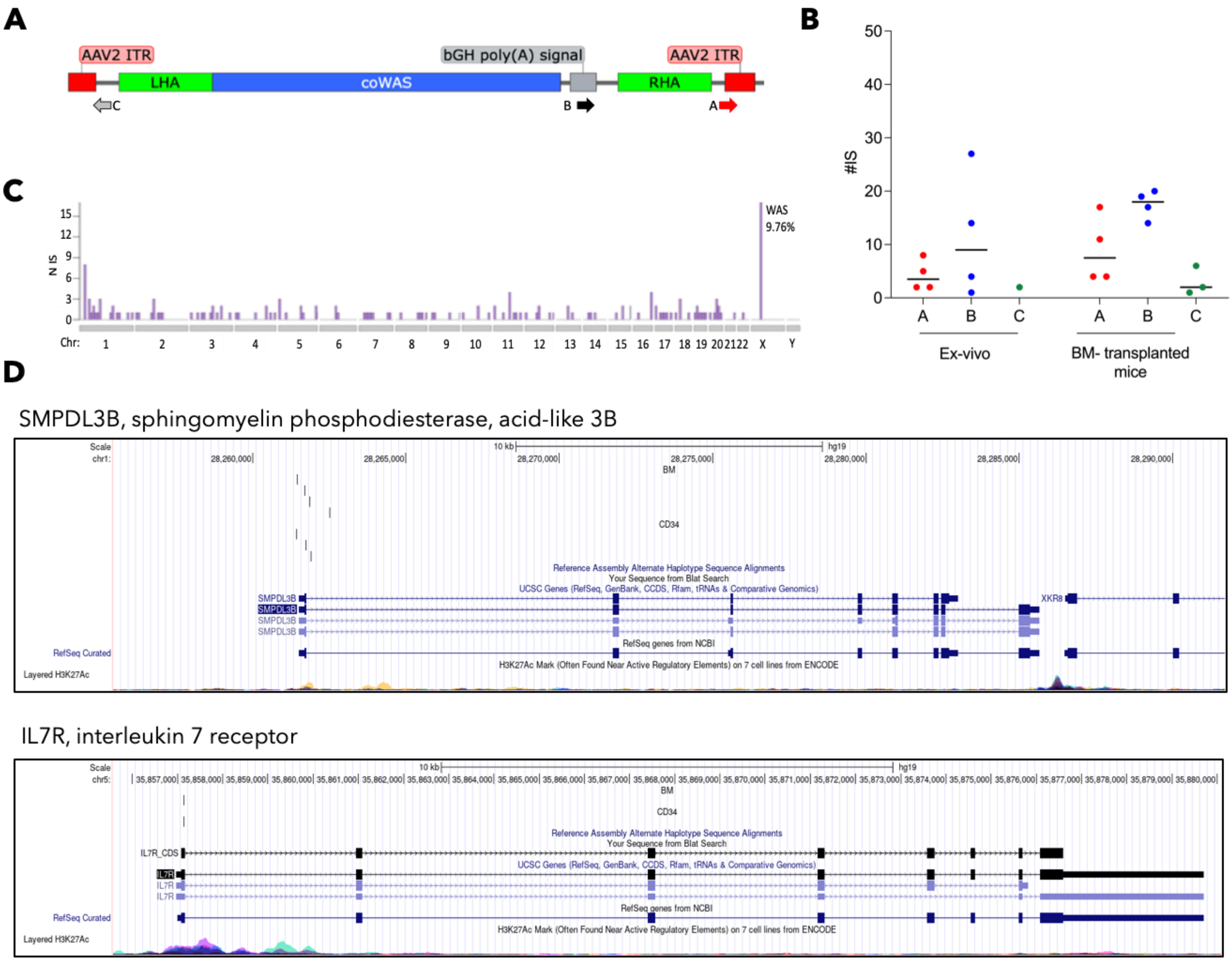

Figure S11. Analysis of AAV donor molecule integration in gene edited HSPCs.
(A) Schematic representation of the strategy used to map AAV integrations involving different features of the AAV donor vector. (B) Number of AAV integration sites (IS) per each sample analysed, ex vivo and in vivo. (C) Distribution of AAV integrations across chromosomes. High IS integration frequency was observed in chromosome X where the *WAS* locus is located. (D) Representative UCSC Genome Browser snapshot of two genes hit by AAV integration retrieved from edited HSPCs (CD34 track) or from CD45+ cells isolated from the BM of transplanted mice (BM track).

Table S1. Genomic sites analysed by amplicon deep-sequencing to assess off-target editing.

Deetction method	Site name	BED Chromosome	Off-Target start	Off-Target end	Strand	Off-Target Sequence	Mismatches
Guide-seq	OT-2	chr1	50322318	50322341	+	GGAAACAG CACCCTGA GAGGAGG	5
	OT-3	chr2	112824051	112824074	-	GCAGCATG AACAATGG GTGGAGG	5
	OT-4	chr2	197121373	197121396	+	ACACAAAG CACCATGA GTGGAGG	2
	OT-5	chr3	2220065	2220088	-	GGAGAAAA CACTCTGA GTGATGG	5
	OT-6	chr6	159803440	159803463	-	GCAGAGGG CAGAGTGA GTGGAGG	5
	OT-7	chrX	124413658	124413681	+	GCATAAAG CACAAGGA GAGGTGA	5
Cosmid+NGS	OT-1	Chr 5	178282798	178282820	+	AAAGGAAG CACCATGA GTGGGGG	3

Table S2. Sequencing results of the translocation detected by CAST-seq in the BM of SU-3 HSPC transplanted mice

chr	start	end	read (n)	hits (n)	read (n) ctrl	hits (n) ctrl	group	annotation	Distance To TSS	Gene	Genes within 100kb	Oncogenes within 100kb
**X**	48674308	48687957	16465	1974	2917	488	OMT	Promoter (<=1kb)	0	WAS	GLOD5; RBM3; WDR13 ; SUV39H1; WAS	WAS
**17**	19697297	19697797	257	34	3	3	NBS	Intron (ENST00000325411.9/146802, intron 13 of 16)	21182	SLC47A2	SLC47A2; ALDH3A1; ALDH3A2 ; ULK2	

Table S3. List and description of AAV integration sites retrieved in this study.

For each IS matrix there are the following columns:
chr: chromosome number
integration_locus: genomic position of the integration sites
GeneID by RefSeq RefSeq Gene Symbol of the closest gene targeted by vector insertion
Gene strand: genomic orientation of the targeted gene
MhOR/ins: Micro-Homology Region (MhOR): Nucleotides overlap between alignments of AAV and target genome (indicated as negative number) / insertion (ins): De novo insertion of nucleotides between AAV and host genome breakpoint (indicated as positive number)
Junction: nucleotide position on the AAV genome at the vector/cellular genome breakpoint
vector_fragments: number of AAV fragments identified within the vector portion of the reads
sample_columns: column name contained the sample ID, the analyzed tissue, the PCR-set from which the IS was identified and eventually the vector dose. Within the column is indicated the number of different genomic fragments identified for each IS

Table S4. Most targeted genes by AAV integration.

ALL IS N= 164			
chr	GeneID	Targeting	%
chrX	WAS	16	9.76
chr1	SMPDL3B	7	4.27
chr1	KDM4A	3	1.83
chr2	DHFRP3	3	1.83
chr10	VSIR	2	1.22
chr12	GRIP1	2	1.22
chr13	PCDH17	2	1.22
chr15	LRRK1	2	1.22
chr18	ZNF521	2	1.22
chr19	MRI1	2	1.22
chr20	CTNNBL1	2	1.22
chr3	FHIT	2	1.22
chr3	MIR548AB	2	1.22
chr5	MIR4456	2	1.22
chr6	EEF1A1	2	1.22
**CD34 N=115**			
chr	GeneID	Targeting	%
chrX	WAS	10	8.7
chr1	SMPDL3B	3	2.61
chr1	KDM4A	3	2.61
chr10	VSIR	2	1.74
chr12	GRIP1	2	1.74
chr2	DHFRP3	2	1.74
chr20	CTNNBL1	2	1.74
chr3	FHIT	2	1.74
chr5	MIR4456	2	1.74
**BM=57**			
chr	GeneID	Targeting	%
chrX	WAS	6	10.71
chr1	SMPDL3B	4	7.14
chr13	PCDH17	2	3.57
chr18	ZNF521	2	3.57
chr6	EEF1A1	2	3.57

Table S5. AAV integration sites abundance in SU samples in vitro (relative to Figure 6G)

SU-1 CD34			SU-2CD34			SU-3 CD34			SU-4 CD34		
GeneID	%	Cell count	GeneID	%	Cell count	GeneID	%	Cell count	GeneID	%	Cell count
IL7R	30.4	38	SHANK2	6.7	6	ATP6V1C2	30.9	17	WAS	8.2	6
PCDH17	10.4	13	WAS	5.6	5	LINC01608	7.3	4	WAS	8.2	6
ZBTB38	8	10	WAS	4.5	4	SMPDL3B	5.4	3	NUP88	6.8	5
SMPDL3B	4	5	LRRK1	3.4	3	TRPM4	5.4	3	LOC100507468	5.5	4
ELMO1	3.2	4	CLTC	3.4	3	OBSCN	3.6	2	C3orf58	4.1	3
KIF17	2.4	3	CTNNBL1	3.4	3	VSIR	3.6	2	LOC101928820	4.1	3
KDM4A	2.4	3	WAS	3.4	3	TUB	3.6	2	KDM4A	2.7	2
ITIH5	2.4	3	RABGGTB	2.2	2	GRIP1	3.6	2	LINC00466	2.7	2
GRIP1	2.4	3	SEC22B	2.2	2	C15orf53	3.6	2	PKN2-AS1	2.7	2
WAS	2.4	3	VSIR	2.2	2	GP2	3.6	2	EVI5	2.7	2

Table S6. AAV integration sites abundance tracking in vivo for SU-1 (relative to Figure 6H)

**SU-1 CD34**			**SU-1 17BM**			**SU-1 21BM**		
GeneID	%	Cell count	GeneID	%	Cell count	GeneID	%	Cell count
IL7R	30.4	38	IL7R	42.6	230	WAS	24.3	51
PCDH17	10.4	13	EEF1A1	34.6	187	IL7R	20	42
ZBTB38	8	10	NCF2	8.2	44	EEF1A1	11.9	25
SMPDL3B	4	5	HFM1	2.4	13	ZBTB38	4.8	10
ELMO1	3.2	4	CLPB	1.7	9	PCDH17	3.3	7
KIF17	2.4	3	ZNF521	1.3	7	DAD1	3.3	7
KDM4A	2.4	3	ZBTB38	1.1	6	ELMO1	3.3	7
ITIH5	2.4	3	EEF1A1	1.1	6	WAS	2.4	5
GRIP1	2.4	3	ELMO1	1.1	6	JPH3	1.9	4
WAS	2.4	3	WAS	0.9	5	MRI1	1.9	4

Table S7. AAV integration sites abundance tracking in vivo for SU-3 (relative to Figure 6I)

SU-3 CD34			SU-3 27BM			SU-3 28BM		
GeneID	%	Cell count	GeneID	%	Cell count	GeneID	%	Cell count
ATP6V1C2	30.91	17	SULT1C2P1	65	13	DDX18	26.67	4
LINC01608	7.27	4	ATP6V1C2	20	4	ECHDC2	20	3
SMPDL3B	5.45	3	WAS	15	3	SMCO4	13.33	2
TRPM4	5.45	3				C2CD4C	13.33	2
OBSCN	3.64	2				ATP6V1C2	13.33	2
VSIR	3.64	2				WAS	13.33	2
TUB	3.64	2						
GRIP1	3.64	2						
C15orf53	3.64	2						
GP2	3.64	2						
